# Allosteric Inhibitors of Macrophage Migration Inhibitory Factor (MIF) Interfere with Apoptosis-Inducing Factor (AIF) Co-Localization to Prevent Parthanatos

**DOI:** 10.1021/acs.jmedchem.3c00397

**Published:** 2023-06-23

**Authors:** Deng Chen, Angelina Osipyan, Jeaunice Adriana, Mohammed Kader, Maxim Gureev, Catharina W. J. Knol, Marie-Cathérine Sigmund, Zhangping Xiao, Petra E. van der Wouden, Robbert H. Cool, Gerrit J. Poelarends, Frank J. Dekker

**Affiliations:** † Department of Chemical and Pharmaceutical Biology, Groningen Research Institute of Pharmacy, University of Groningen, Antonius Deusinglaan 1, 9713 AV Groningen, The Netherlands; ‡ Center of Chemo- and Bioinformatics, Institute of Biodesign and Complex Systems Modeling, I. M. Sechenov First Moscow State Medical University, 119991 Moscow, The Russian Federation

## Abstract

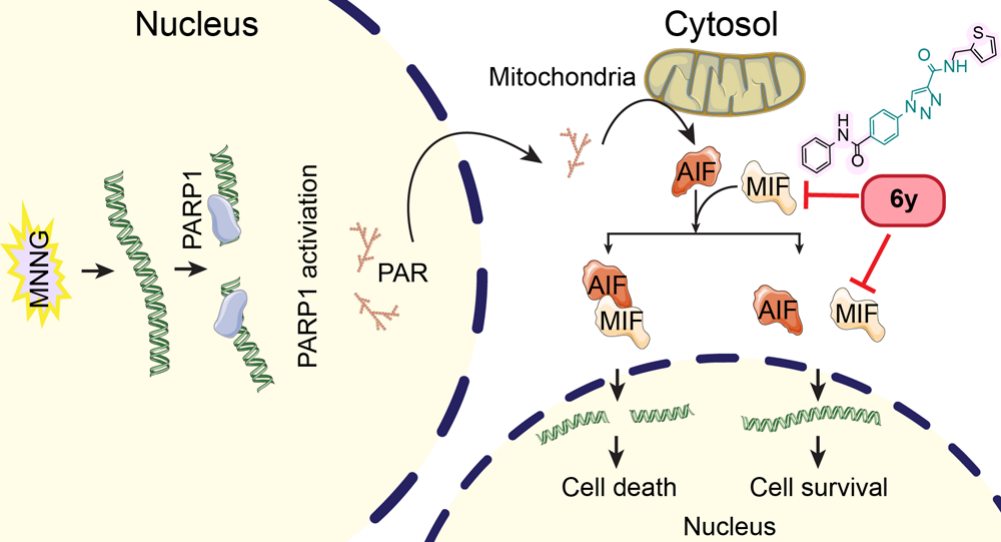

Macrophage migration inhibitory factor (MIF) is a multifunctional cytokine and essential signaling protein associated with inflammation and cancers. One of the newly described roles of MIF is binding to apoptosis-inducing factor (AIF) that “brings” cells to death in pathological conditions. The interaction between MIF and AIF and their nuclear translocation stands as a central event in parthanatos. However, classical competitive MIF tautomerase inhibitors do not interfere with MIF functions in parthanatos. In this study, we employed a pharmacophore-switch to provide allosteric MIF tautomerase inhibitors that interfere with the MIF/AIF co-localization. Synthesis and screening of a focused compound collection around the 1,2,3-triazole core enabled identification of the allosteric tautomerase MIF inhibitor **6y** with low micromolar potency (IC_50_ = 1.7 ± 0.1 μM). This inhibitor prevented MIF/AIF nuclear translocation and protects cells from parthanatos. These findings indicate that alternative modes to target MIF hold promise to investigate MIF function in parthanatos-mediated diseases.

## Introduction

Parthanatos is a form of caspase-independent programmed cell death resulting from the accumulation of poly (ADP-ribose) (PAR) polymers and is characterized by a unique pathway that is distinct from apoptosis, necroptosis, and any other type of cell death. Parthanatos is involved in a wide range of diseases, such as ischemic stroke,^1^ glutamate excitotoxicity,^2^ inflammation,^3^ reactive oxygen species (ROS)-related damage,^4^ cancers,^5^ heart attack, retinal disease, diabetes,^6^ as well as Parkinson’s disease and other neurodegenerative diseases.^7^ The term *parthanatos* was named after *Thanatos*, the personification of death in Greek mythology, to refer to PAR-mediated cell death.^8^ The parthanatos cascade involves PAR polymerase 1 (PARP-1) overactivation, PAR accumulation, PAR binding to the death effector apoptosis-inducing factor (AIF), AIF release from the mitochondria, and its nuclear translocation.^9^ AIF is a mitochondrial oxidoreductase that participates in the biogenesis of the respiratory chain in physiological conditions. In parthanatos, AIF induces chromatin condensation and DNA fragmentation, although the biochemical events mediating this nuclear-mitochondrial crosstalk are not entirely elucidated. In 2016, macrophage migration inhibitory factor (MIF) was identified as a crucial factor in the induction of parthanatos by forming a MIF/AIF complex that translocates from the cytosol to the nucleus triggering DNA fragmentation and cell death. Considering that AIF does not possess any nuclease activity, MIF was assigned as a parthanatos-associated AIF nuclease (PAAN).^10,11^ The research concept to interfere with this interaction to prevent parthanatos using the peptide-like inhibitor PAANIB-1 was suggested by Dawson in 2022.^12^ Overall, discovering a role for the MIF/AIF interaction in PARP-1-mediated cell death indicates a potential for small-molecule modulation of this interaction.

**Figure 1:**
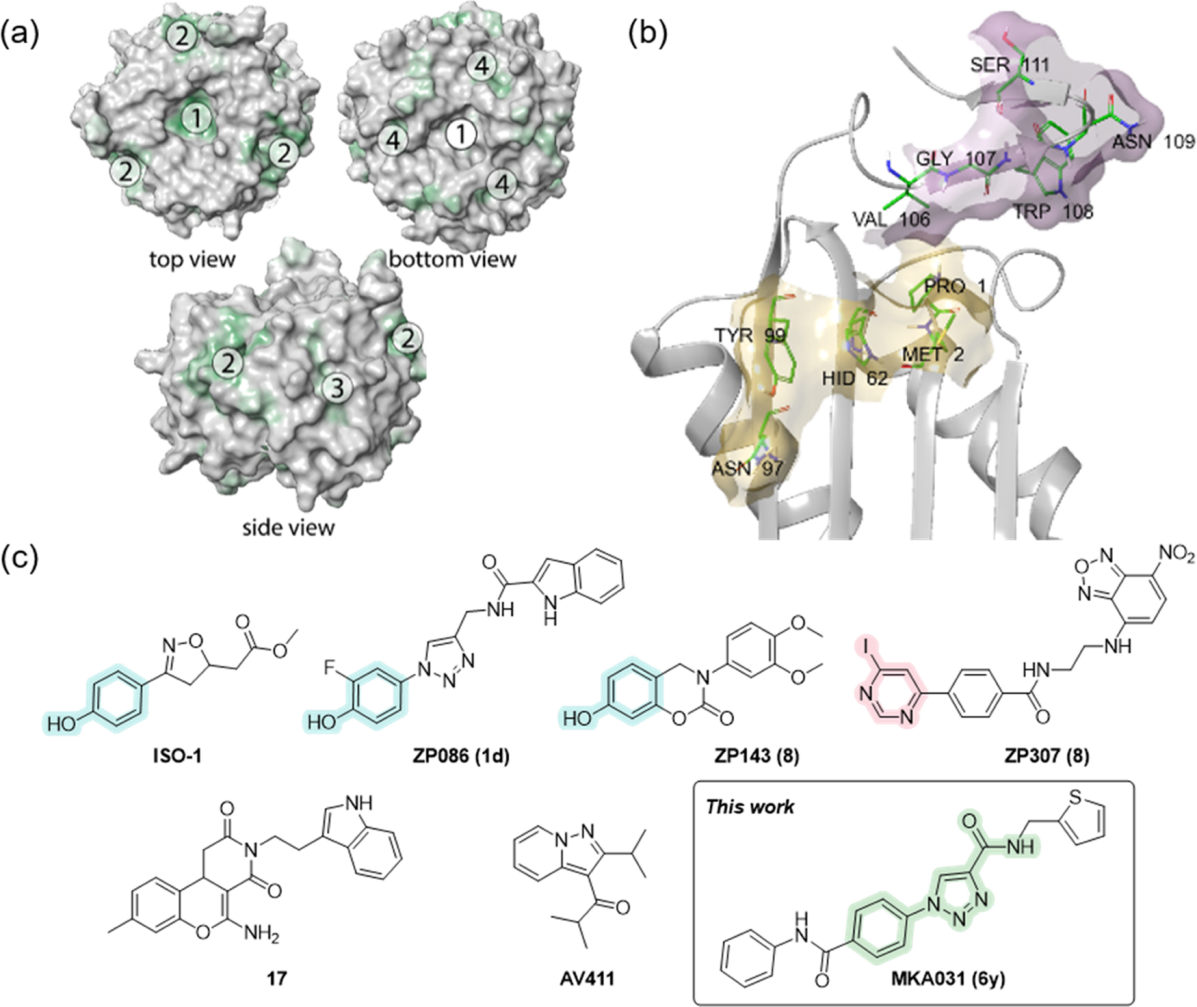
Overall topology and small-molecule inhibitors of MIF. (a) Hydrophobic surface area available for interactions with proteins and small molecules (PDB: 1GD0(32)). (1) Central part of the trimer, central solvent channel. (2) Cavities between trimer subunits on the outside of the MIF protein. Several publications list them as allosteric and CD74-binding sites.^33,34^ (3) Sides of each monomer between the α-helices formed by Phe18-Ala29 and Ser74-Arg86. This sequence is rich in leucine and proline and responsible for the formation of the lipophilic leucine zipper-like structure. (4) “Pseudo-(E)LR” motif, as the glutamate (Glu/E) was substituted with an aspartic acid (Asp/D) which allows interaction with CXCR2, due to structural homology to its ligand CXCL8. (b) Orthosteric site of MIF (PDB: 1GD0(32)): yellow—the site responsible for the tautomerase activity, plum—site of CD74 activation. Observed sites correspond with hydrophobic zones 1 and 2 in (a). (c) Reported MIF inhibitors with different binding modes. **ISO-1**,^35^**ZP086**,^36^ and **ZP143**(37) are competitive inhibitors. **ZP307**(38) is a covalent inhibitor, and **17**(39) and **AV411**(22) are allosteric inhibitors. In this study, we report a new noncompetitive inhibitor **6y** (**MKA031**).

In 1966, MIF was initially identified as a lymphokine derived from activated T-cells that inhibited the random migration of macrophages.^13^ Currently, we know MIF as a widely secreted pleiotropic cytokine that is involved in multiple processes.^14−16^ MIF has a homotrimeric structure, and each monomer consists of 115 residues of 12.5 kDa, forming a β-α-β-fold typical for the tautomerase superfamily.^17^ One aspect of MIF family proteins that remains enigmatic is the presence of catalytic sites, such as the tautomerase active site that requires the *N*-terminal proline^18^ and the oxidoreductase active site that requires Cys56 and Cys59.^19−21^ Although no physiological substrate has been identified for the MIF tautomerase active site, the “pseudosubstrates” phenylpyruvate (PP) or 4-hydroxyphenylpyruvate (4-HPP) proved to be suitable to screen MIF binders that influence MIF tautomerase activity.^21,22^ Furthermore, MIF has been shown to harbor both 3′ exonuclease and endonuclease activity independent of its oxidoreductase and tautomerase activities.^11^ Moreover, MIF is known to be involved in protein–protein interactions. The hydrophobic surface area of MIF has 4 potential binding sites (Table S3) that stand out due to their hydrophobic contact agglomeration available for interactions with proteins and small molecules (Figure 1a). These interaction sites enable, for example, binding to and activation of membrane receptors, such as the cluster of differentiation 74 (CD74)^23^ (Figure 1b) and C-X-C motif chemokine receptors 2,^24,25^ 4,^25^ and 7^26^ (CXCR2, 4, and 7). Protein–protein interactions also facilitate intracellular MIF activities in cell signaling and gene transcription. For example, MIF interacts with thioredoxin (TRX) to induce nuclear factor kappa light chain enhancer of activated B cell (NF-κB)-mediated signaling.^27^ Furthermore, MIF forms a complex with p53 to attenuate p53-mediated gene transcription^28^ and coordinate the cell cycle with DNA damage checkpoints.^29^ Overall, the results demonstrate a role for MIF protein–protein interactions in various disease models.^30,31^ The development of small-molecule modulators of MIF protein–protein interactions holds promise for chemical–biological investigation of MIF function as well as drug discovery.

The most studied small-molecule MIF inhibitors, often containing a phenolic core (Figure 1c, **ISO-1**, **ZP086**, **ZP143**), reversibly target the tautomerase active site. Alternatively, covalent inhibitors containing a heterocyclic electrophilic fragment (Figure 1c, **ZP307**) modify its catalytic base Pro1.^40^ In 2020, it was found that MIF has an allosteric gating residue of a solvent channel Tyr99 which regulates both the MIF enzymatic activity and CD74 activation.^41^ Tyr36 was found to be a multifunctional residue allowing small molecules to bind to the side chain in the active site or allosteric site.^22^ However, the connection between the MIF catalytic site and the structural basis of the MIF/AIF^11^ or MIF/ssDNA^42^ interaction in the allosteric site is unknown.

Expanding on MIF tautomerase inhibition, we became inspired by the idea to assemble a focused compound collection around the 1,2,3-triazole scaffold,^43,44^ as found in **ZP086** (**1d**),^36^ in which the phenolic hydroxyl functionality is removed (Figure 1c). The rationale for this strategy is to eliminate the key interaction of the phenol with Asn97 on the bottom of the tautomerase active site. Eliminating this interaction point would enable binding of the inhibitor further away from the tautomerase active site (Figure 1b). The chemistry of triazoles appears very attractive to assemble a diverse compound collection and is frequently used in pharmaceuticals.^45^ The copper(I)-catalyzed alkyne-azide 1,3-dipolar cycloaddition (CuAAC),^46^ commonly known as the “click” reaction, was successfully applied by Jorgensen^43^ to synthesize a series of competitive MIF tautomerase inhibitors and encouraged us to design a library of 4-substituted triazole-phenols to study further MIF inhibition (Figure 1c, **ZP086**).^36^ In this work, we use triazoles as a structural core of MIF inhibitors preventing the interaction with the orthosteric active site by exchanging the phenolic triazole scaffold for a nonphenolic one.

Overall, in the present study, we report the discovery of a new class of allosteric MIF inhibitors with a 1-phenyl-1*H*-1,2,3-triazole-4-carboxamide scaffold. We synthesized a focused compound collection of 25 triazoles that lack the aromatic alcohol pharmacophore feature. Screening of the compound collection for inhibition of MIF tautomerase activity using PP as a substrate provided inhibitor **6y** (**MKA031**) as the most active one with an IC_50_ = 1.7 ± 0.1 μM. Enzyme kinetic analysis indicated binding to an allosteric binding site that inhibits MIF tautomerase activity. Further characterization indicated that **6y** is able to interfere with the MIF/AIF interaction, MIF nuclear translocation, and MNNG-induced parthanatos. Altogether, this demonstrates the potential of targeting MIF binding sites that are allosteric to the MIF tautomerase active site for interfering with intracellular MIF functions.

## Results and Discussion

### Synthesis of Triazole Compound Collection

**Figure 2:**
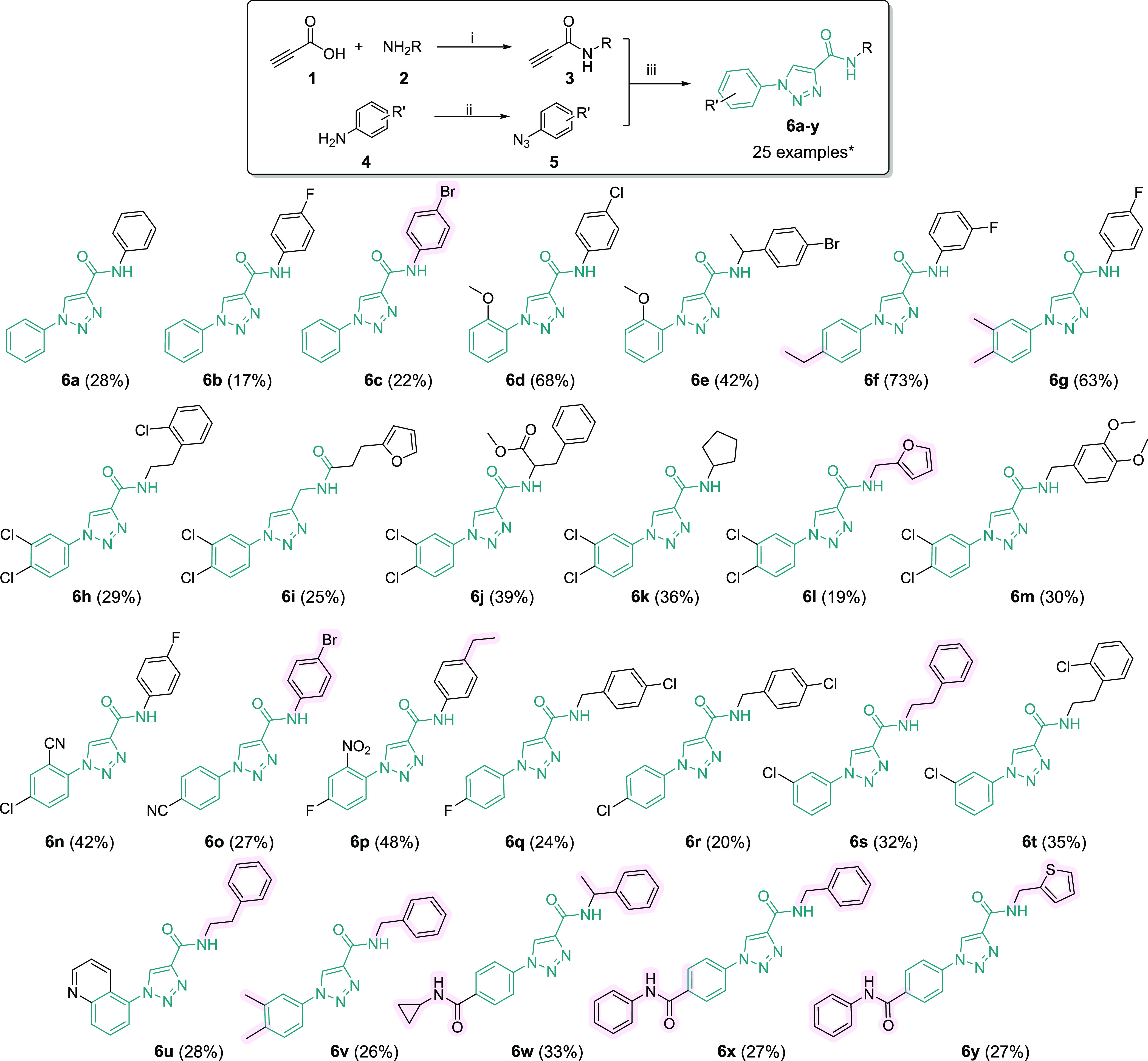
Synthesis of allosteric MIF inhibitors with 1-phenyl-1*H*-1,2,3-triazole-4-carboxamide core. Reagents and conditions: (i) DCC, acetonitrile, rt, 2 h; (ii) (a) HBF_4_, NaNO_2_, H_2_O, rt, 1 h; (b) NaN_3_, rt, 1 h; (iii) CuSO_4_*5H_2_O, sodium ascorbate, MeOH/H_2_O, rt, overnight. *Compound **6i** was prepared using general procedure B for amide synthesis.

The synthesis of the focused compound collection involves, essentially, a three-step procedure, as depicted in Figure 2. The CuAAC reaction was performed by adding pre-catalyst copper(II) sulfate and a reducing agent sodium ascorbate to the alkyne and azide substrates solution in methanol at room temperature (rt). The terminal alkynes as synthetic precursors were prepared by coupling propiolic acid to aromatic and aliphatic amines using *N,N*′-dicyclohexylcarbodiimide (DCC) as an activating agent. Azides were produced by converting different anilines into diazonium salts *in situ* and subsequent reaction with sodium azide in aqueous conditions to provide the corresponding aryl azides. For the preparation of **6i**, the carboxylic acid was coupled to propargylamine using DCC-mediated amidation, providing the substituted terminal alkyne used in the “click” reaction. The corresponding alkynes and azides were employed in the CuAAC reaction to provide a series of 25 compounds in low to moderate overall yields (17–63%) after purification using flash chromatography.

### Inhibition of MIF Tautomerase Activity

Recombinant human MIF with a C-terminal His-tag was expressed and purified following methods published previously.^47^ The inhibitory potency for MIF tautomerase activity of the 1-phenyl-1*H*-1,2,3-triazole-4-carboxamides was measured after pre-incubation with MIF. The MIF-catalyzed PP tautomerization was measured by the corresponding change in UV absorbance over the first 3 min of the reaction.^48^ Initial screening of the inhibitory potency was performed at 10 μM inhibitor concentration. The compounds **ZP143** and **ISO-1** (competitive MIF tautomerase inhibitors, Figure 1c) were included as controls and showed potency comparable to those reported before.^37^

**Figure 3:**
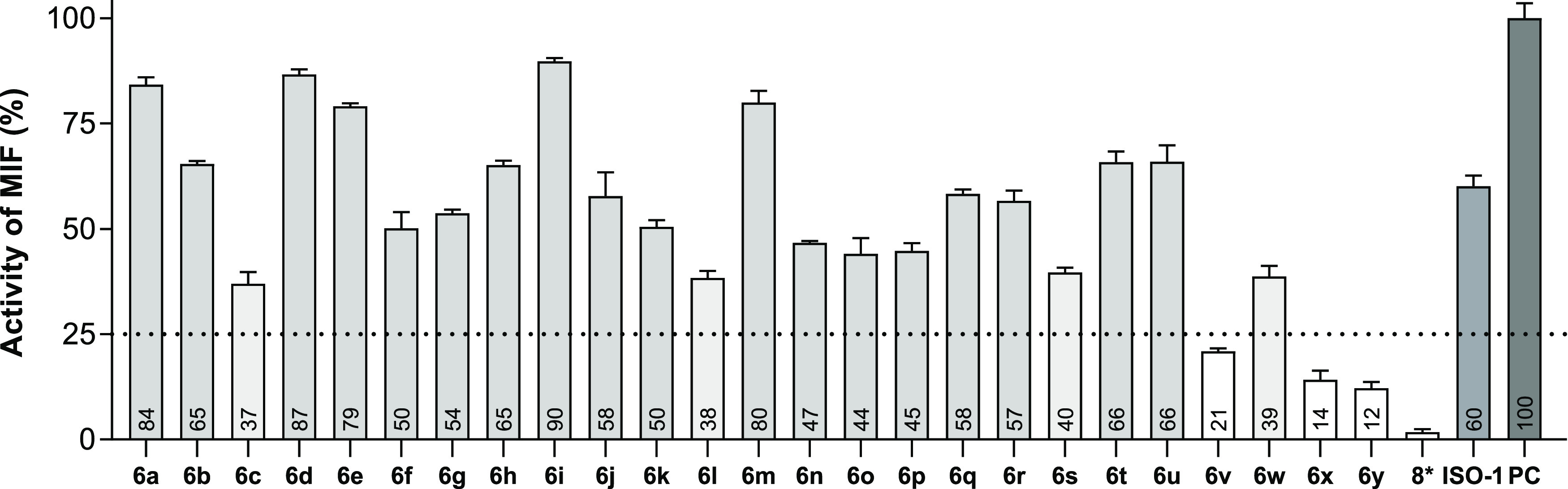
MIF tautomerase activity assay. Screening of the inhibitory potency of the triazole-based collection at 10 μM inhibitor concentration. The enzyme activity in the absence of the inhibitors was set to 100% as a positive control (PC). The signal in the absence of the enzyme was set to 0%. Data are presented as mean ± SD, *n* = 3. 8* is compound **ZP143**.

The compounds that have demonstrated more than 75% of inhibition toward MIF tautomerase activity (Figure 3) were subjected to IC_50_ determination. For **6v** an IC_50_ of 6.5 ± 0.4 μM was determined; **6x** and **6y** showed an IC_50_ of 2.5 ± 0.1 and 1.7 ± 0.1 μM, respectively. Altogether, we concluded that inhibitor **6y** was the most active in this series. The compound collection of 1-phenyl-1*H*-1,2,3-triazole-4-carboxamides showed curious structure-activity relationships for inhibition of MIF tautomerase activity (Figure 2). Surprisingly, carboxamide-substituted *N*-aryl fragment (Figure 2, **6x** and **6y**) showed the most appreciable increase toward the tautomerase activity of MIF in this series. The potency gain can be explained by the presence of two amide bonds and their ability to mimic peptide bonds. The substitution of benzyl into thiophenyl fragment slightly increased the activity giving the final touch to **6y**. Unfortunately, none of the chloro-containing *N*-arylated triazoles showed advanced activity, likewise nitro-, cyano- and methoxy- groups. In contrast, bromo (Figure 2, **6c**, **6o**) and alkyl substitution (Figure 2, **6g**, **6f**, **6w**) on *N*-aryl fragment were the most successful. On the other side of the molecule, we utilized carboxamide functionalization, which was initiated by changing the starting amine.

**Figure 4:**
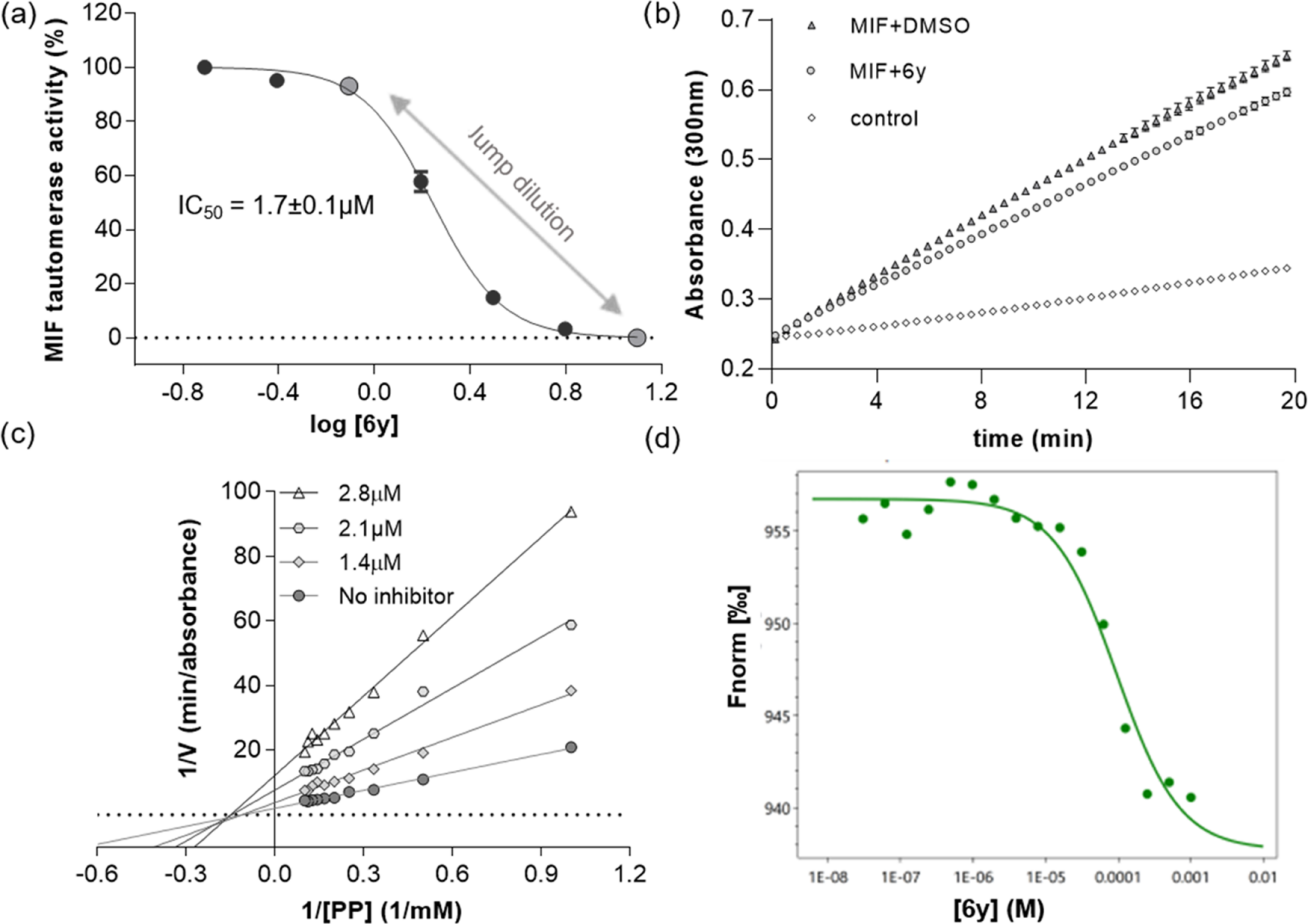
Kinetic parameters of 6y binding to MIF. (a) Dose–response curve for inhibition of MIF tautomerase activity by **6y**. (b) Jump dilution progress curve for **6y**. (c) Lineweaver–Burk plot of MIF inhibition by **6y** at the concentrations 0, 1.4, 2.1, and 2.8 μM. We determined that inhibition was noncompetitive. (d) Microscale thermophoresis (MST) of the binding affinity of **6y** to MIF.

We found that *N*-aryl carboxamides (Figure 2, **6a**–**6d**, **6f**, **6g**, **6n**–**6p**) did not show improved activity compared with alkyl- (including phenyl-) substitution. Incorporating heterocyclic functionality (Figure 2, **6l**) increased potency twice compared to starting **6a**. Despite our initial expectations, the nonsubstituted benzyl group of carboxamide (Figure 2, **6v**, **6x**) significantly improved the activity toward MIF (compared to **6a**) by breaking the planarity of the molecule by adding sp^3^ carbon giving the final molecules drug-like features. Hydrogen bonding, in this case, fades into the background and gives way to the π-π interactions or hydrophobic contacts due to the hydrophobicity of the Leu- and Ile-rich putative allosteric site or its aromatic surrounding (Tyr36, Phe49, Tyr75, Tyr95, Trp108, and Phe113). Thus, the increased activity of the compound **6y** can be explained by enhanced π-stacking that improves site-specific binding. Altogether, using the primary screening with MIF tautomerase assay (Figure 3) and IC_50_ measurements, we selected the compound, **6y**, with a low micromolar IC_50_ (Figure 4a) for further characterization.

To investigate the reversibility of binding, we performed the activity recovery experiment based on pre-incubation of MIF with the inhibitor at saturated concentration followed by jump dilution. The experiment aimed to study the reversibility of the MIF complex with **6y**; thus, MIF was preincubated with 25 μM **6y** for 10 min before 20-fold dilution and then tested for residual enzyme activity. After 20 min of residence time, MIF tautomerase activity recovered comparably to that expected for instantaneously reversible inhibitors (Figure 4b). The results indicate that the binding of **6y** to MIF is reversible.

Enzyme kinetics experiments were performed to determine the mode of MIF inhibition by **6y** (SI, Table S1). The rate of MIF-catalyzed PP conversion was measured at concentrations ranging from 0 to 10 mM in the absence or presence of various inhibitor concentrations (0, 1.4 μM, 2.1 μM, or 2.8 μM). The Lineweaver–Burk plot (Figure 4c) indicated that in the presence of 3 different inhibitor concentrations, the *K*_m_ remains constant between 6.0 ± 1.1 and 6.5 ± 0.7 compared to 6.2 ± 0.8 mM for the control. Conversely, *V*_max_ (and *V*_max_/*K*_m_, subsequently) is decreasing from 0.40 ± 0.03 to 0.08 ± 0.01 absorbance/min, demonstrating that binding of **6y** to MIF is noncompetitive (Figure 4c).

A microscale thermophoresis (MST) assay was performed to confirm the binding of **6y** to MIF. This experiment provided a binding curve with a *K*_d_ of 95 μM (Figure 4d). We note that deviations from the sigmoidal distribution of the MST curve start to appear at concentrations higher than 1000 μM, which can be attributed to the limited solubility of **6y** at these concentrations. Overall, we concluded that **6y** binds reversibly to an allosteric binding site that affects MIF tautomerase activity.

### Compound 6y Prevents MNNG-Induced Cell Death

**Figure 5:**
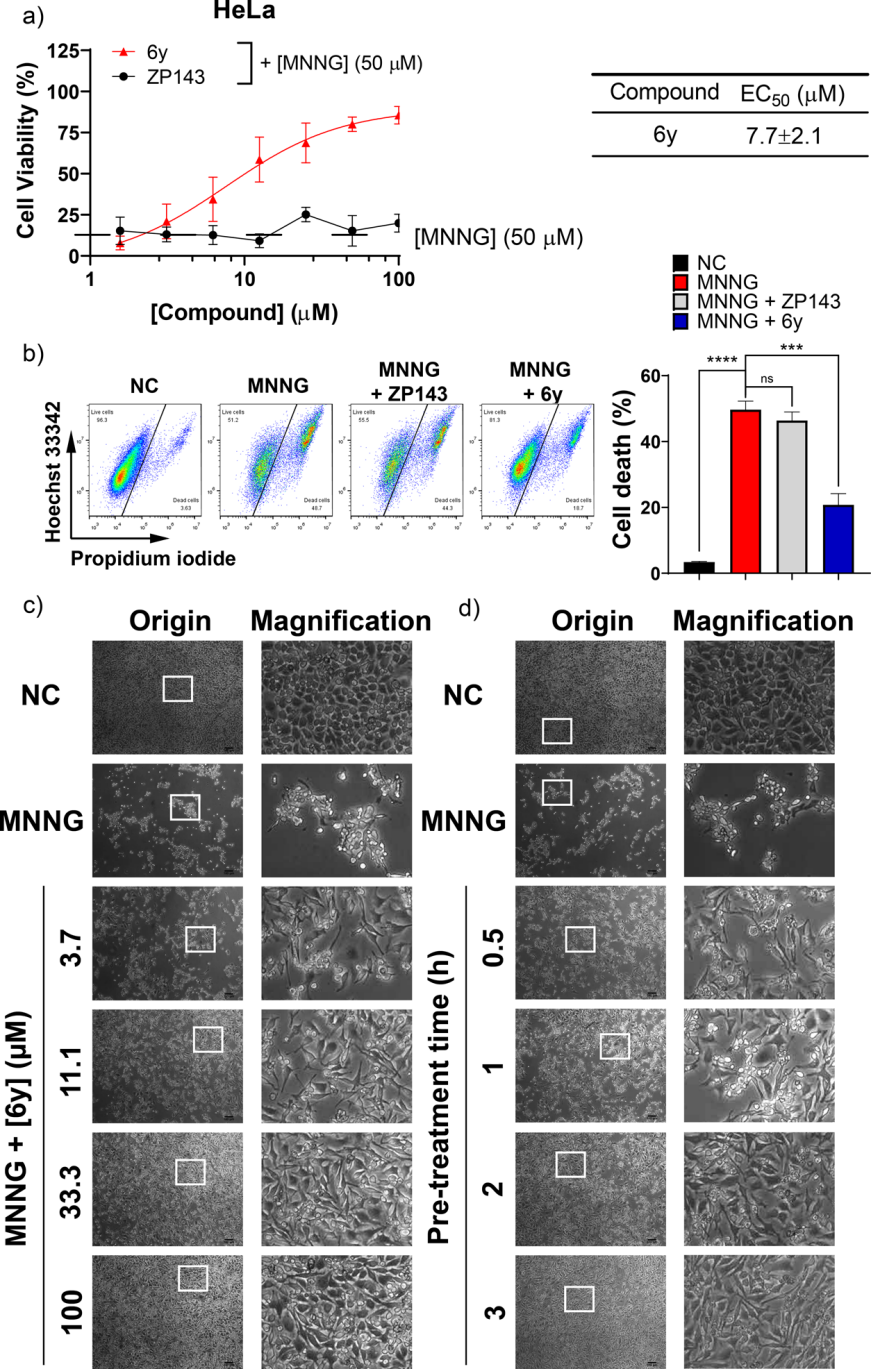
Allosteric MIF inhibitor 6y prevents parthanatos. (a) Quantification of protective effects of allosteric MIF inhibitor **6y** and competitive MIF inhibitor **ZP143** in parthanatos by MTS assay. (b) **6y** rescued cells from MNNG treatment. HeLa cells were seeded in a 6-well plate and preincubated with **6y**. Later on, HeLa cells were treated with either a blanc DMSO dilution as negative control (NC) or MNNG (50 μM, 15 min) to induce parthanatos. After 24 h incubation, the cells were harvested and dual stained with Hoechst 33342 and propidium iodide. Live and dead cells were determined by FACS. Representative images of parthanatic HeLa cells protected by **6y** in a (c) dose- and (d) pretreatment time-dependent manner.

Targeting the MIF/AIF or the MIF/ssDNA interaction has the potential to inhibit parthanatos-mediated cell death (Figure 5). Therefore, we explored the effect of the MIF allosteric inhibitor **6y** in a model of parthanatos. In this model, the DNA alkylating agent N-methyl-N′-nitro-N-nitrosoguanidine (MNNG) is used to initiate cell death in a caspase-independent manner by causing PARP-1 overactivation and subsequent cell death. To compare the effect of **6y**, we used competitive MIF tautomerase inhibitor **ZP143** (*K*_i_ = 0.10 ± 0.01 μM)^37^ as a control. Remarkably, **6y** protected HeLa cells from MNNG-induced parthanatos (Figure 5a) in a dose-dependent manner with an EC_50_ of 7.7 ± 2.1 μM, whereas **ZP143** had no protective effect. We further investigated the effect on cell viability using flow cytometry by double-staining the cells with Hoechst 33342 and propidium iodide (PI) to distinguish between live and dead cells (Figure 5b). The staining pattern resulting from the simultaneous use of these dyes showed that PI stained 20% of cells in the MNNG and **6y** co-treated group and 50% in the MNNG-treated group.

These data indicate that treatment with **6y** conferred cells with resistance to parthanatos compared to MIF inhibitor **ZP143**. In addition, cellular morphology was investigated to see if the MIF inhibitor **6y** restores the original phenotype. Our observation shows that nontreated cells adhere to the bottom of the plate and show a round phenotype, while MNNG treatment disturbs this adherent phenotype (Figure 5c,d). Upon pretreatment with MIF inhibitor **6y**, the original phenotype was mainly (but not completely) restored after treatment with MNNG in a dose- and time-dependent manner. Taken together, these data indicate that the allosteric MIF tautomerase inhibitor **6y** effectively counteracts the induction of parthanatos, whereas the competitive MIF tautomerase inhibitor cannot do so.

### Compound 6y Protects Genomic DNA by Blocking the Recruitment of MIF to AIF

**Figure 6:**
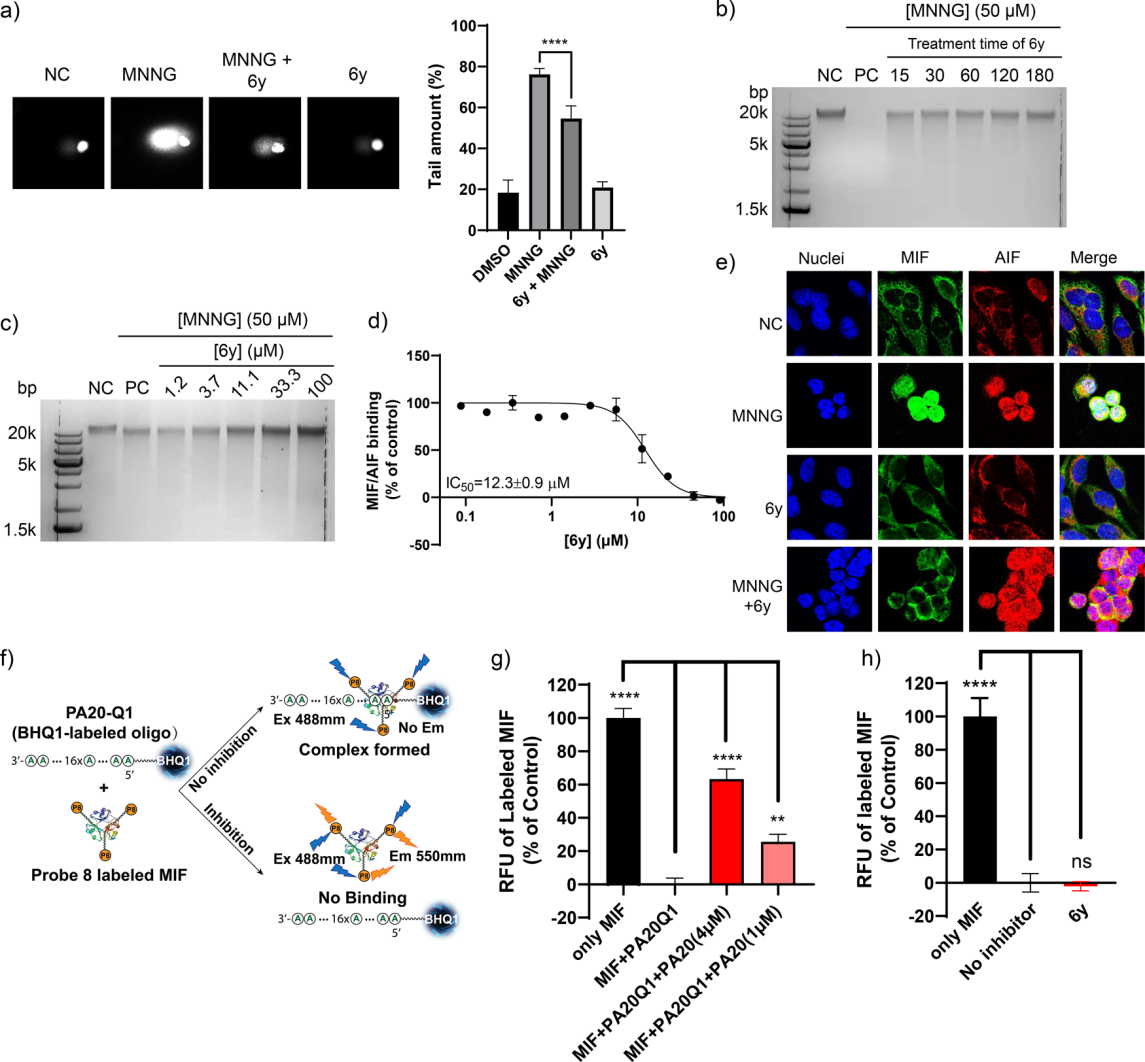
**6y** protects genomic DNA via interfering with MIF/AIF binding and not with MIF/DNA binding. (a) **6y** prevented MNNG-induced genome damage (comet assay). (b, c) MIF inhibitor **6y** demonstrated protective effects on the genome in parthanatos in a dose- and time-dependent manner in cells. Treatment with a blank DMSO dilution is the negative control (NC). Treatment with an MNNG dilution is the positive control (PC). (d) MIF inhibitor **6y** inhibited MIF/AIF binding in vitro (ELISA assay). (e) MIF inhibitor **6y** (100 μM) blocked MIF nuclear translocation upon MNNG stimulation, while AIF nuclear translocation was uninfluenced. (f) Schematic representation of the MIF/ssDNA binding assay. **Probe 8** is compound **ZP307** (Figure 1c). (g) PA20 attenuated the binding of MIF and PA20-Q1. (h) **6y** was unable to block MIF/PA20-Q1 binding.

We tested if **6y** interfered with the parthanatic cell death pathway to elucidate how MIF inhibitors protect cells from parthanatos. Recently it was proved that upon activation of parthanatos, AIF mediates MIF translocation to the nuclei, while MIF fragments genomic DNA and consequently causes cell death.^11^ Therefore, we investigated how inhibitor **6y** affected the integrity of genomic DNA using the comet and gel electrophoresis assay described previously.^11^ The comet assay was performed under alkaline conditions to detect both single- and double-strand breaks in the genome. Through the comet assay, we found that MNNG-induced DNA damage was inhibited by **6y** (Figure 6a). The tail of the cell comet was smaller in the **6y** protected group compared to the MNNG group. Compound **6y** itself did not affect genome integrity. Moreover, gel electrophoresis demonstrated that a protective effect is associated with the pretreatment with the MIF inhibitor in a dose and time-dependent manner (Figure 6b,c). Together, these data showed that pretreating cells with **6y** protected genomic DNA from fragmentation in parthanatos.

Next, we investigated the mechanism by which **6y** protects genome DNA. Considering that AIF-mediated MIF nuclear translocating is a crucial step in parthanatos, we tested if **6y** interferes with MIF/AIF binding *in vitro*. An ELISA assay enabled the detection of the MIF/AIF interaction and showed that inhibitor **6y** inhibits MIF/AIF binding with an IC_50_ of 12.3 μM (Figure 6d). Next, confocal microscopy pictures (Figure 6e) demonstrated that **6y** is able to interfere with the MIF/AIF localization in MNNG-treated cells. Under control conditions, MIF and AIF are mainly located in the cytosol. Upon MNNG treatment, both MIF and AIF translocated to the nucleus. MIF inhibitor **6y** did not affect MIF localization under control conditions. Co-treatment with MNNG and **6y** demonstrated that MIF remained mainly in the cytoplasm while AIF moved to the nucleus. Taken together, these data indicate that **6y** can interfere with the MIF/AIF interaction and blocks MIF nuclear translocation.

Although in our hands the nuclease activity could not unambiguously be assigned to MIF (due to the possibility of bacterial contamination), there is still a possibility that MIF binds to single-stranded DNA (ssDNA). Furthermore, the direct interaction between MIF and ssDNA was investigated. We developed a MIF/ssDNA binding assay to detect and quantify the binding of MIF to ssDNA (Figure 6f). To establish this assay, we employed a MIF probe **ZP307** (Figure 1c) that binds covalently to the *N*-terminal proline of MIF. This probe enables the fluorescent labeling of MIF.^38^ The oligonucleotide Poly A was earlier described as a MIF nuclease substrate, lacking a secondary structure.^11^ Also, a black hole quencher-1 (BHQ1) at the 5′ terminal of the poly-A oligonucleotide cannot be cleaved by MIF.^11^ This enabled using the BHQ1-labeled poly A oligonucleotide (PA20-Q1) as an energy acceptor to quench fluorescence emission by labeling MIF once a MIF/ssDNA complex formed (Figure 6f). The unlabeled oligonucleotide, PA20, impaired the binding of MIF/PA20-Q1 in a dose-dependent manner (Figure 6g). The attenuation effect of PA20 is achieved by competing with PA20-Q1 binding to IF because the two oligonucleotides have the same binding site on MIF. These data indicate that if a compound competitively binds to MIF at PA20-Q1 binding site, the fluorescence signal of MIF will be restored. However, **6y** could not replace the oligonucleotide at a high concentration, up to 100 μM (Figure 6h), suggesting that **6y** does not directly interfere with MIF/ssDNA binding. Taken together, these data demonstrated that **6y** protects genomic DNA from damage by blocking AIF-mediated MIF nuclear translocation rather than attenuating the DNA binding ability of MIF.

### MIF/AIF Interaction Model

**Figure 7:**
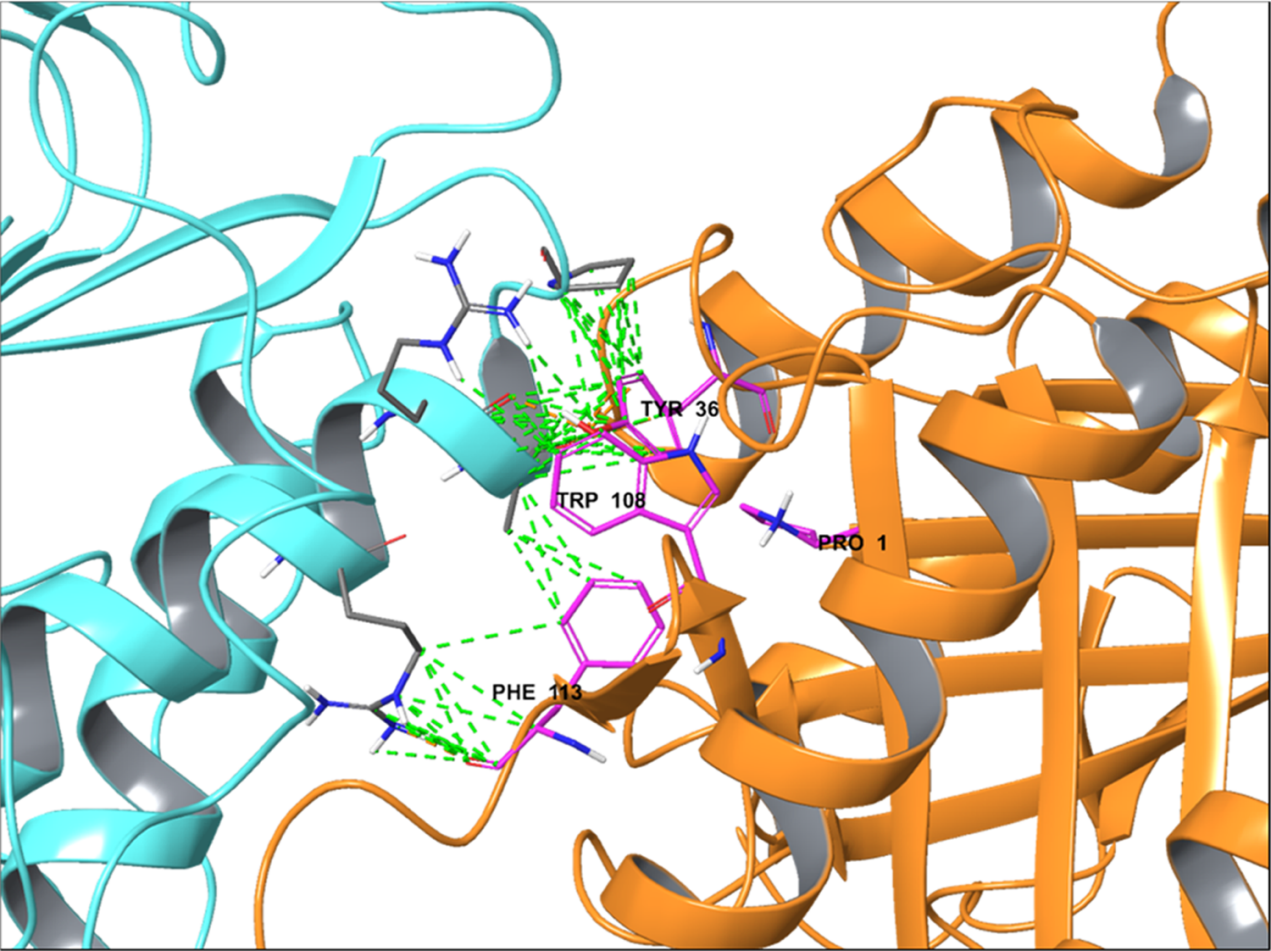
Simulated protein–protein complex of AIF (cyan, PDB: 4BV6(49)) and MIF (orange, PDB: 1GD0(32)). Interacting amino acids are shown as thin tubes. Green dashed lines show interactions of protein–protein interfaces; magenta amino acids represent site #2 (Figure 1a).

To gain an understanding of the MIF/AIF interaction, we applied a protein–protein docking method. The results of the calculations are depicted in Figure 7. The smallest protein, in this case, MIF, was considered a ligand, and AIF was considered a receptor. Two prepared proteins (corrected missing sidechains, bond orders, missing protonation, etc.) were docked using the PIPER algorithm. The protocol generated 70 000 ligand orientations, from which 50 best-fitting poses were refined and analyzed (Tables S4 and S5, Figure S6). As a result, we detected a cluster of docking solutions where MIF interacts with AIF in the region of the allosteric site colored by plum (Figure 7). We assume that inhibitor **6y** can block this interaction via a steric blockade of lipophilic residues, such as Tyr36, Trp108, and Phe113. This provides the theory to explain the binding of MIF with AIF, which can be blocked by an allosteric inhibitor.

## Conclusions

MIF is involved in protein–protein and protein–DNA interactions that play key roles in parthanatos-mediated cell death. This work discovered a new class of allosteric MIF inhibitors with a 1-phenyl-1*H*-1,2,3-triazole-4-carboxamide scaffold. A MIF inhibitor **6y** was developed by removing the aromatic alcohol functionality, a key pharmacophoric element from competitive MIF tautomerase inhibitors with a 1,2,3-triazole core. Screening of a focused compound collection of 25 new triazoles provided a MIF inhibitor with an IC_50_ of 1.7 ± 0.1 μM. Enzyme kinetic analysis demonstrated a reversible allosteric binding mode with an estimated *K*_d_ of 95 μM. The allosteric binding mode creates perspectives to interfere with MIF protein–protein interactions at sites distinct from the MIF tautomerase active site.

Further, we studied if the allosteric MIF tautomerase inhibitor is able to block the MIF/AIF co-localization that has been described to be crucial for MNNG-induced cell death. We found that **6y** can rescue HeLa cells from MNNG-induced toxicity with EC_50_ around 8 μM. It is also able to hamper MIF binding to AIF and block AIF-mediated MIF nuclear translocation, which explains the prevention of MNNG-induced parthanatos. We also found that **6y** does not directly interfere with MIF/ssDNA binding, thus indicating that the protection of genomic DNA damage occurs mostly by interfering with the AIF-mediated MIF nuclear translocation. Taken together, the allosteric MIF inhibitor **6y** prevents parthanatos and interferes with the MIF/AIF co-localization. These results emphasize the importance and potential of MIF binding molecules to interfere with its interaction in the cellular context and pave the way to understanding MIF functions and its exploitation as a therapeutic target.

## Experimental Section

### Synthetic Procedures and Analytical Methods

#### General

Nuclear magnetic resonance spectra (NMR) were recorded on a Bruker Avance 500 spectrometer ^1^H NMR (500 MHz), ^13^C NMR (126 MHz). Coupling constants were reported in hertz (Hz). Chemical shifts were reported as δ and referenced to the residual proton and carbon signals of the deuterated solvent, CDCl_3_: δ = 7.26 (^1^H) and 77.05 ppm (^13^C), DMSO-*d*_6_: δ = 2.50 (^1^H) and 39.52 (^13^C), D_2_O: δ = 4.79 (^1^H) ppm. The following abbreviations were used for spin multiplicity: s = singlet, bs = broad singlet, d = doublet, t = triplet, q = quartet, quin = quintet, dd = double of doublets, ddd = double of doublet of doublets, m = multiplet. Chemical shifts for ^13^C NMR were reported in ppm relative to the solvent peak. Analytical thin-layer chromatography was performed using pre-coated silica gel 60 F_254_ plates (Merck, Darmstadt), and the spots were visualized with UV light at 254 nm or alternatively by staining with potassium permanganate or ninhydrin solutions. Column chromatography was carried out with silica gel 60 Å (0.040–0.063 mm, 230–400 mesh). Reagents were available from commercial suppliers (Sigma-Aldrich, ABCR, Acros, and Fluorochem) and used without any purification unless otherwise noted. High-resolution mass spectra (HRMS) were recorded using a QTOF Bruker Maxis Plus, mass range 200–2000*m*/*z*, spectra rate 2.00 Hz. All compounds are >95% pure by reversed-phase high-performance liquid chromatography (RP-HPLC) Shimadzu LC – 10AT; autosampler: HiP sampler G1367A, *T* = 4 °C, 10 μL injection; flow rate: 1 mL/min; column: Kinetex 5 μm EVO C18 100 Å, *T* = 30 °C; detector: SPD-20A photodiode array detector (PDA), λ = 254 nm; solvent A: water, solvent B: ACN; gradient: 10–100%, 15 min.

#### General Procedure A: Synthesis of Propiolamides **3**

Propiolic acid **1** (122 μL, 2.0 mmol) and *N,N*′-dicyclohexylcarbodiimide (413 mg, 2.0 mmol) were dissolved in dry acetonitrile (10 mL) and cooled in an ice bath for 15 min. The corresponding amine **2** (2.0 mmol) was added to the mixture and stirred at room temperature for 2 h. The resulting precipitation was removed by filtration. The solvent was removed under reduced pressure, and the residue was used without further purification for the next step.

#### General Procedure B: Synthesis of 3-(Furan-2-yl)-*N*-(prop-2-yn-yl)propenamide **6i** (**MKA044**)

3-(Furan-2-yl)propanoic acid (280 mg, 2.0 mmol) and *N,N*′-dicyclohexylcarbodiimide (413 mg, 2.0 mmol) were dissolved in (20 mL) of dry acetonitrile and cooled in an ice bath for 15 min. Propargylamine (110 mg, 2.0 mmol) was added to the respective mixture and stirred at room temperature for 2 h. The resulting precipitate was removed by filtration. The filtrate was evaporated to dryness under reduced pressure, and the product was used without further purification.

#### General Procedure C: Synthesis of Azides **5**

Concentrated HBF_4_ (2 mL) was added dropwise to a solution of corresponding aniline **4** (5.0 mmol) in water (5 mL) over 5 min. After cooling the resulting solution to 0 °C, NaNO_2_ (350 mg, 10.0 mmol) was added portion-wise. The mixture was left stirring for 1 h at room temperature. A freshly made solution of NaN_3_ (390 mg, 6.0 mmol) in 3 mL of demi-water was added dropwise to the reaction mixture and left stirring for 1 h at room temperature. The reaction mixture was extracted with diethyl ether (3 × 20 mL). The combined organic layers were washed with brine, dried with MgSO_4_, filtered through silica gel, and concentrated under reduced pressure. Product **5** was used without further purification.

#### General Procedure D: Synthesis of Triazoles **6a-y**

Triazoles were synthesized by coupling propiolic acid to the corresponding amines, followed by CuAAC reaction with azide. Toward this aim, amide, obtained using general procedure A or B (1.0 mmol), and the corresponding azide, obtained using general procedure C (1.0 mmol), were dissolved in MeOH (5 mL) each and added into a round-bottom flask equipped with a stirring bar. Freshly prepared solutions of CuSO_4_·5H_2_O (25 mg, 0.1 mmol) and sodium ascorbate (40 mg, 0.2 mmol) in 0.5 mL of demi-water were added to the reaction mixture and left stirring overnight at room temperature. The volatiles was evaporated, and the residue was diluted with 30 mL of dichloromethane. A minimal amount of silica was added, the mixture was then evaporated under pressure, and the product was purified using medium-pressure liquid chromatography (MPLC) on silica gel. The eluent gradient used was DCM-MeOH 0-5%.

***N*****,1-Diphenyl-1*****H*****-1,2,3-triazole-4-carboxamide 6a (MKA105)** was synthesized by following general procedure D to afford **6a** (74 mg, 28%) as a white solid. ^1^H NMR (500 MHz, DMSO-*d*_6_) δ 10.6 (s, 1H), 9.4 (s, 1H), 8.0–8.0 (m, 2H), 7.9–7.9 (m, 2H), 7.7–7.6 (m, 2H), 7.6–7.5 (m, 1H), 7.4–7.4 (m, 2H), 7.2–7.1 (m, 1H) ppm. ^13^C NMR (126 MHz, DMSO-*d*_6_) δ 158.1, 143.8, 138.5, 136.3, 130.0, 129.3, 128.6, 125.6, 123.9, 120.6, 120.4 ppm. HRMS (ESI), *m*/*z* calcd for C_15_H_13_N_4_O [M + H]^+^ 265.1084, found 265.1083. HPLC: retention time 11.47 min, purity 99.5%.

***N*****-(4-Fluorophenyl)-1-phenyl-1*****H*****-1,2,3-triazole-4-carboxamide 6b (MKA103)** was synthesized by following general procedure D to afford **6b** (48 mg, 17%) as a white solid. ^1^H NMR (500 MHz, DMSO-*d*_6_) δ 10.7 (s, 1H), 9.4 (s, 1H), 8.0–8.0 (m, 2H), 7.9–7.9 (m, 2H), 7.7–7.6 (m, 2H), 7.6–7.5 (m, 1H), 7.2–7.2 (m, 2H) ppm. ^13^C NMR (126 MHz, DMSO-*d*_6_) δ 158.4 (d, *J* = 240.6 Hz), 158.0, 143.7, 136.2, 134.9 (d, *J* = 2.6 Hz), 130.0, 129.3, 125.6, 122.3, 120.5 (d, *J* = 9.3 Hz), 115.2 (d, *J* = 22.1 Hz) ppm. HRMS (ESI), *m*/*z* calcd for C_15_H_12_FN_4_O [M + H]^+^ 283.0990, found 283.0990. HPLC: retention time 11.69 min, purity 97.9%.

***N*****-(4-Bromophenyl)-1-phenyl-1H-1,2,3-triazole-4-carboxamide 6c (MKA109)** was synthesized by following general procedure D to afford **6c** (75 mg, 22%) as a white solid. ^1^H NMR (500 MHz, DMSO-*d*_6_) δ 10.8 (s, 1H), 9.5 (s, 1H), 8.0–8.0 (m, 2H), 7.9–7.8 (m, 2H), 7.7–7.6 (m, 2H), 7.6–7.5 (m, 3H) ppm. ^13^C NMR (126 MHz, DMSO-*d*_6_) δ 158.2, 143.6, 137.9, 136.2, 131.5, 129.9, 129.6, 125.7, 122.4, 120.6, 115.7 ppm. HRMS (ESI), *m*/*z* calcd for C_15_H_12_BrN_4_O [M + H]^+^ 343.0189, found 343.0190. HPLC: retention time 13.00 min, purity 99.1%.

***N*****-(4-Chlorophenyl)-1-(2-methoxyphenyl)-1*****H*****-1,2,3-triazole-4-carboxamide 6d (MKA108)** was synthesized by following general procedure D to afford **6d** (223 mg, 68%) as a white solid. ^1^H NMR (500 MHz, DMSO-*d*_6_) δ 10.7 (s, 1H), 9.0 (s, 1H), 7.9 (d, *J* = 8.9 Hz, 2H), 7.7 (dd, *J* = 7.8, 1.7 Hz, 1H), 7.6 (ddd, *J* = 9.0, 7.5, 1.7 Hz, 1H), 7.4–7.4 (m, 2H), 7.4 (dd, *J* = 8.5, 1.2 Hz, 1H), 7.2 (td, *J* = 7.7, 1.2 Hz, 1H), 3.9 (s, 3H) ppm. ^13^C NMR (126 MHz, DMSO-*d*_6_) δ 158.3, 151.9, 142.5, 137.5, 131.4, 129.3, 128.6, 127.5, 126.1, 125.2, 122.1, 120.9, 113.0, 56.3 ppm. HRMS (ESI), *m*/*z* calcd for C_16_H_14_ClN_4_O_2_ [M + H]^+^ 329.0795, found 329.0796. HPLC: retention time 12.88 min, purity 99.0%.

***N*****-(1-(4-Bromophenyl)ethyl)-1-(2-methoxyphenyl)-1*****H*****-1,2,3-triazole-4-carboxamide 6e (MKA125)** was synthesized by following general procedure D to afford **6e** (168 mg, 42%) as a white solid. ^1^H NMR (500 MHz, DMSO-*d*_6_) δ 9.1 (d, *J* = 8.2 Hz, 1H), 8.8 (s, 1H), 7.7 (dd*, J* = 7.8, 1.7 Hz, 1H), 7.6–7.5 (m, 3H), 7.4–7.4 (m, 2H), 7.4–7.3 (m, 1H), 7.2 (td, *J* = 7.5, 0.9 Hz, 1H), 5.3–5.1 (m, 1H), 3.9 (s, 3H), 1.5 (d, *J* = 7.1 Hz, 3H) ppm. ^13^C NMR (126 MHz, DMSO-*d*_6_) δ 158.8, 151.8, 144.1, 142.5, 131.1, 128.5, 128.4, 126.1, 126.0, 125.2, 120.8, 119.7, 113.0, 56.2, 47.5, 21.7 ppm. HRMS (ESI), *m*/*z* calcd for C_18_H_18_BrN_4_O_2_ [M + H]^+^ 401.0608, found 401.0608. HPLC: retention time 12.88 min, purity 98.5%.

**1-(4-Ethylphenyl)-*****N*****-(3-fluorophenyl)-1*****H*****-1,2,3-triazole-4-carboxamide 6f (MKA102)** was synthesized by following general procedure D to afford **6f** (225 mg, 73%) as a white solid. ^1^H NMR (500 MHz, DMSO-*d*_6_) δ 10.8 (s, 1H), 9.4 (s, 1H), 7.9 (d, *J* = 8.5 Hz, 2H), 7.8 (dt, *J* = 11.8, 2.3 Hz, 1H), 7.7–7.7 (m, 1H), 7.5 (d, *J* = 8.6 Hz, 2H), 7.4 (td, *J* = 8.2, 6.8 Hz, 1H), 7.0–6.9 (m, 1H), 2.7 (q, *J* = 7.6 Hz, 2H), 1.2 (t, *J* = 7.6 Hz, 3H) ppm. ^13^C NMR (126 MHz, DMSO-*d*_6_) δ 162.0 (d, *J* = 241.1 Hz), 158.4, 145.2, 143.4, 140.3 (d, *J =* 11.0 Hz), 134.1, 130.2, 129.1 (d, *J* = 10.4 Hz), 125.7, 120.5 (d, *J* = 15.7 Hz), 116.2 (d, *J* = 18.2 Hz), 107.2 (d, *J* = 19.6 Hz), 27.7, 15.5 ppm. HRMS (ESI), *m*/*z* calcd for C_17_H_16_FN_4_O [M + H]^+^ 311.1303, found 311.1303. HPLC: retention time 13.73 min, purity 98.1%.

**1-(3,4-Dimethylphenyl)-*****N*****-(4-fluorophenyl)-1*****H*****-1,2,3-triazole-4-carboxamide 6g (MKA095)** was synthesized by following general procedure D to afford **6g** (196 mg, 63%) as a white solid. ^1^H NMR (500 MHz, DMSO-*d*_6_) δ 10.6 (s, 1H), 9.3 (s, 1H), 7.9–7.9 (m, 2H), 7.8 (d, *J* = 2.4 Hz, 1H), 7.7 (dd*, J* = 8.2, 2.4 Hz, 1H), 7.4 (d, *J* = 8.2 Hz, 1H), 7.2 (t, *J* = 8.9 Hz, 2H), 2.3 (s, 3H), 2.3 (s, 3H) ppm. ^13^C NMR (126 MHz, DMSO-*d*_6_) δ 158.4 (d, *J* = 240.4 Hz), 158.1, 143.5, 138.2, 137.7, 134.9 (d, *J* = 2.7 Hz), 134.1, 130.6, 125.3, 122.3 (d, *J* = 8.4 Hz), 121.3, 117.8, 115.2 (d, *J* = 22.1 Hz), 19.4, 19.0 ppm. HRMS (ESI), *m*/*z* calcd for C_17_H_16_FN_4_O [M + H]^+^ 311.1303, found 311.1302. HPLC: retention time 13.27 min, purity 99.2%.

***N*****-(2-Chlorophenethyl)-1-(3,4-dichlorophenyl)-1*****H*****-1,2,3-triazole-4-carboxamide 6h (MKA030)** was synthesized by following general procedure D to afford **6h** (114 mg, 29%) as a white solid. ^1^H NMR (500 MHz, DMSO-*d*_6_) δ 9.36 (s, 1H), 8.84 (t, *J* = 5.8 Hz, 1H), 8.36 (d, *J* = 2.5 Hz, 1H), 8.03 (dd, *J* = 8.8, 2.6 Hz, 1H), 7.91 (d, *J* = 8.7 Hz, 1H), 7.44 (dd, *J* = 7.4, 1.8 Hz, 1H), 7.37 (dd, *J* = 7.2, 2.1 Hz, 1H), 7.31–7.23 (m, 2H), 3.57 (q, *J* = 6.8 Hz, 2H), 3.02 (t, *J* = 7.2 Hz, 2H) ppm. ^13^C NMR (126 MHz, DMSO-*d*_6_) δ 159.1, 143.9, 136.8, 135.8, 133.2, 132.4, 131.7, 131.5, 131.0, 129.2, 128.2, 127.2, 122.3, 122.2, 120.5, 38.3, 32.8 ppm. HRMS (ESI), *m*/*z* calcd for C_17_H_14_Cl_3_N_4_O [M + H]^+^ 395.0228, found 395.0229. HPLC: retention time 14.21 min, purity 97.6%.

***N*****-((1-(3,4-Dichlorophenyl)-1*****H*****-1,2,3-triazol-4-yl)methyl)-3-(furan-2-yl)-propenamide 6i (MKA044)** was synthesized by following general procedure D to afford **6i** (91 mg, 25%) as a white solid. ^1^H NMR (500 MHz, DMSO-*d*_6_) δ 8.7 (s, 1H), 8.5 (t, *J =* 5.7 Hz, 1H), 8.3 (dd, *J* = 2.5, 1.7 Hz, 1H), 8.0 (ddd, *J =* 8.8, 2.5, 1.1 Hz, 1H), 7.9 (dd, *J* = 8.8, 2.5 Hz, 1H), 7.5 (d, *J* = 1.7 Hz, 1H), 6.3 (dd, *J =* 3.2, 1.8 Hz, 1H), 6.07 (d, *J* = 3.2 Hz, 1H), 4.4 (d, *J =* 5.7 Hz, 2H), 2.9 (t, *J =* 7.7 Hz, 2H), 2.5 (t, *J* = 7.7 Hz, 2H) ppm. ^13^C NMR (126 MHz, DMSO-*d*_6_) δ 171.0, 154.7, 146.6, 141.5, 136.2, 132.4, 132.0, 130.9, 121.6, 121.3, 119.9, 110.3, 105.2, 34.1, 33.3, 23.4 ppm. HRMS (ESI), *m*/*z* calcd for C_16_H_15_Cl_2_N_4_O_2_ [M + H]^+^ 365.0567, found 365.0565. HPLC: retention time 11.22 min, purity 98.7%.

**Methyl-(1-(3,4-dichlorophenyl)-1*****H*****-1,2,3-triazole-4-carbonyl)phenylalaninate 6j (MKA084)** was synthesized by following general procedure D to afford **6j** (163 mg, 39%) as a white solid. ^1^H NMR (500 MHz, DMSO-*d*_6_) δ 9.4 (s, 1H), 9.0 (d, *J* = 8.1 Hz, 1H), 8.3 (d*, J* = 2.5 Hz, 1H), 8.0 (dd, *J* = 8.8, 2.5 Hz, 1H), 7.9 (d, *J* = 8.8 Hz, 1H), 7.3–7.2 (m, 4H), 7.2–7.2 (m, 1H), 4.8 (td, *J =* 8.2, 6.7 Hz, 1H), 3.7 (s, 3H), 3.2 (s, 1H), 3.2 (s, 1H) ppm. ^13^C NMR (126 MHz, DMSO-*d*_6_) δ 172.1, 159.7, 143.6, 138.0, 136.2, 132.8, 132.1, 129.5, 128.7, 127.0, 126.2, 122.9, 122.9, 121.1, 53.8, 36.5 ppm. HRMS (ESI), *m*/*z* calcd for C_19_H_17_Cl_2_N_4_O_3_ [M + H]^+^ 419.0672, found 419.0673. HPLC: retention time 13.64 min, purity 98.4%.

***N*****-Cyclopentyl-1-(3,4-dichlorophenyl)-1*****H*****-1,2,3-triazole-4-carboxamide 6k (MKA085)** was synthesized by following general procedure D to afford **6k** (117 mg, 36%) as a white solid. ^1^H NMR (500 MHz, DMSO-*d*_6_) δ 9.4 (s, 1H), 8.5 (d, *J* = 7.8 Hz, 1H), 8.3 (d, *J* = 2.5 Hz, 1H), 8.0 (dd, *J* = 8.8, 2.5 Hz, 1H), 7.9 (d, *J* = 8.8 Hz, 1H), 4.3–4.2 (m, 1H), 1.9–1.8 (m, 2H), 1.8–1.7 (m, 2H), 1.6–1.5 (m, 4H) ppm. ^13^C NMR (126 MHz, DMSO-*d*_6_) δ 158.7, 144.1, 135.8, 132.4, 131.5, 125.1, 122.3, 122.2, 120.5, 50.4, 32.0, 23.6 ppm. HRMS (ESI), *m*/*z* calcd for C_14_H_15_Cl_2_N_4_O [M + H]^+^ 325,0617, found 325,0617. HPLC: retention time 13.05 min, purity 99.7%.

**1-(3,4-Dichlorophenyl)-*****N*****-(furan-2-ylmethyl)-1*****H*****-1,2,3-triazole-4-carboxamide 6l (MKA038)** was synthesized by following general procedure D to afford **6l** (64 mg, 19%) as a white solid. ^1^H NMR (500 MHz, DMSO-*d*_6_) δ 9.4 (s, 1H), 9.2 (t, *J* = 6.0 Hz, 1H), 8.4 (d, *J* = 2.5 Hz, 1H), 8.0 (dd, *J* = 8.8, 2.6 Hz, 1H), 7.9 (d, *J* = 8.7 Hz, 1H), 7.6–7.6 (m, 1H), 6.4 (dd, *J* = 3.3, 1.9 Hz, 1H), 6.3 (d, *J* = 3.2 Hz, 1H), 4.5 (d, *J* = 6.0 Hz, 2H) ppm. ^13^C NMR (126 MHz, DMSO-*d*_6_) δ 159.1, 152.2, 143.6, 142.0, 135.8, 132.4, 131.8, 131.6, 125.4, 122.3, 120.6, 110.5, 106.9, 35.5 ppm. HRMS (ESI), *m*/*z* calcd for C_14_H_11_Cl_2_N_4_O_2_ [M + H]^+^ 337.0259, found 337.0254. HPLC: retention time 12.18 min, purity 98.0%.

**1-(3,4-Dichlorophenyl)-*****N*****-(3,4-dimethoxybenzyl)-1*****H*****-1,2,3-triazole-4-carboxamide 6m (MKA029)** was synthesized by following general procedure D to afford **6m** (122 mg, 30%) as a white solid. ^1^H NMR (500 MHz, DMSO-*d*_*6*_) δ 9.38 (s, 1H), 9.15 (t, *J* = 6.3 Hz, 1H), 8.34 (d, *J* = 2.5 Hz, 1H), 8.02 (dd, *J* = 8.7, 2.5 Hz, 1H), 7.89 (d, *J* = 8.8 Hz, 1H), 6.99 (d, *J* = 1.9 Hz, 1H), 6.90–6.85 (m, 2H), 4.42 (d, *J* = 6.2 Hz, 2H), 3.73 (s, 3H), 3.72 (s, 3H) ppm. ^13^C NMR (126 MHz, DMSO-*d*_*6*_) δ 159.1, 148.6, 147.8, 143.9, 135.8, 132.4, 131.9, 131.8, 131.7, 131.5, 125.2, 122.3, 120.5, 119.5, 111.7, 55.5, 55.4, 41.8 ppm. HRMS (ESI), *m*/*z* calcd for C_18_H_17_Cl_2_N_4_O_3_ [M + H]^+^ 407.0672, found 407.0673. HPLC: retention time 12.15 min, purity 98.1%.

**1-(4-Chloro-2-cyanophenyl)-*****N*****-(4-fluorophenyl)-1*****H*****-1,2,3-triazole-4-carboxamide 6n (MKA106)** was synthesized by following general procedure D to afford **6n** (143 mg, 42%) as a white solid. ^1^H NMR (500 MHz, DMSO-*d*_6_) δ 10.8 (s, 1H), 9.4 (s, 1H), 8.4 (d, *J* = 2.4 Hz, 1H), 8.1 (dd*, J* = 8.8, 2.4 Hz, 1H), 8.0 (d, *J* = 8.8 Hz, 1H), 7.9–7.9 (m, 2H), 7.3–7.2 (m, 2H) ppm. ^13^C NMR (126 MHz, DMSO-*d*_6_) δ 158.5 (d, *J* = 240.9 Hz), 157.7, 143.5, 136.4, 135.1, 134.8, 134.7 (d, *J* = 2.5 Hz), 134.2, 134.1, 128.8, 122.4, 115.3 (d, *J* = 22.2 Hz), 114.5, 109.2 ppm. HRMS (ESI), *m*/*z* calcd for C_16_H_10_ClFN_5_O [M + H]^+^ 342.0552, found 342.0555. HPLC: retention time 11.94 min, purity 99.2%.

***N*****-(4-Bromophenyl)-1-(4-cyanophenyl)-1*****H*****-1,2,3-triazole-4-carboxamide 6o (MKA097)** was synthesized by following general procedure D to afford **6o** (98 mg, 27%) as a white solid. ^1^H NMR (500 MHz, DMSO-*d*_*6*_) δ 10.8 (s, 1H), 9.6 (s, 1H), 8.3 (d, *J* = 8.8 Hz, 2H), 8.1 (d, *J* = 8.8 Hz, 2H), 7.9 (d, *J =* 9.0 Hz, 2H), 7.6 (d, *J* = 9.0 Hz, 2H) ppm. ^13^C NMR (126 MHz, DMSO-*d*_6_) δ 158.0, 143.9, 139.2, 137.8, 134.3, 131.4, 126.3, 122.5, 121.1, 118.0, 115.8, 111.7 ppm. HRMS (ESI), *m*/*z* calcd for C_16_H_11_BrN_5_O [M + H]^+^ 368.0141, found 368.0142. HPLC: retention time 12.53 min, purity 98.6%.

***N*****-(4-Ethylphenyl)-1-(4-fluoro-2-nitrophenyl)-1*****H*****-1,2,3-triazole-4-carboxamide 6p (MKA098)** was synthesized by following general procedure D to afford **6p** (169 mg, 48%) as a white solid. ^1^H NMR (500 MHz, DMSO*-d*_6_) δ 10.6 (s, 1H), 9.3 (s, 1H), 8.3 (dd, *J* = 8.1, 2.9 Hz, 1H), 8.1 (dd, *J* = 8.9, 4.9 Hz, 1H), 8.0 (ddd, *J* = 8.9, 7.8, 2.9 Hz, 1H), 7.8 (d, *J* = 8.5 Hz, 2H), 7.2 (d, *J* = 8.5 Hz, 2H), 2.6 (q, *J* = 7.6 Hz, 2H), 1.2 (t*, J* = 7.6 Hz, 3H) ppm. ^13^C NMR (126 MHz, DMSO*-d*_6_) δ 161.9 (d, *J* = 252.9 Hz), 157.6, 144.7 (d, *J* = 9.7 Hz), 143.5, 139.5, 136.0, 130.5 (d, *J* = 8.7 Hz), 129.2, 127.9, 125.5, 121.7, 120.7 (d, *J* = 15.0 Hz), 113.8 (d, *J* = 28.7 Hz), 27.7, 15.8 ppm. HRMS (ESI), *m*/*z* calcd for C_17_H_15_FN_5_O_3_ [M + H]^+^ 356.1153, found 356.1153. HPLC: retention time 12.74 min, purity 97.4%.

***N*****-(4-Chlorobenzyl)-1-(4-fluorophenyl)-1*****H*****-1,2,3-triazole-4-carboxamide 6q (MKA004)** was synthesized by following general procedure D to afford **6q** (79 mg, 24%) as a white solid. ^1^H NMR (500 MHz, DMSO-*d*_6_) δ 9.33 (t, *J* = 6.3 Hz, 1H), 9.29 (s, 1H), 8.04–7.99 (m, 2H), 7.48 (t, *J* = 8.8 Hz, 2H), 7.41–7.32 (m, 4H), 4.47 (d, *J* = 6.0 Hz, 2H) ppm. ^13^C NMR (126 MHz, DMSO-*d*_6_) δ 161.9 (d, *J* = 246.3 Hz), 159.5, 143.5, 138.5, 132.8, 131.3, 129.2, 128.2, 125.1, 122.9, 116.7, 41.4 ppm. HRMS (ESI), *m*/*z* calcd for C_16_H_13_ClFN_4_O [M + H]^+^ 331.0756, found 331.0756. HPLC: retention time 12.16 min, purity 99.6%.

***N*****-(4-Chlorobenzyl)-1-4-chlorophenyl-1*****H*****-1,2,3-triazole-4-carboxamide 6r (MKA010)** was synthesized by following general procedure D to afford **6r** (69 mg, 20%) as a white solid. ^1^H NMR (500 MHz, DMSO-*d*_6_) δ 9.33 (d, *J* = 4.9 Hz, 2H), 8.03–7.99 (m, 2H), 7.71–7.67 (m, 2H), 7.37 (q, *J* = 8.5 Hz, 4H), 4.47 (d, *J* = 6.2 Hz, 2H) ppm. ^13^C NMR (126 MHz, DMSO-*d*_6_) δ 159.4, 143.6, 138.5, 135.1, 133.5, 131.3, 130.0, 129.8, 129.2, 128.2, 122.2, 41.4 ppm. HRMS (ESI), *m*/*z* calcd for C_16_H_13_Cl_2_N_4_O [M + H]^+^ 347.0461, found 347.0464. HPLC: retention time 12.99 min, purity 99.8%.

**1-(3-Chlorophenyl)-*****N*****-phenethyl-1*****H*****-1,2,3-triazole-4-carboxamide 6s (MKA048)** was synthesized by following general procedure D to afford **6s** (104 mg, 32%) as a white solid. ^1^H NMR (500 MHz, DMSO-*d*_6_) δ 9.3 (s, 1H), 8.7 (t, *J* = 5.9 Hz, 1H), 8.1 (t, *J =* 2.0 Hz, 1H), 8.0–8.0 (m, 1H), 7.6 (t, *J* = 8.0 Hz, 1H), 7.6 (dt, *J* = 8.3, 1.4 Hz, 1H), 7.3–7.2 (m, 5H), 7.2–7.2 (m, 1H), 3.5 (dt, *J =* 8.0, 6.1 Hz, 2H), 2.9 (t, *J* = 7.5 Hz, 2H) ppm. ^13^C NMR (126 MHz, DMSO-*d*_6_) δ 159.1, 143.9, 139.4, 137.3, 134.2, 131.6, 128.9, 128.6, 128.3, 126.1, 124.9, 120.3, 119.1, 40.1, 35.1 ppm. HRMS (ESI), *m*/*z* calcd for C_17_H_16_ClN_4_O [M + H]^+^ 327.1007, found 327.1007. HPLC: retention time 12.63 min, purity 99.7%.

***N*****-(2-Chlorophenethyl)-1-(3-chlorophenyl)-1*****H*****-1,2,3-triazole-4-carboxamide 6t (MKA050)** was synthesized by following general procedure D to afford **6t** (126 mg, 35%) as a white solid. ^1^H NMR (500 MHz, DMSO-*d*_6_) δ 9.3 (s, 1H), 8.8 (t, *J* = 5.9 Hz, 1H), 8.1 (t, *J* = 2.0 Hz, 1H), 8.0–8.0 (m, 1H), 7.6 (t, *J* = 8.0 Hz, 1H), 7.6–7.6 (m, 1H), 7.4 (dd, *J* = 7.4, 1.8 Hz, 1H), 7.4 (dd, *J* = 7.2, 2.1 Hz, 1H), 7.3–7.2 (m, 2H), 3.6 (q, *J* = 6.9 Hz, 2H), 3.0 (t, *J* = 7.2 Hz, 2H) ppm. ^13^C NMR (126 MHz, DMSO-*d*_6_) δ 159.2, 143.8, 137.3, 136.8, 134.2, 133.2, 131.5, 131.0, 129.2, 129.0, 128.2, 127.2, 125.0, 120.2, 119.1, 38.3, 32.8 ppm. HRMS (ESI), *m*/*z* calcd for C_17_H_15_Cl_2_N_4_O [M + H]^+^ 361.0617, found 361.0616. HPLC: retention time 13.37 min, purity 98.5%.

***N*****-Phenethyl-1-(quinolin-5-yl)-1*****H*****-1,2,3-triazole-4-carboxamide 6u (MKA078)** was synthesized by following general procedure D to afford **6u** (96 mg, 28%) as a white solid. ^1^H NMR (500 MHz, DMSO-*d*_6_) δ 9.4 (s, 1H), 9.0 (dd, *J* = 4.2, 1.7 Hz, 1H), 8.8 (t, *J* = 6.0 Hz, 1H), 8.7 (d, *J* = 2.5 Hz, 1H), 8.5 (dd, *J* = 8.4, 1.7 Hz, 1H), 8.4 (dd, *J* = 9.1, 2.5 Hz, 1H), 8.2 (d, *J* = 9.1 Hz, 1H), 7.7 (dd, *J* = 8.4, 4.2 Hz, 1H), 7.3–7.2 (m, 4H), 7.2–7.2 (m, 1H), 3.5 (dt, *J* = 7.9, 6.0 Hz, 2H), 2.9 (t, *J* = 7.5 Hz, 2H) ppm. ^13^C NMR (126 MHz, DMSO-*d*_6_) δ 159.2, 151.7, 147.1, 144.0, 139.4, 136.6, 133.9, 131.0, 128.7, 128.4, 127.9, 126.1, 125.0, 122.9, 122.2, 118.8, 40.4, 35.1 ppm. HRMS (ESI), *m*/*z* calcd for C_20_H_18_N_5_O [M + H]^+^ 344.1506, found 344.1505. HPLC: retention time 10.74 min, purity 99.0%.

***N*****-Benzyl-1-(3,4-dimethylphenyl)-1*****H*****-1,2,3-triazole-4-carboxamide 6v (MKA027)** was synthesized by following general procedure D to afford **6v** (80 mg, 26%) as a white solid. ^1^H NMR (500 MHz, DMSO-*d*_6_) δ 9.21 (d, *J* = 2.4 Hz, 2H), 7.79 (d, *J* = 2.4 Hz, 1H), 7.68 (dd, *J =* 8.1, 2.4 Hz, 1H), 7.39–7.30 (m, 5H), 7.29–7.22 (m, 1H), 4.50 (d, *J* = 6.3 Hz, 2H), 2.31 (s, 3H), 2.27 (s, 3H) ppm. ^13^C NMR (126 MHz, DMSO-*d*_6_) δ 159.5, 143.5, 139.5, 138.2, 137.5, 134.2, 130.5, 128.2, 127.3, 126.7, 124.5, 121.3, 117.7, 42.0, 19.4, 19.0 ppm. HRMS (ESI), *m*/*z* calcd for C_18_H_19_N_4_O [M + H]^+^ 307.1553, found 307.1552. HRMS (ESI), *m*/*z* calcd for C_18_H_19_N_4_O [M + H]^+^ 307.1553, found 307.1552. HPLC: retention time 12.53 min, purity 98.6%.

**1-(4-(Cyclopropylcarbamoyl)phenyl)-*****N*****-(1-phenylethyl)-1*****H*****-1,2,3-triazole-4-carboxamide 6w (MKA122)** was synthesized by following general procedure D to afford **6w** (124 mg, 33%) as a white solid. ^1^H NMR (500 MHz, DMSO-*d*_6_) δ 9.4 (s, 1H), 9.1 (d, *J* = 8.4 Hz, 1H), 8.6 (d, *J* = 4.2 Hz, 1H), 8.1 (q, *J =* 8.8 Hz, 4H), 7.4 (d, *J* = 7.6 Hz, 2H), 7.3 (t, *J =* 7.6 Hz, 2H), 7.2 (t, *J* = 7.4 Hz, 1H), 5.2 (t*, J* = 7.5 Hz, 1H), 2.9 (tq, *J =* 7.8, 4.0 Hz, 1H), 1.5 (d*, J* = 7.1 Hz, 3H), 0.7 (dt, *J* = 6.9, 3.3 Hz, 2H), 0.6–0.6 (m, 2H) ppm. ^13^C NMR (126 MHz, DMSO-*d*_6_) δ 166.2, 158.5, 144.5, 143.9, 138.0, 134.5, 128.9, 128.2, 126.7, 126.2, 125.0, 119.9, 48.0, 23.2, 22.1, 5.7 ppm. HRMS (ESI), *m*/*z* calcd for C_21_H_22_N_5_O_2_ [M + H]^+^ 376.1768, found 376.1766. HPLC: retention time 10.13 min, purity 99.4%.

***N*****-Benzyl-1-(4-(phenylcarbomyl)phenyl)-1*****H*****-1,2,3-triazole-4-carboxamide 6x (MKA019)** was synthesized by following general procedure D to afford **6x** (107 mg, 27%) as a white solid. ^1^H NMR (500 MHz, DMSO-*d*_6_) δ 10.42 (s, 1H), 9.46 (s, 1H), 9.31 (t, *J* = 6.3 Hz, 1H), 8.25–8.15 (m, 4H), 7.83–7.79 (m, 2H), 7.41–7.32 (m, 6H), 7.26 (d, *J* = 6.4 Hz, 1H), 7.14 (t, *J* = 7.4 Hz, 1H), 4.52 (d, *J* = 6.2 Hz, 2H) ppm. ^13^C NMR (126 MHz, DMSO-*d*_6_) δ 164.2, 159.3, 146.8, 143.9, 139.5, 139.0, 138.3, 135.1, 129.5, 128.6, 128.2, 127.3, 126.7, 125.0, 120.4, 120.1, 42.0 ppm. HRMS (ESI), *m*/*z* calcd for C_23_H_20_N_5_O_2_ [M + H]^+^ 398.1612, found 398,1613. HPLC: retention time 11.73 min, purity 98.4%.

**1-(4-(Phenylcarbamoyl)phenyl)-*****N*****-(thiophen-2-ylmethyl)-1*****H*****-1,2,3-triazole-4-carboxamide 6y (MKA031)** was synthesized by following general procedure D to afford **6y** (109 mg, 27%) as a white solid. ^1^H NMR (500 MHz, DMSO-*d*_6_) δ 10.41 (s, 1H), 9.5 (d, *J* = 0.7 Hz, 1H), 9.4 (t, *J* = 6.2 Hz, 1H), 8.2–8.2 (m, 4H), 7.8 (d, *J* = 8.0 Hz, 2H), 7.4–7.3 (m, 3H), 7.1 (t, *J* = 7.4 Hz, 1H), 7.0 (d, *J* = 3.5 Hz, 1H), 7.0 (dd, *J* = 5.1, 3.3 Hz, 1H), 4.7 (d, *J* = 6.1 Hz, 2H) ppm. ^13^C NMR (126 MHz, DMSO-*d*_6_) δ 164.3, 159.2, 143.7, 142.3, 139.0, 138.3, 135.1, 129.5, 128.7, 126.6, 125.7, 125.1, 123.9, 120.5, 120.4, 120.0, 37.1 ppm. HRMS (ESI), *m*/*z* calcd for C_21_H_18_N_5_O_2_S [M + H]^+^ 404.1174, found 404.1174. HPLC: retention time 11.50 min, purity 97.8%.

### Enzyme Activity Study

#### Protein Expression and Purification

C-Terminal His-tagged and untagged recombinant human MIF was expressed with pET-20b(p) plasmid and *Escherichia coli* BL21 according to the literature procedure.^47^ After culturing, *Escherichia coli* cells were pelleted by centrifugation at 3700 g for 20 min. The pellet was sonicated in 50 mM sodium phosphate (NaPi) buffer, pH 7.2, 20 mM NaCl, 10% glycerol, and centrifuged (fixed angle) for 1 h at 30 kg and 4 °C. The supernatant was loaded on combined HiTrap Q HP and HiTrap SP HP ion exchange columns (Cytiva, 2 × 5 mL Q + 5 mL SP), equilibrated with 50 mM NaPi buffer (pH 7.2), 20 mM NaCl, and 10% glycerol. The flowthrough, which contained MIF, was collected, and 1.5 M pestle crushed ammonium sulfate (AS) was slowly added to the solution. After 1 h of mixing at 4 °C, the precipitation was centrifuged (fixed angle) for 10 min at 18 kg and 4 °C. The supernatant was collected and loaded onto a 5 mL phenyl sepharose high-performance column (Cytiva), calibrated with 50 mM NaPi buffer and 1.5 M AS, pH 7.8. MIF was eluted from the column with 50 mM NaPi buffer, pH 7.2, 20 mM NaCl, and 10% glycerol. The samples containing MIF were pooled and concentrated to ∼5 mL with 1 K Microsep Advance Centrifugal Devices (swing out centrifuge, 3220*g*). Subsequently, the sample was loaded onto HiLoad Superdex 75 PG 26/60 (GE Healthcare) size exclusion column and washed with 20 mM Tris, pH 7.5, 20 mM NaCl. Pierce BCA Protein Assay Kit (Thermo Fisher Scientific) was used to determine protein concentration. The resulting MIF was assessed by SDS gel electrophoresis, and no impurities were observed (>95%). The concentration of MIF was determined by BCA protein assay to be 1 mg/mL (70 μM). The purified protein was aliquoted and stored at −80 °C. HRMS (ESI), calcd for monoisotopic mass 14 657.3 Da, deconvoluted for monoisotopic mass 14 657.2 Da. Deconvolution is done with UniDec ver.5.05.02.^51^

#### MIF Tautomerase Activity Assay

Inhibition of the tautomerase activity and kinetics of MIF was measured using pyruvic acid (PP) as a substrate. A stock solution was prepared by mixing PP in 50 mM ammonium acetate buffer and adjusted to pH 6.0 using 1.0 M NaOH to provide a concentration of 20 mM. This solution was incubated overnight at 37 °C to allow equilibration of the keto and enol forms and then stored at 4 °C. For the assay, MIF stock solution (10 μL, 70 μM) was diluted in 20 mL of the boric acid buffer (435 mM H_3_BO_3_, 1 mM EDTA, pH 6.2) to provide 35 nM solution. The enzyme activity was determined by premixing 192 μL of the MIF dilution with 8 μL of the inhibitors dissolved in DMSO (1 mM). This mixture was preincubated for 10 min. Next, 50 μL of the mixture was mixed with 50 μL of 2 mM PP solution in ammonium acetate buffer. Subsequently, MIF tautomerase activity was monitored by the formation of the borate–enol complex, which was measured by the increase in UV absorbance at 300 nm. The increase in UV absorbance was measured over the first 10 min of incubation using a BioTek Synergy H1 Hybrid plate reader. MIF tautomerase activity in the presence of blank DMSO was set to 100% enzyme activity. As the negative control, the enzyme was excluded from monitoring the noncatalyzed conversion of the substrate, which did not show a change in absorbance at 300 nm. Data from the first 3 min were used to calculate the initial velocities. All of the graphs were prepared in GraphPad Prism.

#### IC_50_ Measurements

For the IC_50_ measurements, 1 mM solutions of the compounds were double-diluted 10 times in DMSO and subsequently, the MIF tautomerase activity was determined by premixing 190 μL of the MIF dilution with 10 μL of the inhibitors. This mixture was preincubated for 10 min and mixed with the 2 mM PP solution as described above.

#### Jump Dilution Assay

The solution of the enzyme (1.4 μM) was preincubated with a saturating concentration of inhibitor **6y** (25 μM) for 10 min. Then, the enzyme–inhibitor mixture was diluted 20-fold with boric acid buffer, then 50 μL of the mixture was mixed with 50 μL of 2 mM PP solution in ammonium acetate buffer, and recovery of the activity was measured over 20 min. In the control groups, no enzyme/inhibitor was added.

#### Enzyme Kinetics

The *K*_m_ of PP and *V*_max_ of the enzyme were determined at varying concentrations of PP (0–10 mM) with MIF (35 nM), and 0, 1.4 μM, 2.1 μM, or 2.8 μM **6y** (2.5% DMSO in buffer). Toward this aim, 10 μL of the inhibitors was added to 190 μL of the enzyme solution and incubated for 10 min. Then, 50 μL of these solutions was mixed with 50 μL of the solutions of the substrate with different concentrations. The reactions were measured by the increase in UV absorbance at 300 nm, and the data from the first 3 min were used to calculate the initial velocities. The negative control (no MIF) was subtracted from all of the data, and the curves were fitted using nonlinear regression, Michaelis–Menten, and linear regression, Lineweaver–Burk. *V*_max_ was defined as the maximum velocity as extrapolated by the curve fit. The *K*_m_ of PP was defined as the concentration of PP at which 50% of maximum velocity was reached. The data were analyzed using GraphPad Prism 8.

#### MST

MST experiments were performed on a Monolith NT.115 system (NanoTemper Technologies) using 100% LED and 60% IR-laser power. Laser on and off times were set at 30 and 5 s, respectively. A 100 nM dye solution RED-tris-NTA was prepared by mixing 2 μL of the dye (5 μM in DMSO) and 98 μL of PBS-T. The protein concentration was adjusted to 200 nM, and 100 μL of this solution was added to the tube with dye and incubated at rt for 30 min. Meanwhile, a twofold dilution series of compound **6y** was prepared in PBS-T containing 5% DMSO. Subsequently, 10 μL of labeled MIF was mixed with 10 μL of the samples with 16 different concentrations of **6y** ranging from 2000 to 0.06 μM. The samples were centrifuged for 10 min at rt and the supernatant was transferred into fresh tubes. After that, the standard treated capillaries (K002) were filled with the solutions and the MST curves were measured at 25 °C.

#### ELISA

Human recombinant untagged MIF was diluted in the binding buffer (Tris-HCl 100 mM, 5 mM MgCl_2_, 150 mM NaCl, pH 8.5) to a final concentration of 250 nM. 100 μL of MIF solution was used to coat each well of a high-binding 96-well plate overnight at 4 °C. The next day, after washing three times with washing buffer (TBS with 0.05% tween 20), the plate was blocked with 2% BSA in binding buffer for 2 h at room temperature. Later, the MIF-coated plate was inhibited with a series of concentrations of 6y for 30 min at rt followed by adding his-tagged recombinant AIF (final concentration 4 μM, Novus Biologicals, Centennial). After 2 h incubation, the plate was washed three times with washing buffer and incubated with anti-poly-Histidine–peroxidase antibody (Sigma, Amsterdam, the Netherlands) for 30 min. After washing, 100 μL of mixed HRP substrate reagent (R&D Systems, Minneapolis) was added, and the colorizing reaction was stopped by adding 100 μL of 1N sulfuric acid. The absorbance at 450 nM was determined with the correction at 570 nM via a Synergy H1 plate reader.

#### MIF Labeling and MIF/DNA Binding Assay

Five equivalents of probe ZP307 were mixed with recombinant MIF protein for MIF labeling and incubated overnight at 4 °C. Later, the free probe ZP307 was removed by a PD-10 desalting column. The MIF tautomerase activity assay was employed to assess the degree of labeling. The labeled MIF was kept at −20 °C for further experiments. The MIF/ssDNA binding assay was performed in the reaction buffer (100 mM Tris, 5 mM MgCl_2_, 5 mM KCl, pH 8.2). Probe **ZP307** labeled MIF (1 μM) preincubated with MIF inhibitor (100 μM) for 1 min at rt. Later, PA20-Q1 (1 μM) was added to each well. The fluorescence (Ex488/Em520) was determined immediately using a BioTek Synergy H1 Hybrid plate reader. The wells in the absence of PA20-Q1 were considered a positive control (100% of fluorescence signal), whereas the wells without inhibitors were considered a negative control (0% of fluorescence signal).

### Cell-Based Studies

#### Cell Culture

HeLa cell line was purchased from ATCC and cultured in Dulbecco’s modified Eagle’s medium (Invitrogen) containing 1% (w/v) penicillin G/streptomycin (Invitrogen) supplemented with 10% (v/v) fetal bovine serum (FBS) (Costar Europe, the Netherlands) in a humidified incubator at 37 °C with 5% CO_2_.

#### Cell Viability Assay

HeLa cells were seeded in 96-well plates at a density of 2.4 × 10^3^ cells per well. After overnight culturing, the cells were treated with MIF inhibitor **6y** at different concentrations for 3 h. After that, MNNG was added to each well to reach a final concentration of 50 μM. Plates were incubated at 37 °C for 15 min. Then, the compound-containing medium was replaced by 100 μL of fresh culture medium. The cells were incubated for another 24 h. Subsequently, 20 μL of CellTiter96 Aqueous One Solution reagent (Promega) was added to each well. The plates were incubated at 37 °C for 1.5 h. The OD490 of each well was determined by a Synergy H1 plate reader (BioTek).

#### Cell Death Determination by Flow Cytometry

HeLa cells were seeded in 6-well plates at 5 × 10^5^ cells/well density and incubated overnight. The cells were pretreated with 100 μM MIF inhibitor **6y** and 100 μM MIF tautomerase inhibitor ZP143 for 3 h. Later, 50 μM MNNG was used to treat cells for 15 min to induce parthanatos. Then, the compound-containing medium was replaced by fresh culture medium, and the cells were incubated for another 24 h. After that, the cells were harvested via trypsinization and washed with ice-cold PBS. Subsequently, the cells were stained with 7 μM Hoechst 33342 and 2 μM propidium iodide (PI), and then measured using NovoCyte Quanteon (Agilent, Santa Clara). The cells stained only with Hoechst 33342 were counted as live cells, while cells stained by both Hoechst 33342 and PI were counted as dead cells.

#### Comet Assay

Comet assays were conducted following protocols published previously.^52^ Briefly, HeLa cells were treated with or without 50 μM MIF nuclease inhibitor **6y** for 2 h, followed by stimulation with 10 μM MNNG for another 2 h. The cells were then harvested and resuspended in ice-cold PBS (divalent cations free) at 2 × 10^4^ cells/mL density. 400 μL of cell suspension was mixed with 1.2 mL of 1% (w/v) low-melting-point agarose at 40 °C. The mixture was immediately pipetted onto a pre-coated comet slide and placed flatly in a dark, cold room for 5 min for gelling. Then, slides were submerged in an alkaline lysis solution (1.2 M NaCl, 100 mM EDTA, 0.1% sodium lauryl sarcosinate, 260 mM NaOH, pH > 13) overnight at 4 °C. Gel electrophoresis was then conducted in an alkaline electrophoresis solution (30 mM NaOH, 2 mM EDTA pH > 12) at a voltage of 0.6 V/cm for 25 min. After that, slides were rinsed twice in 400 μL of dH_2_O. Later, slides were stained with 2.5 μg/mL of PI in dH_2_O for 20 min. Cell images were acquired using a Leica DM4000b fluorescence microscope and analyzed by ImageJ.

#### Immunofluorescence Staining and Confocal Microscopy

Immunofluorescence staining was applied to cells to determine the nuclear trans-localization of AIF and MIF. HeLa cells were seeded onto a coverslip and treated with or without 100 μM **6y** for 3 h. Then, parthanatos was induced by MNNG (50 μM, 15 min). The same amount of medium in the absence of MNNG was added for the negative control group. After overnight incubation, the cells were fixed with methanol and blocked with 2% BSA in 0.1% PBS-T. AIF and MIF were detected by AIF Monoclonal Antibody (7F7AB10, Invitrogen) and MIF Polyclonal Antibody (PA5-27343, Invitrogen), respectively. Then, AIF and MIF primary antibodies were visualized by Alexa Fluor 647 conjugated anti-Mouse IgG (Invitrogen) and Alexa Fluor 488 conjugated anti-rabbit IgG (Cell Signaling Technology, Leiden, the Netherlands), respectively. After washing, coverslips were mounted onto objective slides with an anti-fading mounting medium with NucBlue stain (Invitrogen). The pictures were acquired using a Leica SP8 confocal laser scanning microscope and analyzed by ImageJ.

## Supporting Information Available

^1^H NMR, ^13^C NMR, and HPLC for all final compounds; synthetic procedures; biological data; and details of computational methods (PDF)
Molecular formula strings (CSV)
MIF-AIF-protein-protein-docked_pose_1 (PDB)
MIF-AIF-protein-protein-docked_pose_2 (PDB)
MIF-AIF-protein-protein-docked_pose_3 (PDB)

## Supplementary Material

jm3c00397_si_002.csv

jm3c00397_si_003.pdb

jm3c00397_si_004.pdb

jm3c00397_si_005.pdb

## Abbreviations Used

4-HPP: 4-hydroxyphenyl pyruvate
AIF: apoptosis-inducing factor
BHQ1: black hole quencher-1
CD74: cluster of differentiation 74
CuAAC: copper(I)-catalyzed alkyne-azide cycloaddition
CXCR: C-X-C chemokine receptor
DCC: *N*,*N*′-dicyclohexylcarbodiimide
MIF: macrophage migration inhibitory factor
MNNG: *N*-methyl-*N*′-nitro-*N*-nitrosoguanidine
MST: microscale thermophoresis
MTS: 3-(4,5-dimethylthiazol-2-yl)-5-(3-carboxymethoxyphenyl)-2-(4-sulfophenyl)-2H-tetrazolium
PAAN: parthanatos-associated AIF nuclease
PAR: poly (ADP-ribose)
PARP-1: PAR polymerase 1
PP: phenyl pyruvate
RP-HPLC: reversed-phase high-performance liquid chromatography
ssDNA: single-stranded DNA
TRX: thioredoxin
