# Supplementary material for: Allosteric Inhibitors of Macrophage Migration Inhibitory Factor (MIF) Interfere with Apoptosis-Inducing Factor (AIF) Co-Localization to Prevent Parthanatos
Source: J Med Chem. 2023 Jun 23;66(13):8767–81. doi: 10.1021/acs.jmedchem.3c00397 (PMC10350922; doi:10.1021/acs.jmedchem.3c00397)
Supplement: Supplementary file 1 — jm3c00397_si_001.pdf [file jm3c00397_si_001.pdf]

Supporting information for

Allosteric inhibitors of macrophage migration inhibitory factor (MIF) interfere with apoptosis-inducing factor (AIF) co-localization to prevent parthanatos

Deng Chen‡[1], Angelina Osipyan‡[1], Jeaunice Adriana[1], Mohammed Kader[1], Maxim Gureev[2], Catharina W. J. Knol[1], Marie-Cathérine Sigmund[1], Zhangping Xiao[1], Petra E. van der Wouden[1], Robbert H. Cool[1], Gerrit J. Poelarends[1], and Frank J. Dekker*[1].

[1]*Department of Chemical and Pharmaceutical Biology, Groningen Research Institute of Pharmacy, University of Groning-en, Antonius Deusinglaan 1, 9713 AV, Groningen, The Netherlands.*

[2]*Center of Chemo- and bioinformatics, Institute of Biodesign and Complex Systems Modeling, I. M. Sechenov First Mos-cow State Medical University, 119435, Moscow, The Russian Federation*

[‡] These authors contributed equally to this work

**Corresponding author: Dekker, F.J. (f.j.dekker@rug.nl)*

Table of Contents

1. Supporting figures and tables 3
    Enzyme kinetics 3
    MST data 4
    Cell-based studies 5
    MIF/AIF protein-protein docking protocol 8
2. Copies of ^{1}H and ^{13}C NMR spectra of all compounds 10
3. Copies of HPLC data of all compounds 35
4. Reference 62

1. Supporting figures and tables

Enzyme kinetics

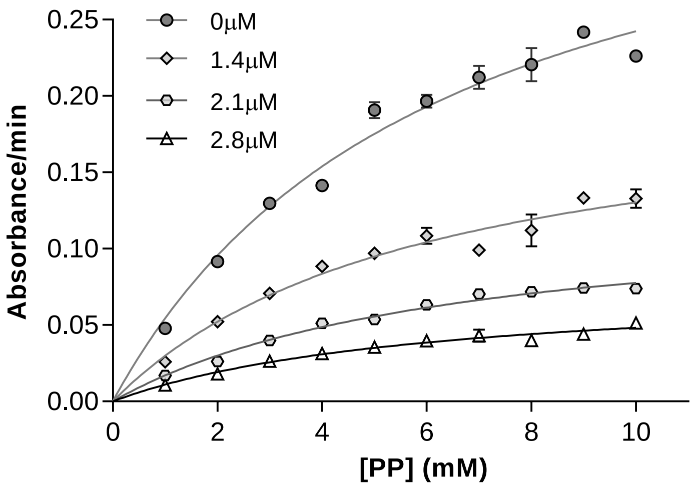

Figure S1. Michaelis-Menten plot of MIF inhibition by 6y at the concentrations 0, 1.4, 2.1 and 2.8 μM.

Table S1. Kinetic parameters of 6y measured at 35nM of MIF.

	**0 μM**	**1.4 μM**	**2.1 μM**	**2.8 μM**
**V_{max} (abs/min)**	0.39±0.03	0.21±0.02	0.13±0.01	0.08±0.01
**K_m (mM)**	6.24±0.84	5.98±1.07	6.58±0.72	6.04±0.99

1 Experiment 1

Experiment Type: Binding Affinity
Filename: C:\Users\nanotemper\Documents\NanoTemperData\Angelina\08122022 MIF MKA031.moc
Date measured: Thu, 08 Dec 2022 19:08:36 GMT

Target: 50 nM MIFhis
Ligand: 1E+03 µM MKA_031

Buffer: MST Buffer including 0.05% Tween
Capillary: Monolith NT.115 Standard Treated Capillary (K002)
Excitation Color: Red
Excitation Power: 60% (Auto-detect)
MST Power: Medium

Device: Monolith NT.115 (201703-BR-N002)

Comment:

Dose Response

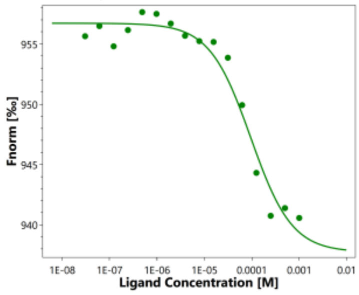

Response Evaluation: On Time 1.5s
Kd model
Unbound 956.7
Bound 937.7
Kd 95 µM
TargetConc 50 nM
Response Amplitude: 19.0
Noise: 1.2
Signal to Noise Ratio: 15.8

Capillary Scans

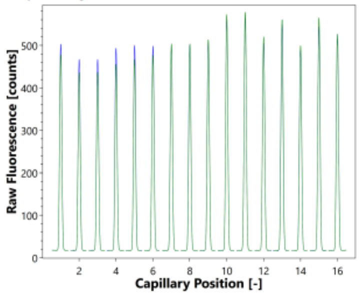

Initial Fluorescence:
Average: 506 counts
Variation: ±14.2 %
No adsorption
No Ligand Induced Fluorescence Change

MST Traces

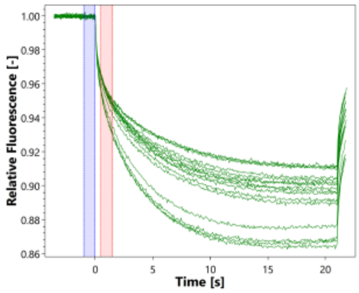

Cursor positions:
Cold Region: -1s - 0s
Hot Region: 0.5s - 1.5s
No Aggregation
No Ligand Induced Photobleaching Rate Change

MST data

Figure S2. Full report of the MST experiment. Normalized fluorescence Fnorm = Fhot/Fcold

Cell-based studies

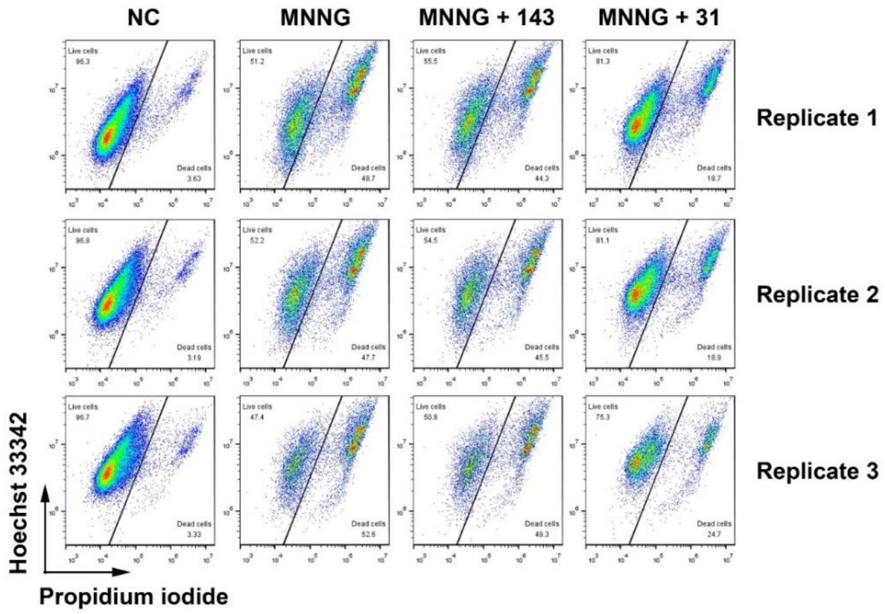

Figure S3. Replicates for Figure 5b. **6y** (**MKA031)** rescued cells from MNNG treatment. HeLa cells were seeded in a 6-well plate. MIF was pre-inhibited by **6y,** later on, HeLa cells were treated either with DMSO or MNNG (50 µM, 15 min) to induce parthanatos. After 24 h incubation, cells were harvested and dual stained with Hoechst 33342 and propidium iodide. Live and dead cells were determined by FACS.

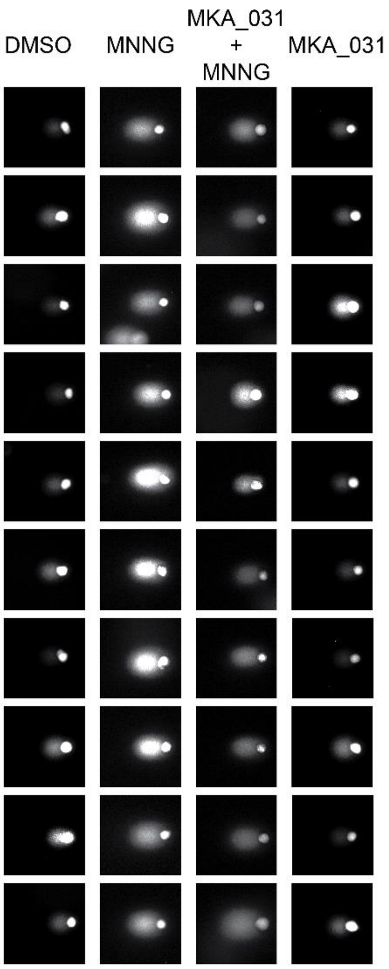

Figure S4. The source images of the comet assay shown in Figure 6a.

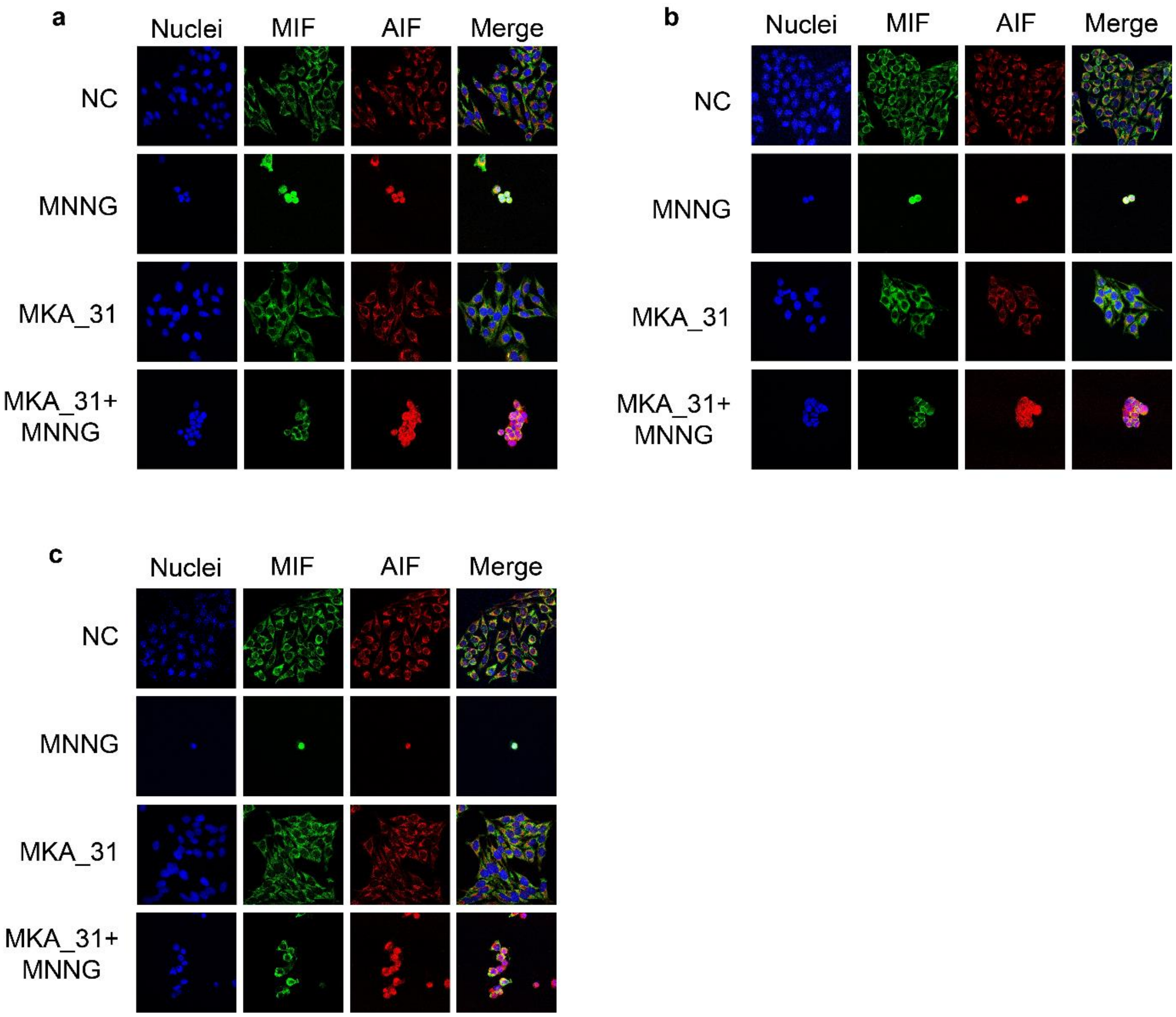

Figure S5. The uncropped images and three replicates a-c) for the Figure 6d.

Table S2. Sequence of MIF substrate

**Name**	**Sequence**
**PA20-Q1**	5’-[BHQ1]AAAAAAAAAAAAAAAAAAAA-3’

MIF/AIF protein-protein docking protocol

Table S3. Hydrophobic areas parameters considered as potential binding sites

Area#	Hydrophobic int. score	Size (Å^2)	Intensity (score/size)	Aminoacids
1	88.83	108.4	0.82	B:SER90; C:SER90; A:SER90; B:MET101; A:MET101; C:MET101; C:TYR99; B:TYR99; A:TYR99; A:PRO91; B:PRO91; C:PRO91; A:ARG93; B:ARG93
2	201.78	320.4	0.63	B:TYR36; B:PHE113; B:ASN97; B:TRP108; B:THR23; A:TYR95; B:ASN102; B:GLU54; B:MET101; B:SER53; B:ILE64; B:TYR98; B:TYR95; B:LEU26; A:HID40; B:LEU22; B:GLN25; B:PRO33; B:ILE96; B:VAL94; B:VAL106
	246.33	379.5	0.65	C:TYR36; C:PHE113; C:ALA104; C:ASN97; C:TRP108; C:THR23; B:TYR95; C:GLU54; C:LEU26; C:MET101; C:LEU115; C:ASN102; C:SER53; C:TYR98; C:ILE64; C:ASN105; C:TYR95; C:GLN25; C:PRO33; C:ILE96; C:ALA103; C:VAL94; B:HID40
	268.29	433.3	0.62	A:TYR36; A:PHE113; A:ALA104; A:ASN97; A:TRP108; A:THR23; A:GLN24; C:TYR95; A:GLU54; A:LEU26; A:ASN102; A:MET101; A:SER53; A:LEU115; A:TYR95; A:ILE64; A:ASN105; A:TYR98; A:GLN25; A:PRO33; A:ILE96; A:PRO34; C:HID40; A:ALA103; A:VAL94; A:VAL106
3	44.19	123.3	0.36	B:PRO43; B:PRO1; B:LEU79; A:HID118; B:PHE3; B:PRO10; B:PRO55
	30.66	85.3	0.36	A:PRO1; A:PRO43; A:PHE3; A:PRO10
	34.99	97.7	0.36	C:PRO1; C:PRO43; C:PHE3; B:ALA114; C:PRO10; C:PRO55
4	33.36	63.8	0.52	A:ILE37; B:VAL9; B:ILE4; A:LEU46
	33.21	66.9	0.50	B:ILE37; C:VAL9; C:ILE4; B:LEU46
	25.76	55.0	0.47	C:ILE37; A:VAL9; C:LEU46

On the basis of parameters of interaction intensity, the most preferred areas for ligand binding are 2 and 4. Also, this fact is supported by cavity geometry, which has «canyon-like» shape, favorable for linear ligands.

MIF protein trimer, PDB model 1GD0/4K9G, and AIF, PDB model 4BV6, which was considered as a receptor, were used for calculations. Protein structures were prepared and refined: all ligands, water, and other proteins (if present) were removed, missing sidechains were added, and the hydrogen bond network was optimized. Protein-protein docking was performed with use of PIPER algorythm[1,2]. During the docking, 70000 orientations were sampled in standard mode, with 50 best-fitting on output. Each of the resulting poses was refined. Repulsion/attraction restraints were not used. The best solution was discovered using solutions clustering and by energy value. The selection of effective poses is implemented by the value of the PoseScore parameter and the clustering of docking solutions. As a result, a cluster of solutions was found with parameters characterizing it as energetically favorable and stable. Resulting pose analysis showed that all amino acids involved in the protein-protein contact corresponds to zone 2 (Figure 1a and Table S3), previously singled out as one of the most promising sites for protein-ligand interactions. Key hydrophobic contacts of MIF/AIF are realized within following amino acids: Pro33, Tyr36, Asn102, Trp108, Phe113. Figure S5 depicts clustered docking solutions, and the row

coloring correlates with the ribbon coloring (these are three averaged structures with similar binding pose

Table S4. Poses energy

Pose	Cluster volume	Pose energy (kcal/mol)	Pose score (kcal/mol)
9	20	-612.04	-191.22
7	23	-543.60	-131.05
33	11	-542.63	-120.68

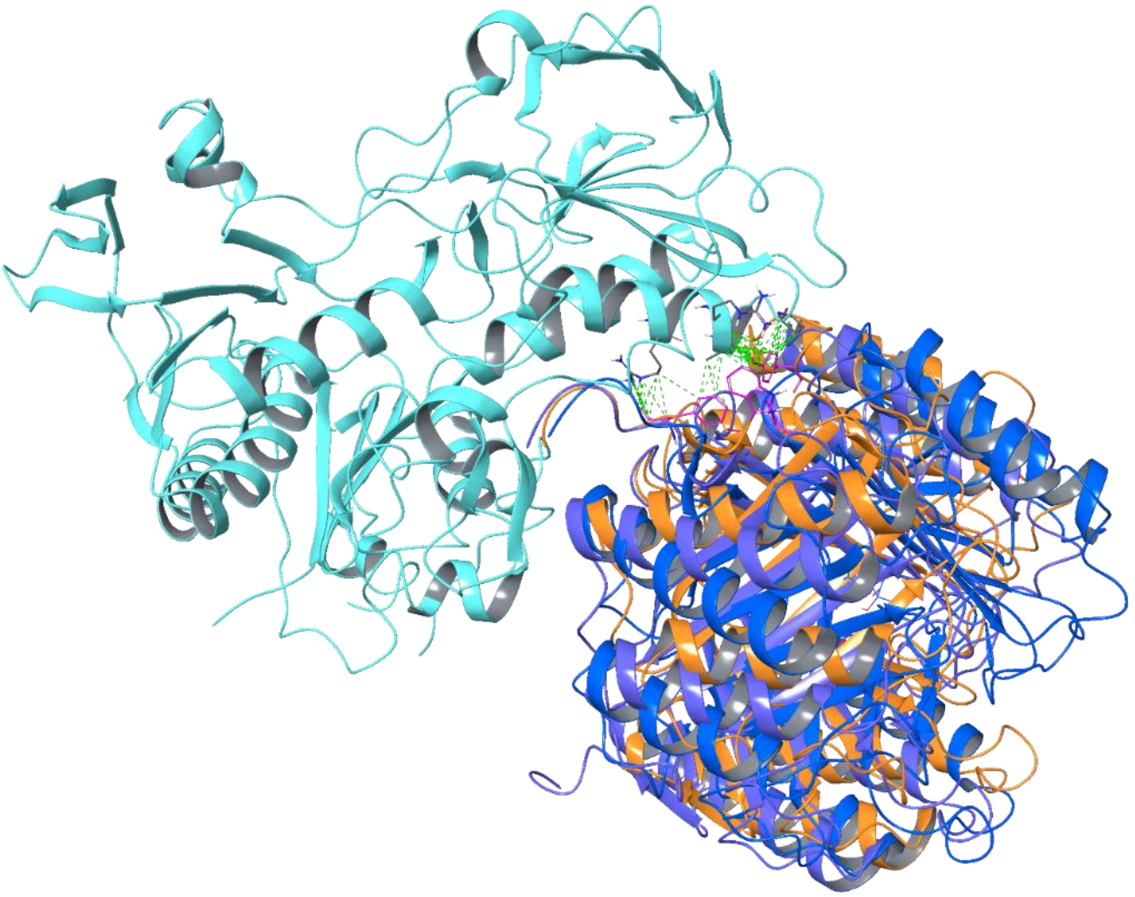

Figure S6. The clustered docking solutions for MIF/AIF interaction

Table S5. Key hydrophobic contacts of MIF/AIF

AIF	MIF
Phe144, Ala145, Arg148, Arg151-Pro155, Met171, Arg201-Phe205, His455, Val459, Arg463, Ile528, Ser530, Glu531	B: Ala70, Arg73; C: Gly31, Pro33, Gln35, Tyr36, Asn102, Trp108, Phe113

2. Copies of ^{1}H and ^{13}C NMR spectra of all compounds

^{1}H and ^{13}C NMR spectra of compound 6a (MKA105)

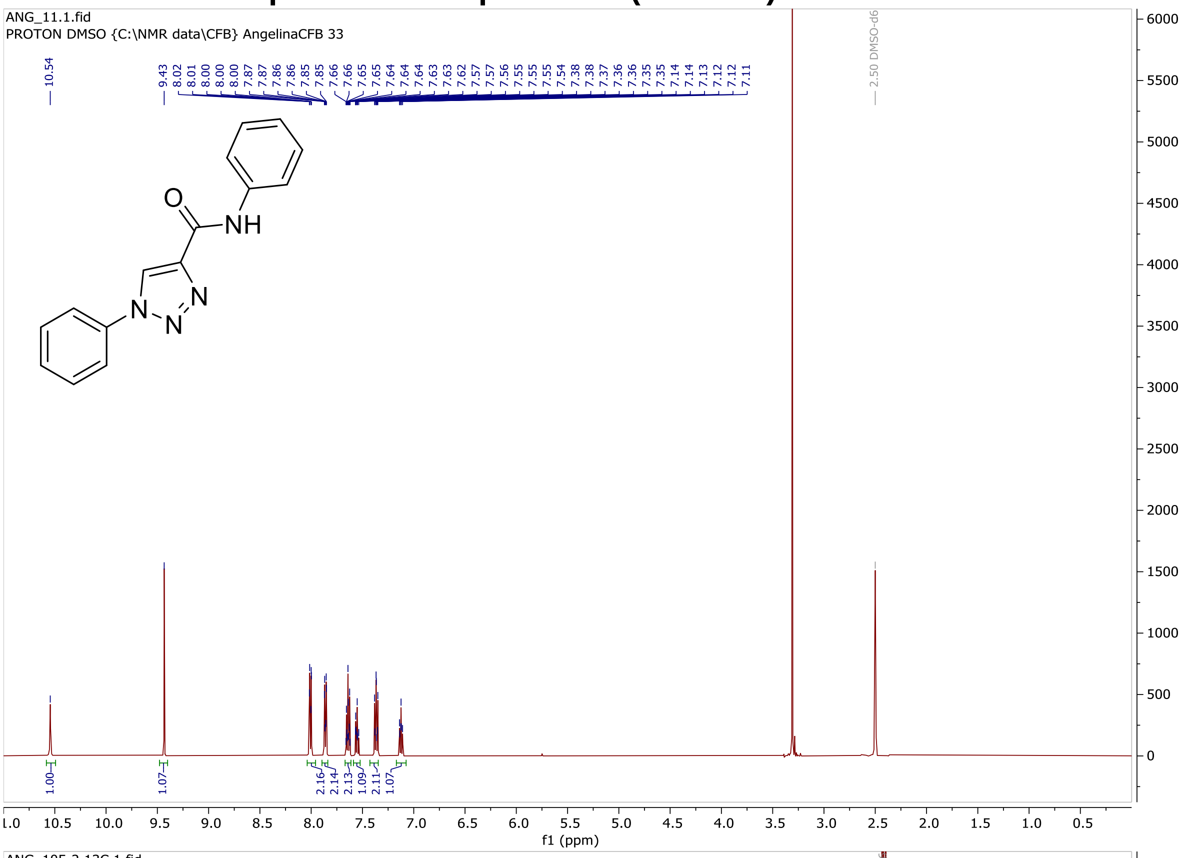

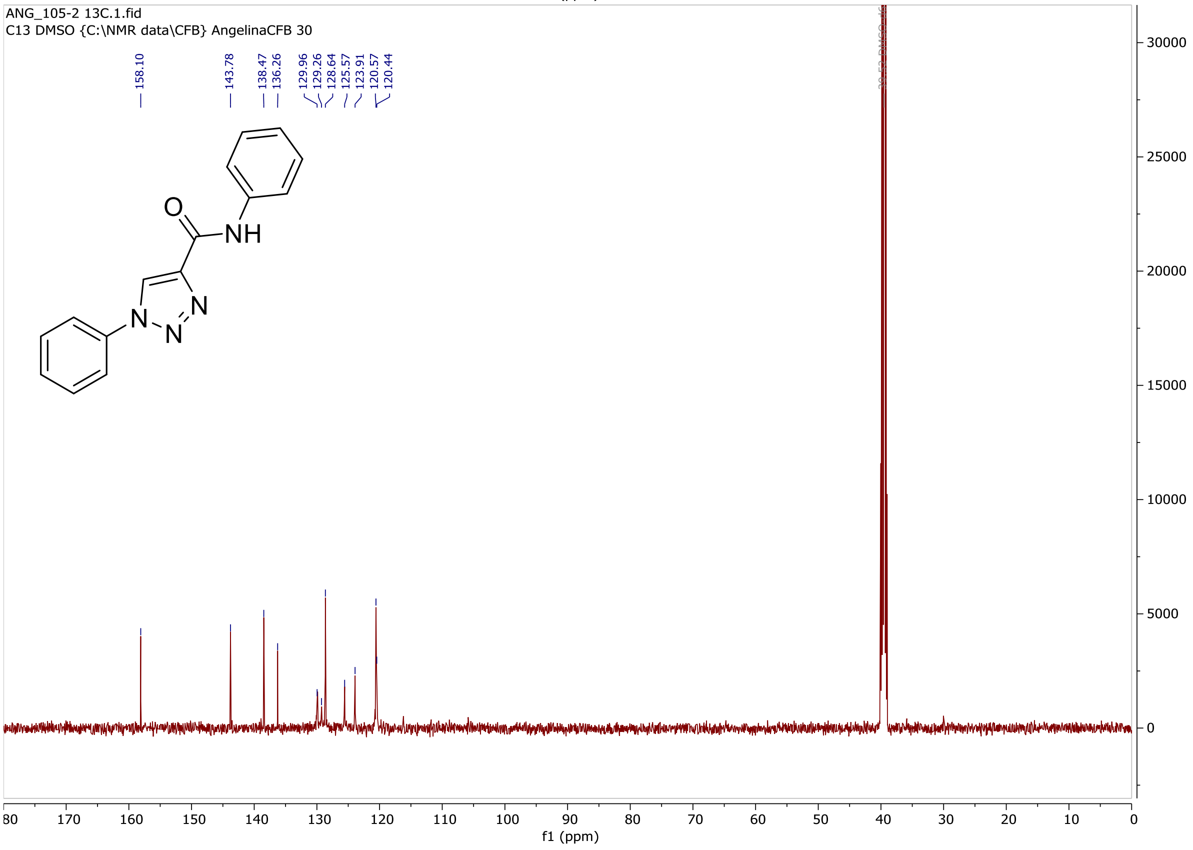

^{1}H and ^{13}C NMR spectra of compound 6b (MKA103)

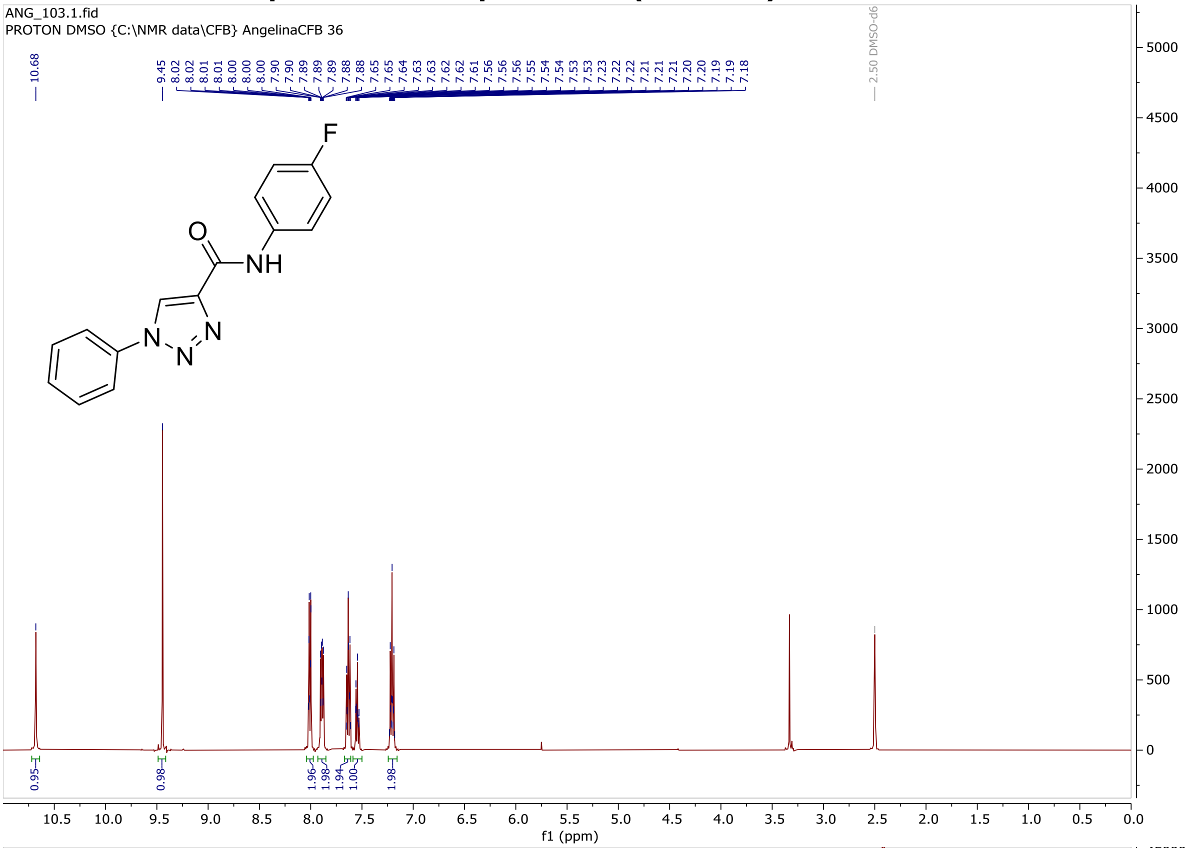

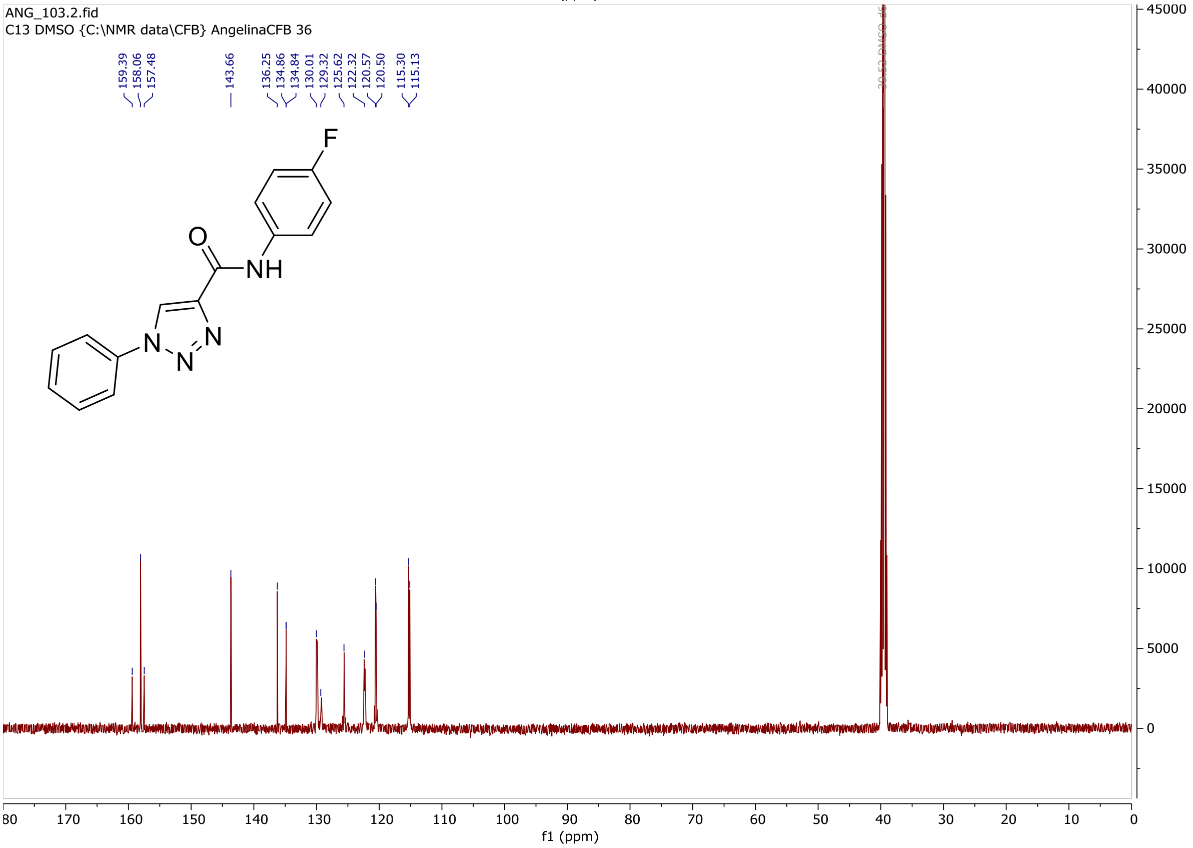

1H and 13C NMR spectra of compound 6c (MKA109)

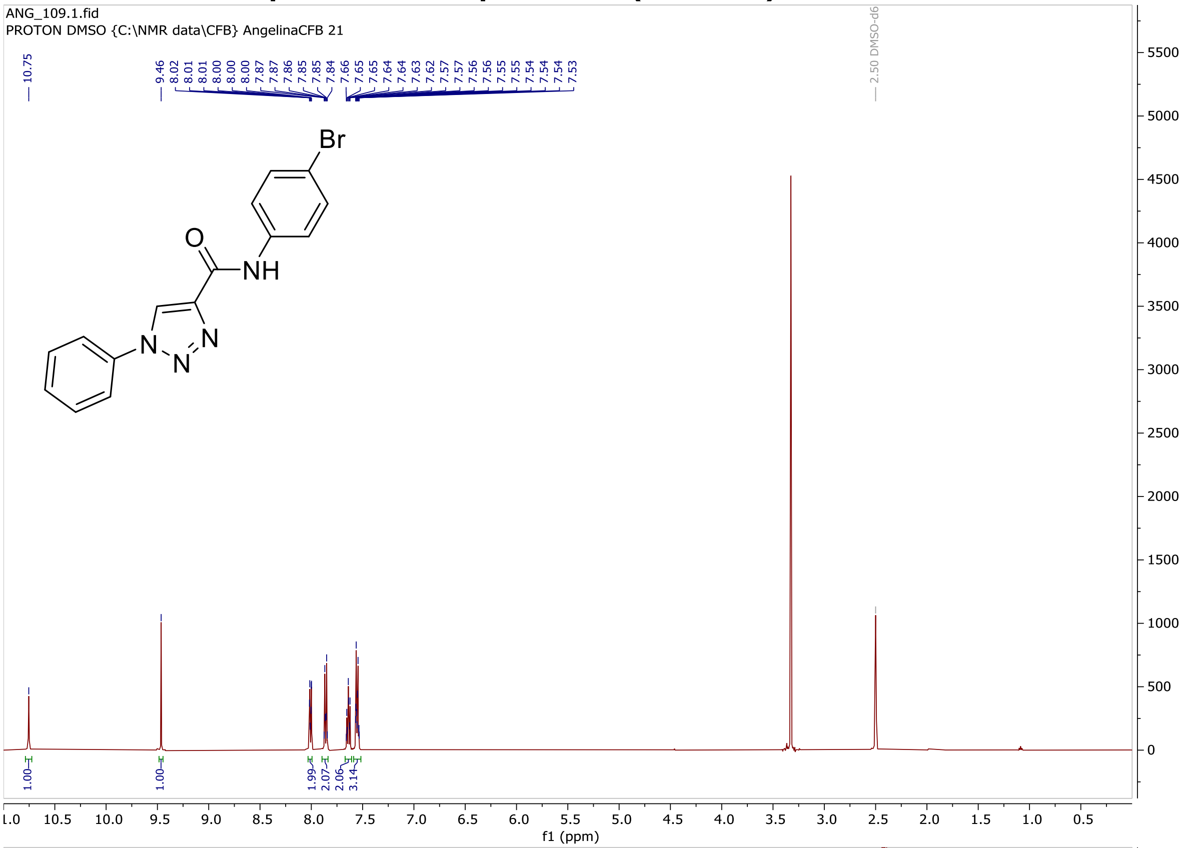

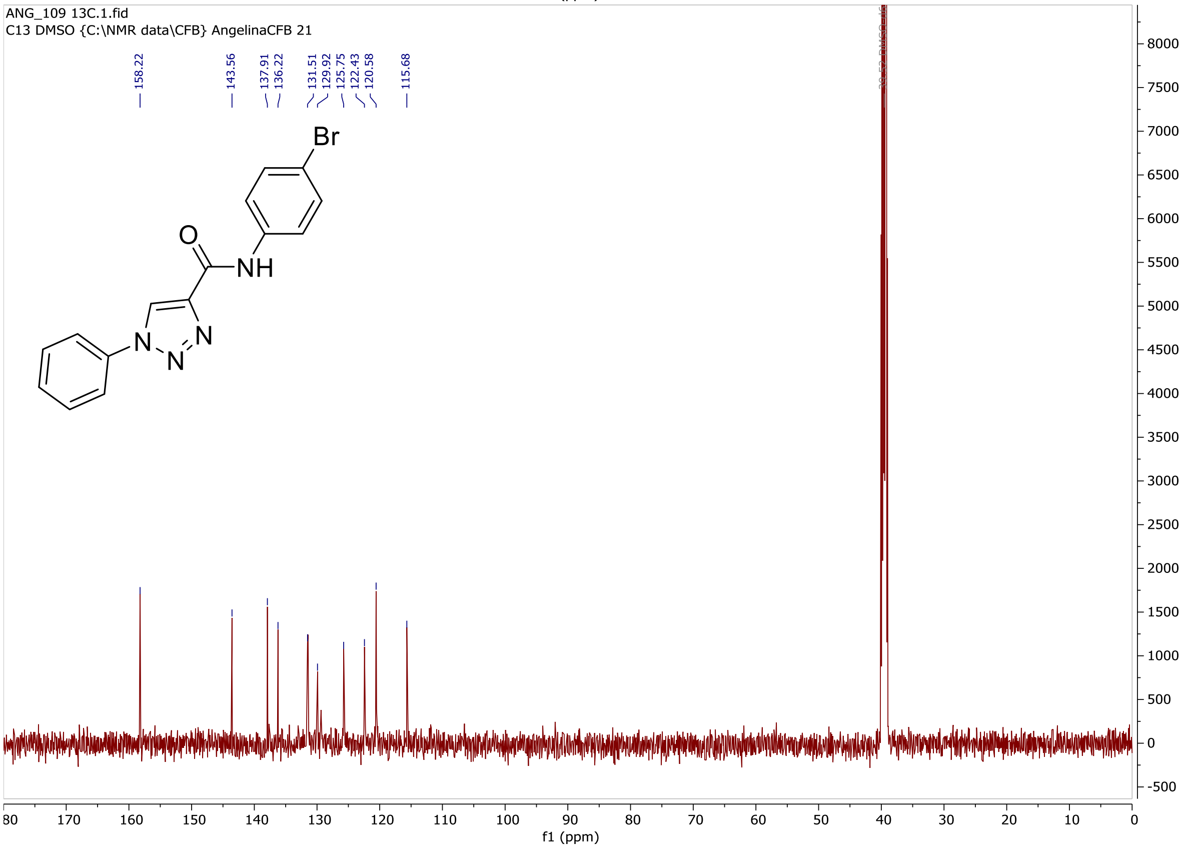

^{1}H and ^{13}C NMR spectra of compound 6d (MKA108)

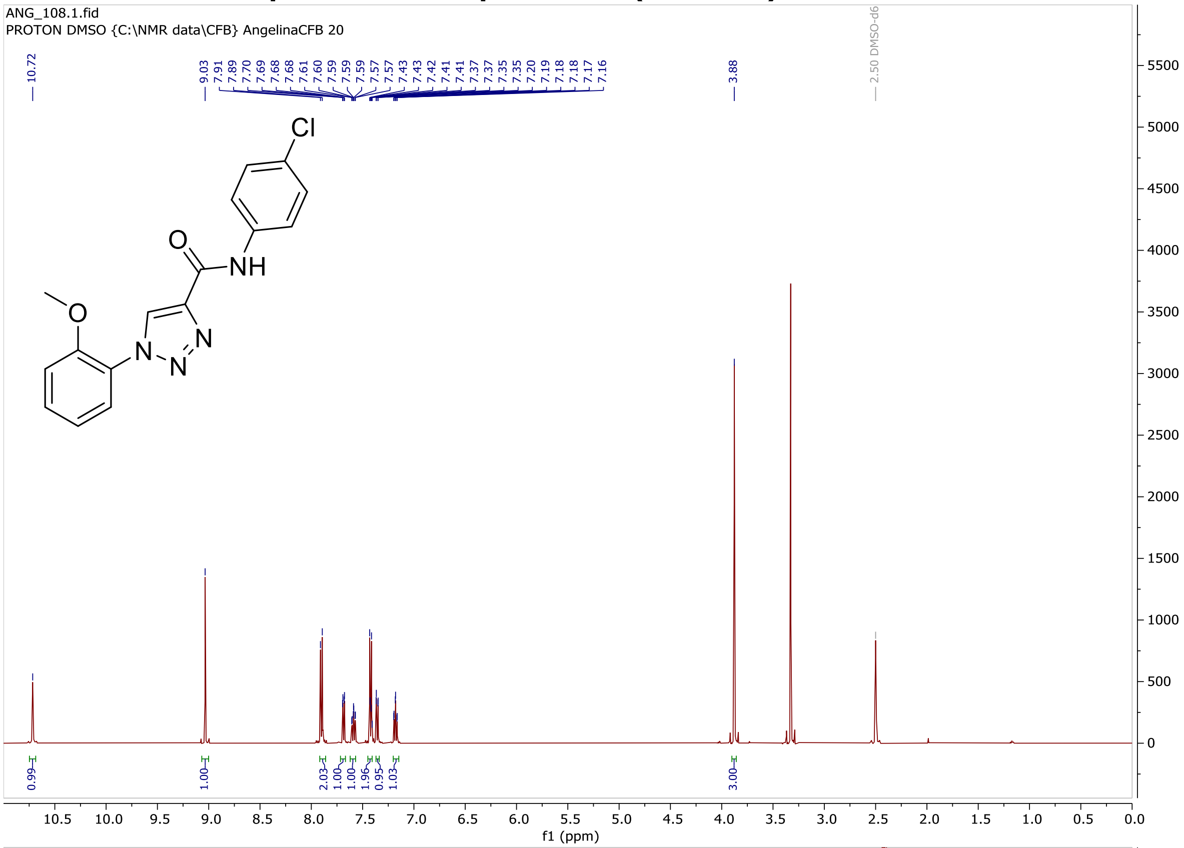

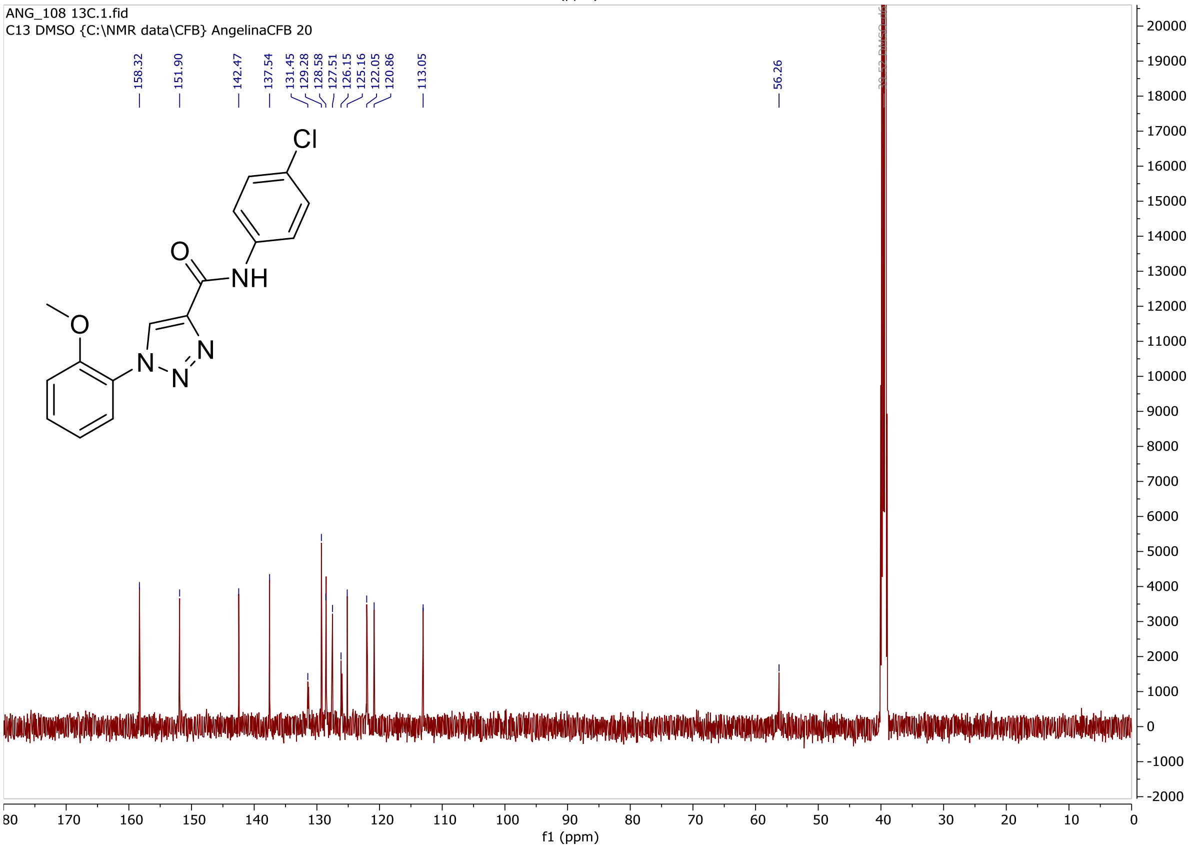

^{1}H and ^{13}C NMR spectra of compound 6e (MKA125)

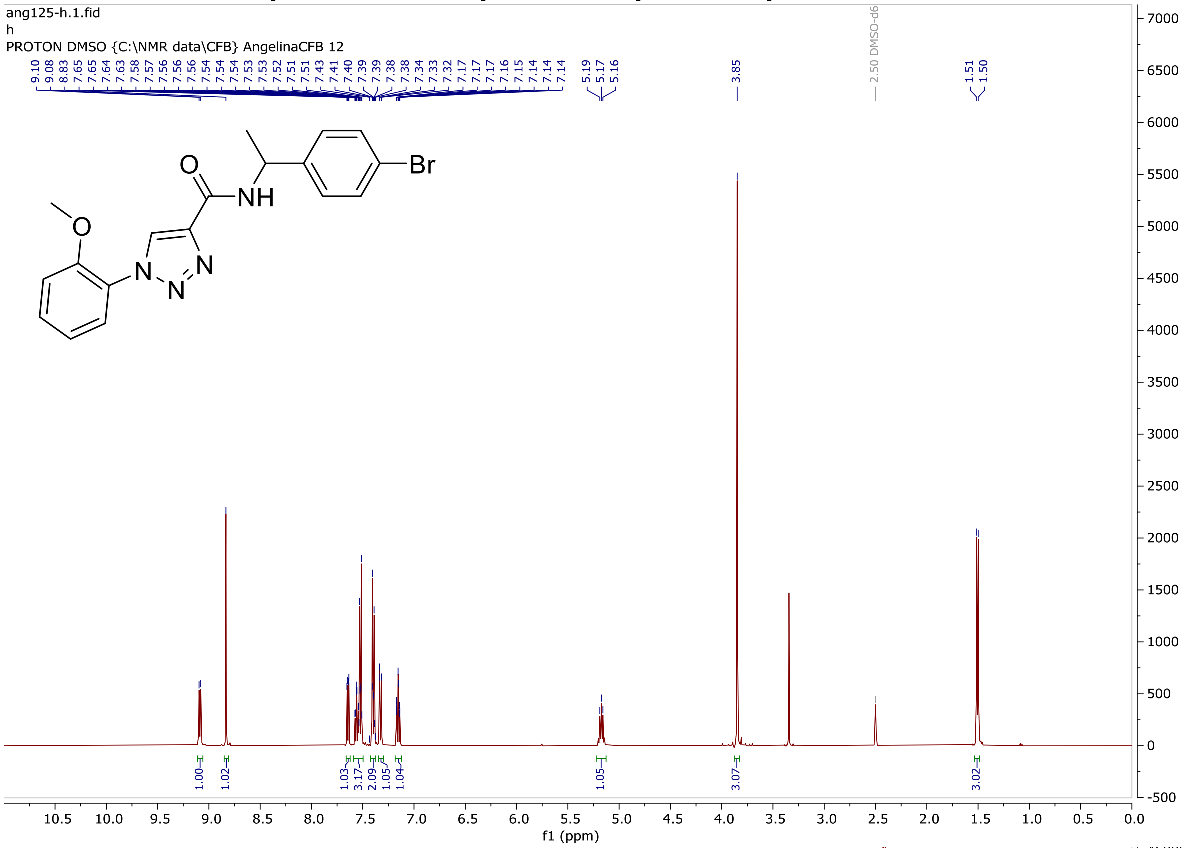

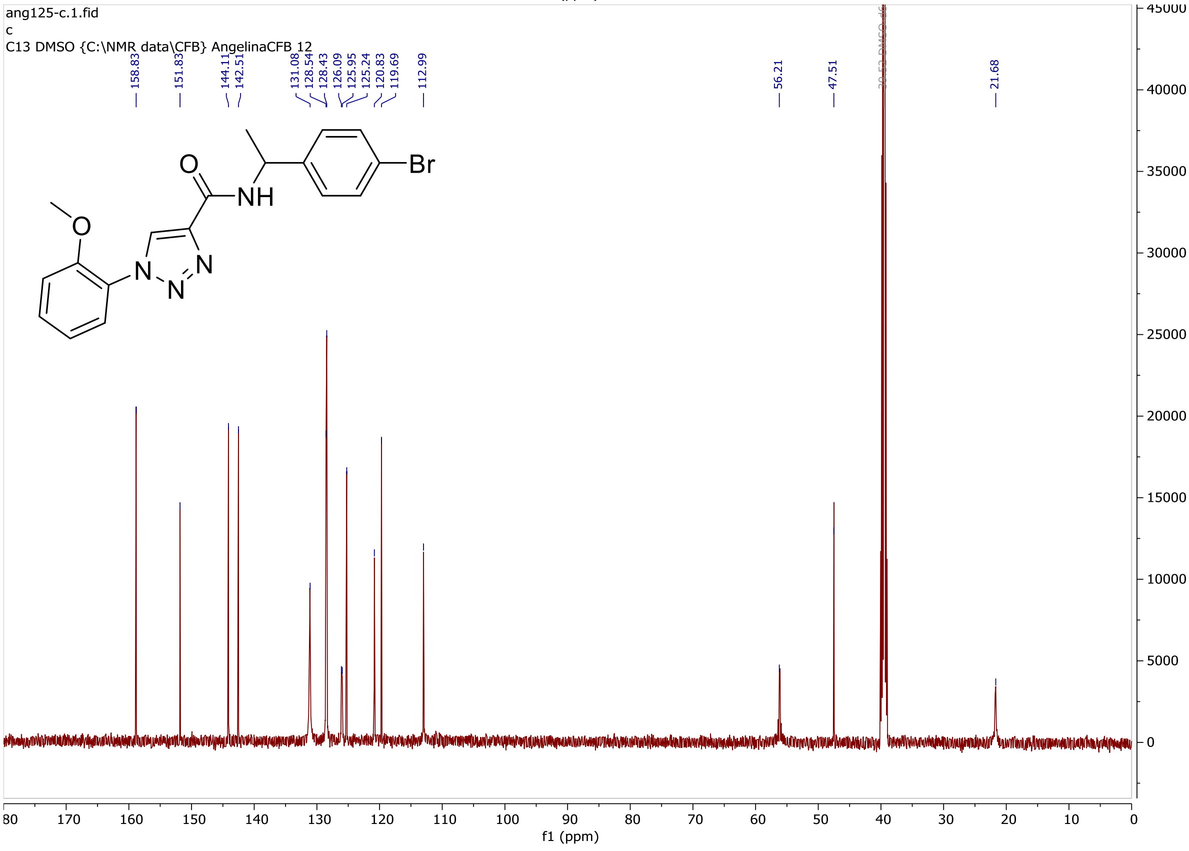

^{1}H and ^{13}C NMR spectra of compound 6f (MKA102)

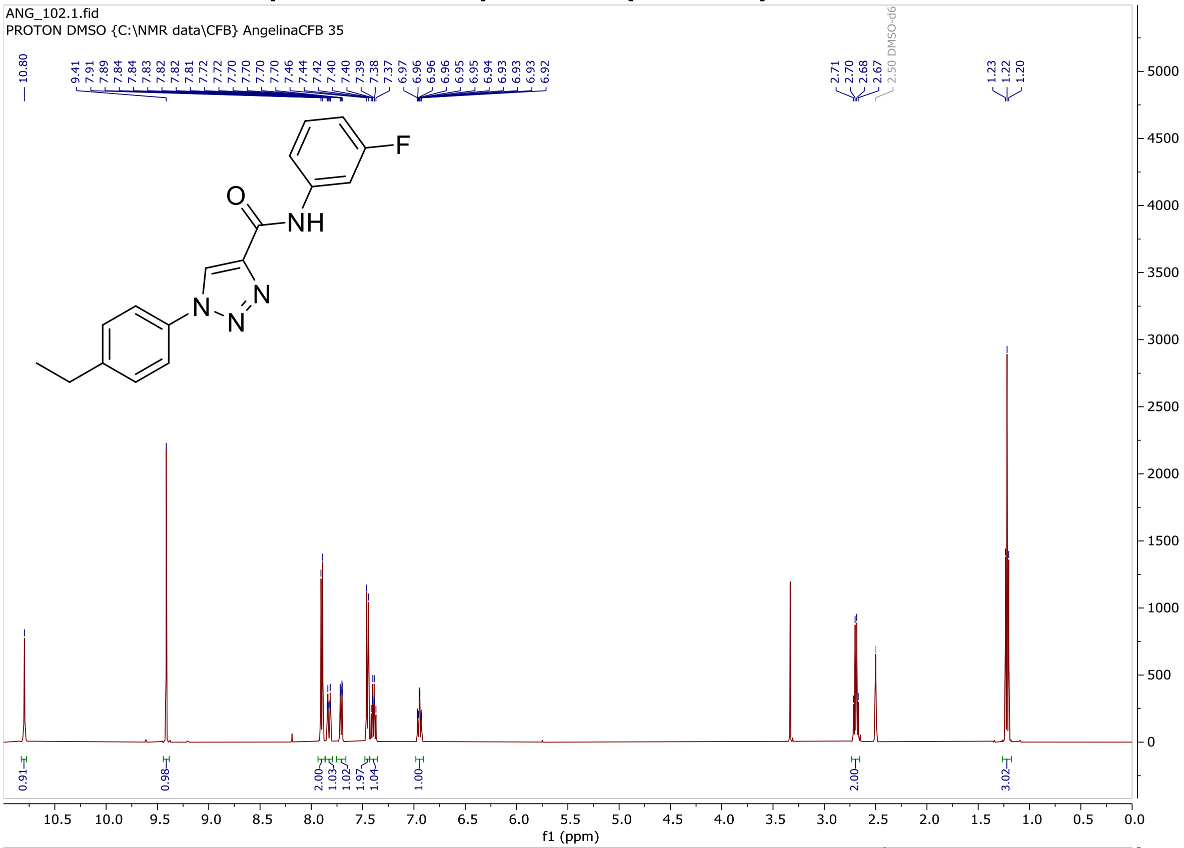

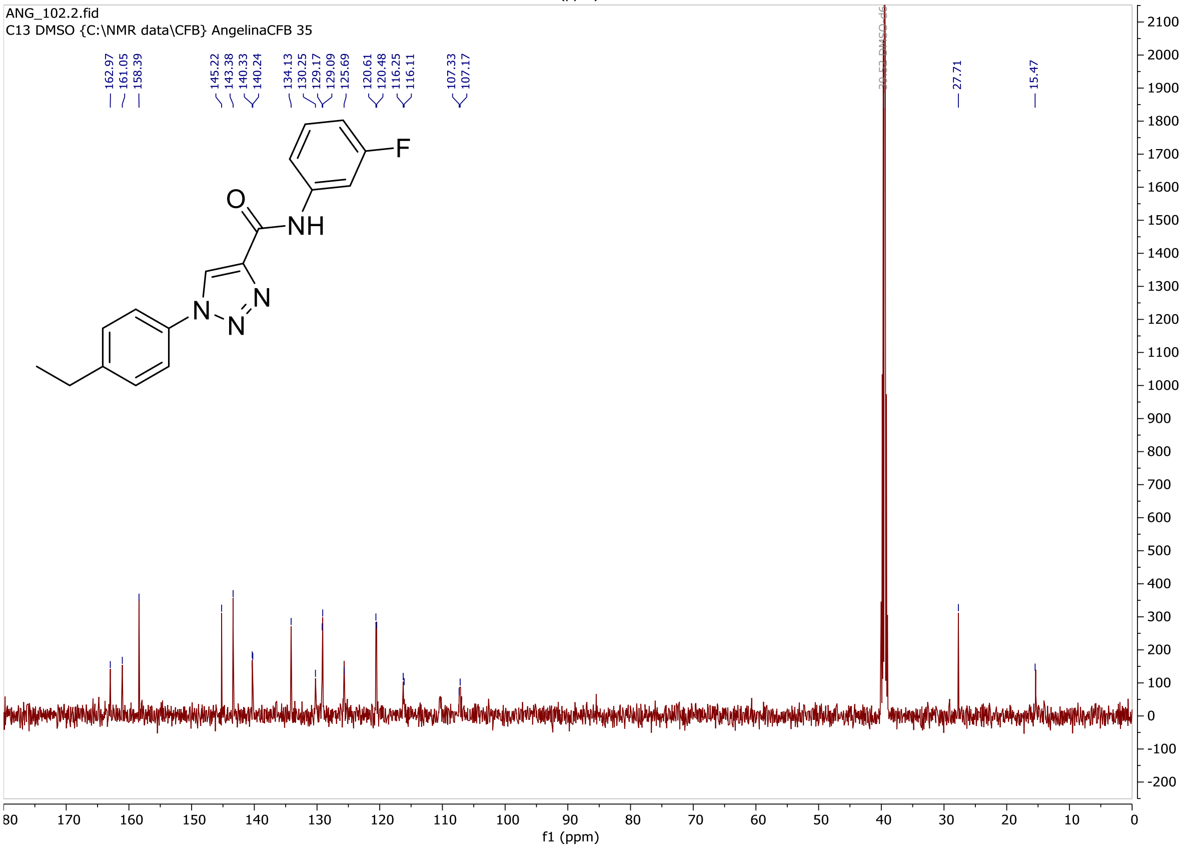

^{1}H and ^{13}C NMR spectra of compound 6g (MKA095)

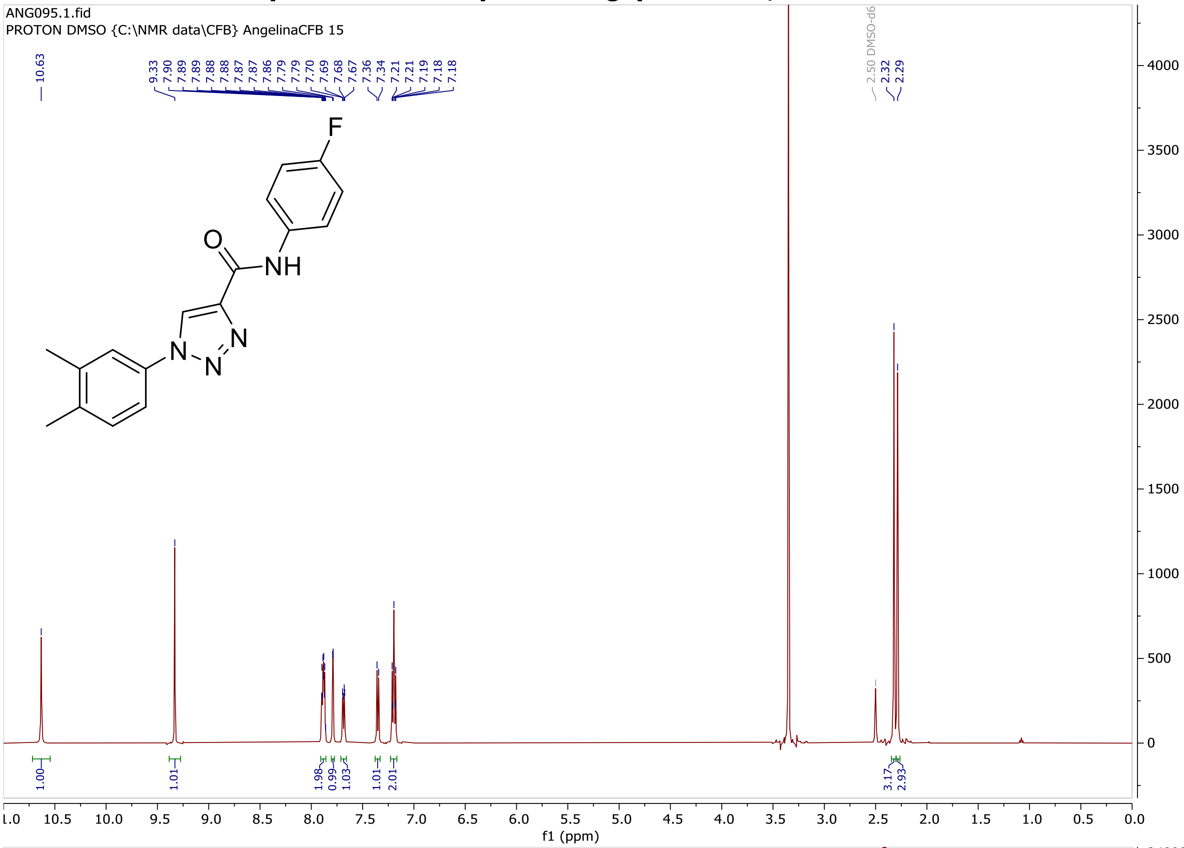

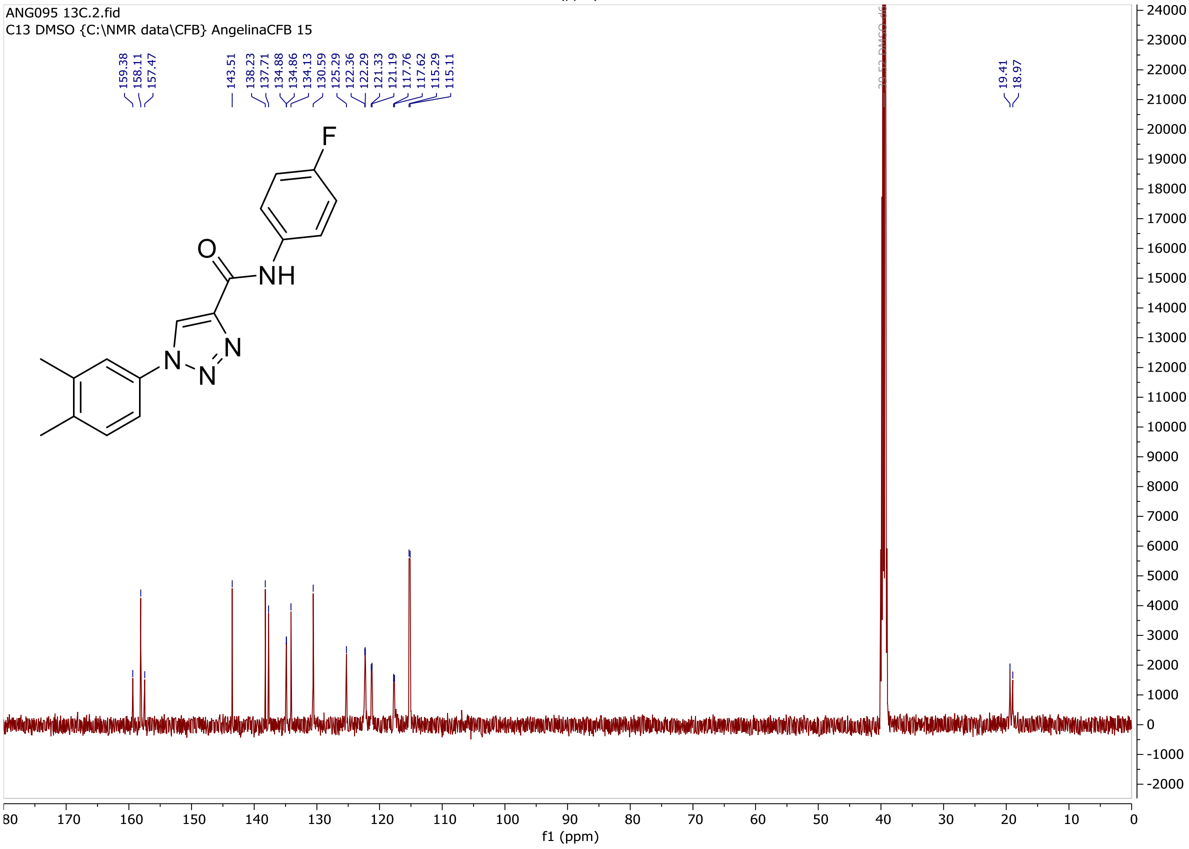

^{1}H and ^{13}C NMR spectra of compound 6h (MKA030)

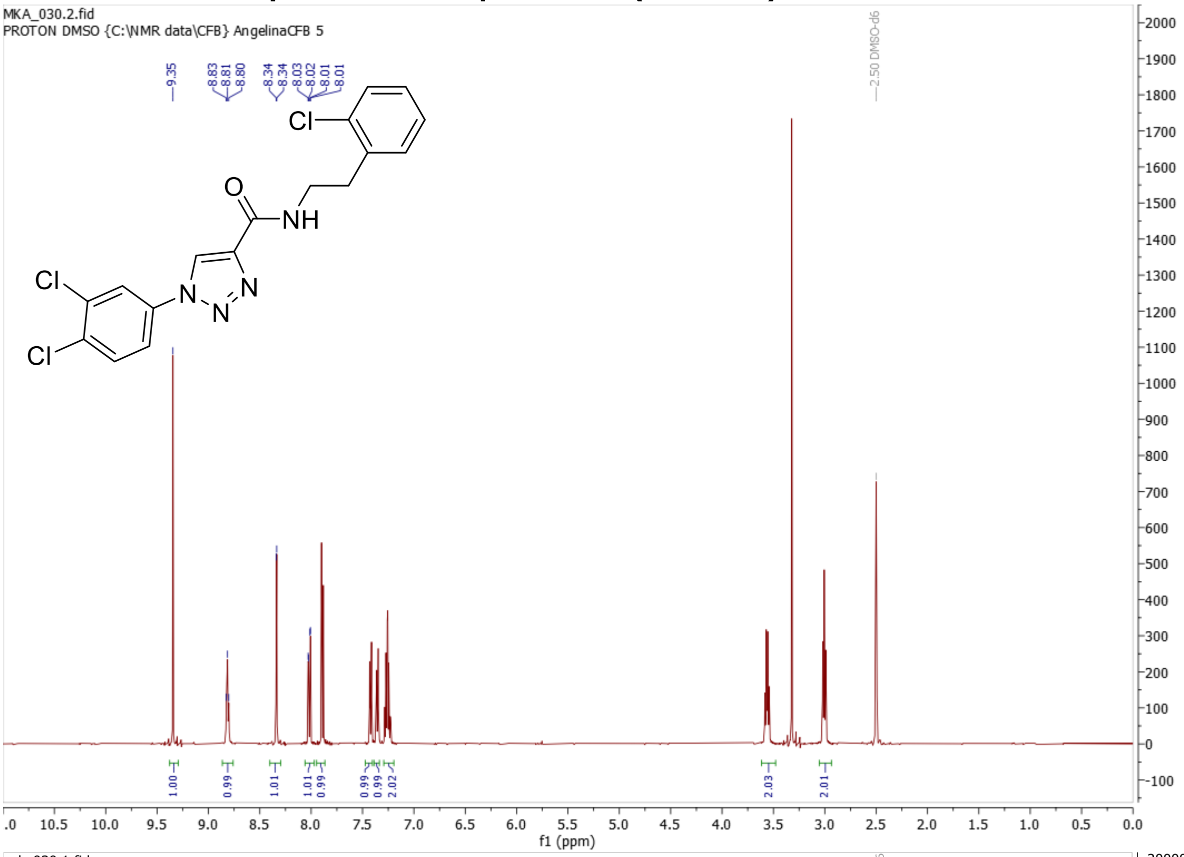

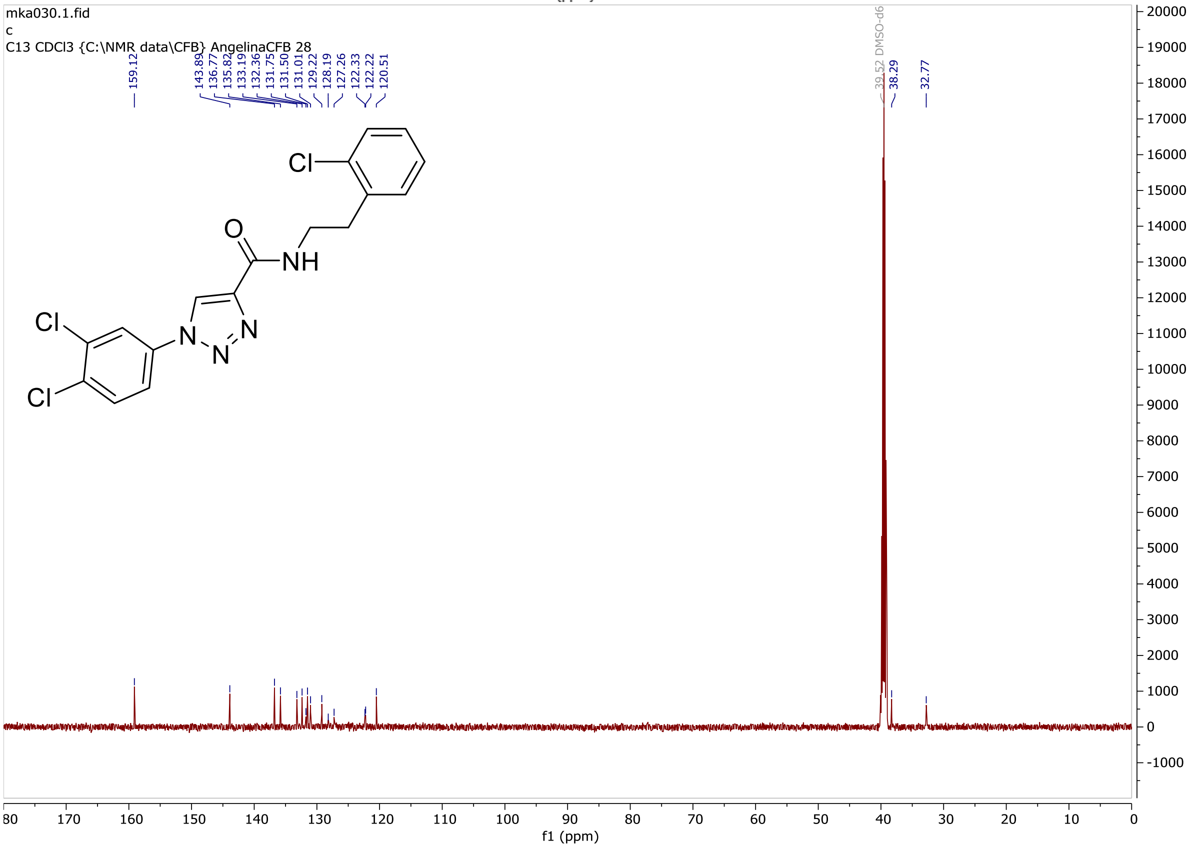

^{1}H and ^{13}C NMR spectra of compound 6i (MKA044)

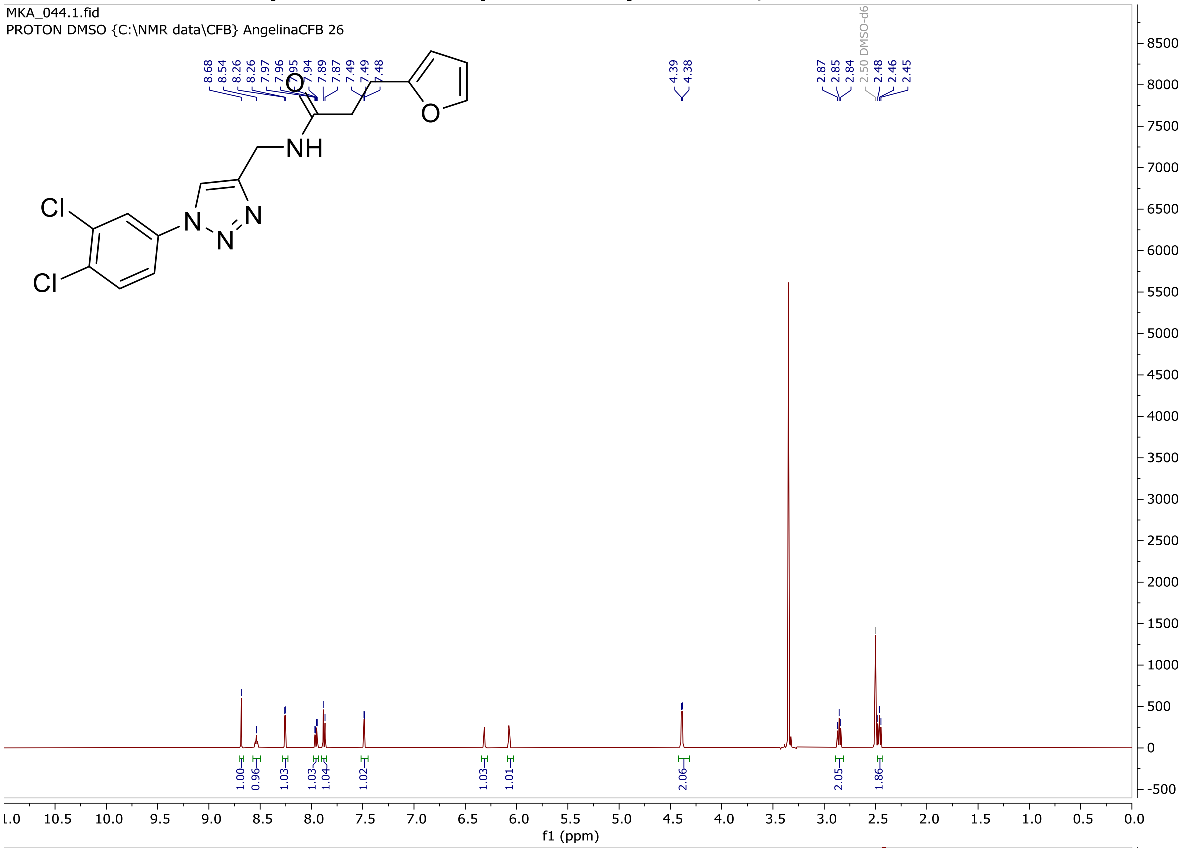

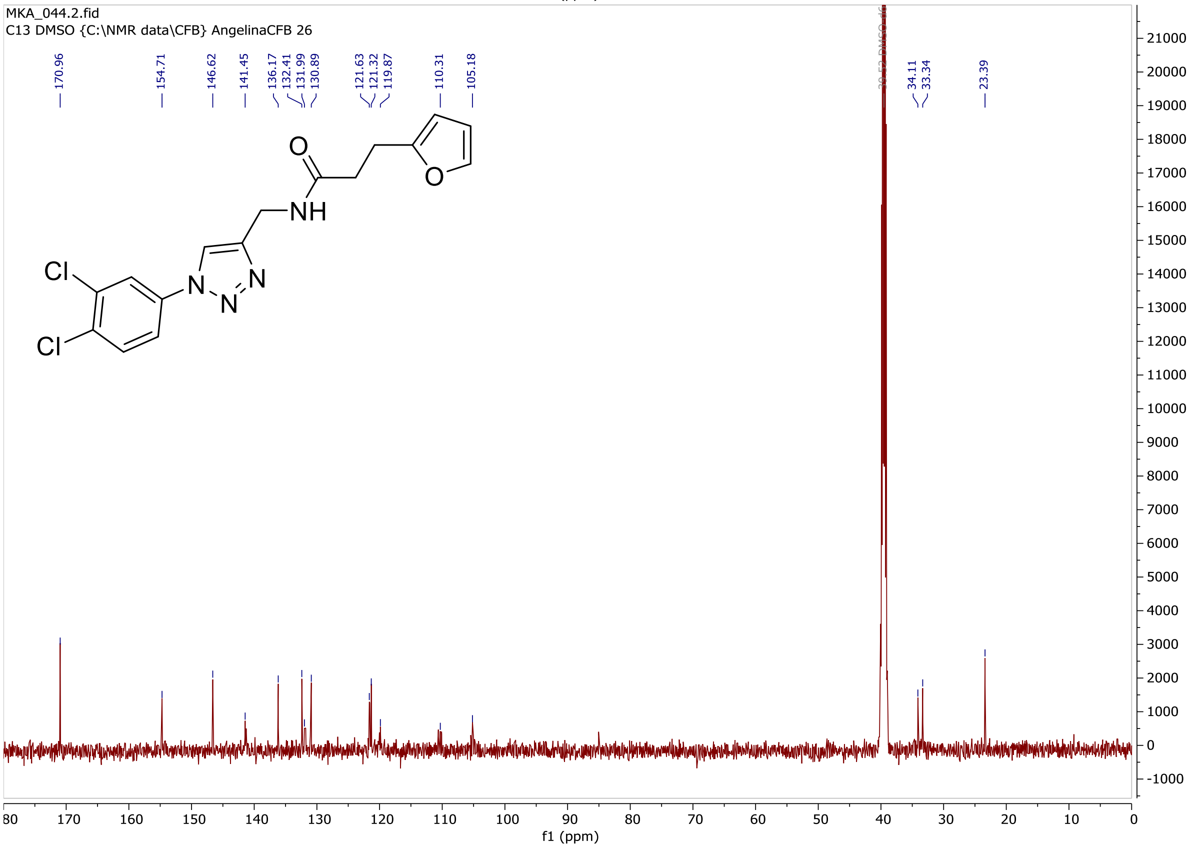

^{1}H and ^{13}C NMR spectra of compound 6j (MKA084)

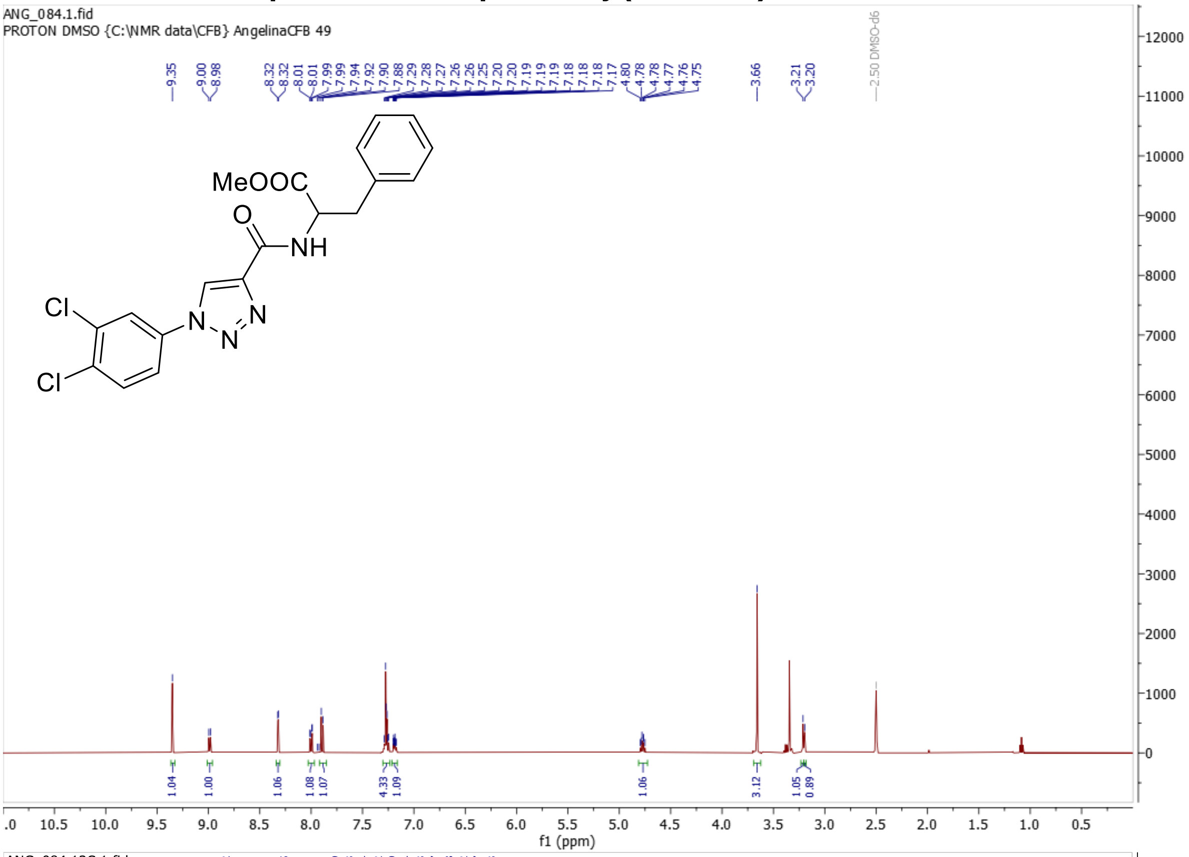

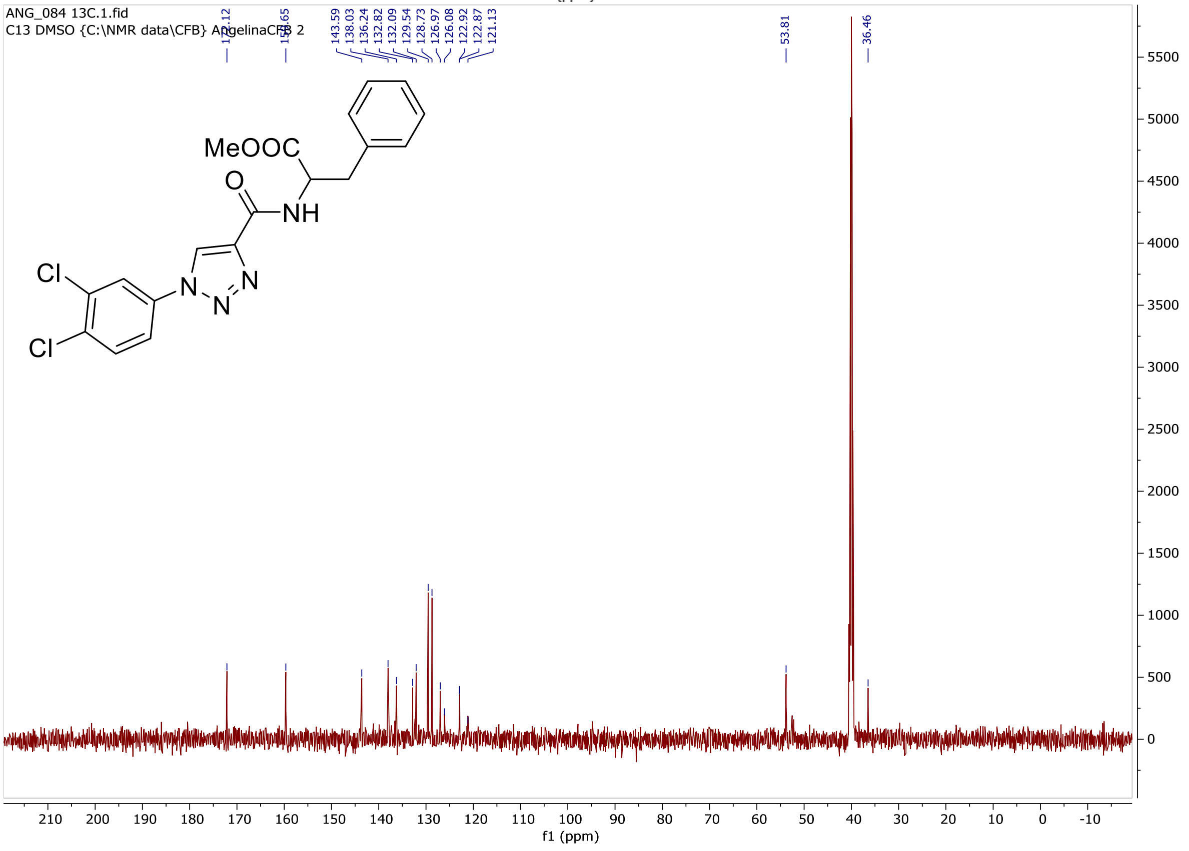

^{1}H and ^{13}C NMR spectra of compound 6k (MKA085)

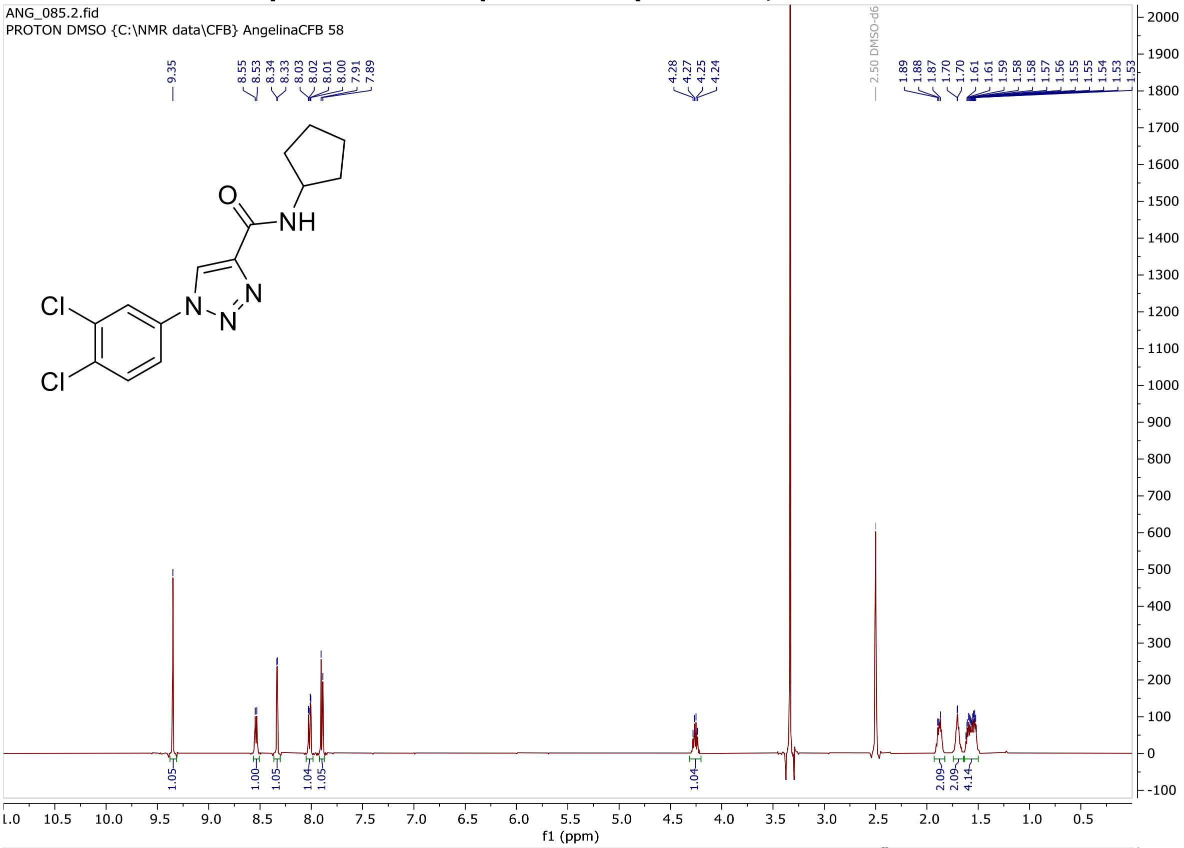

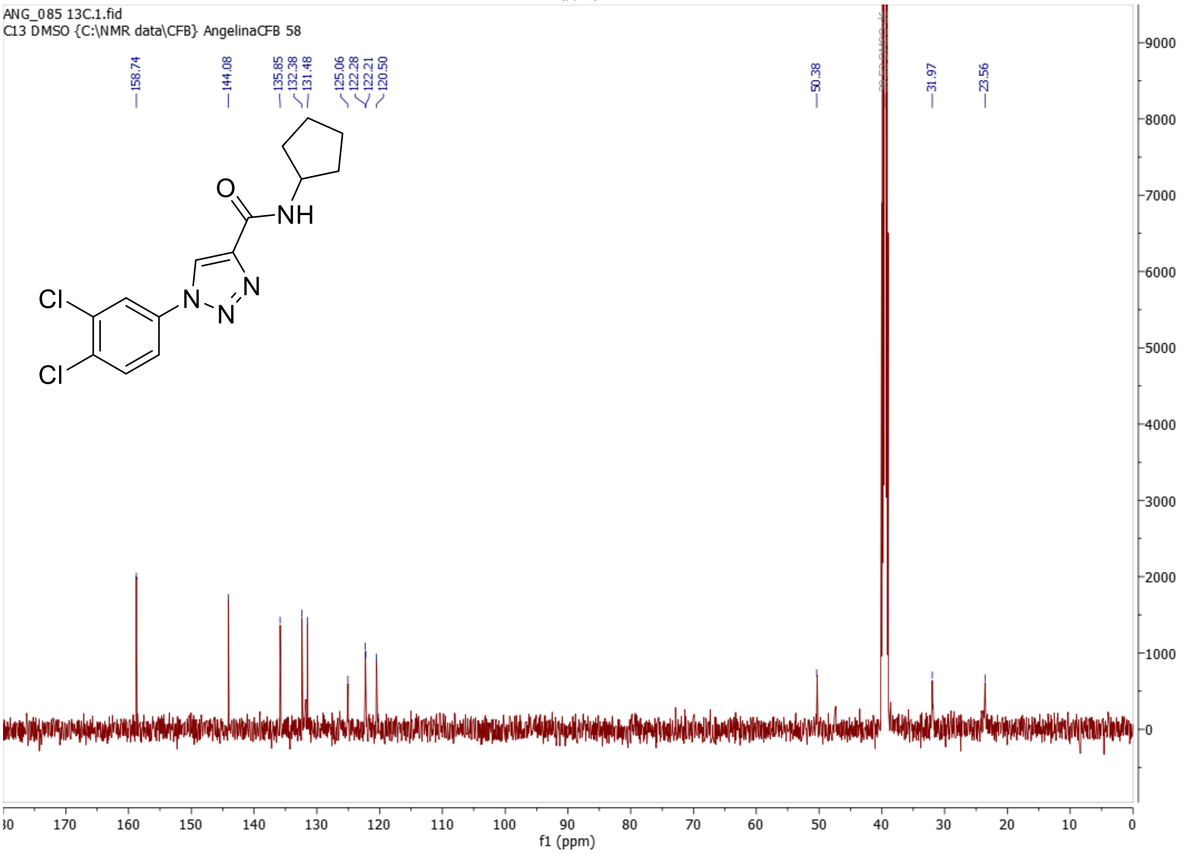

^{1}H and ^{13}C NMR spectra of compound 6l (MKA038)

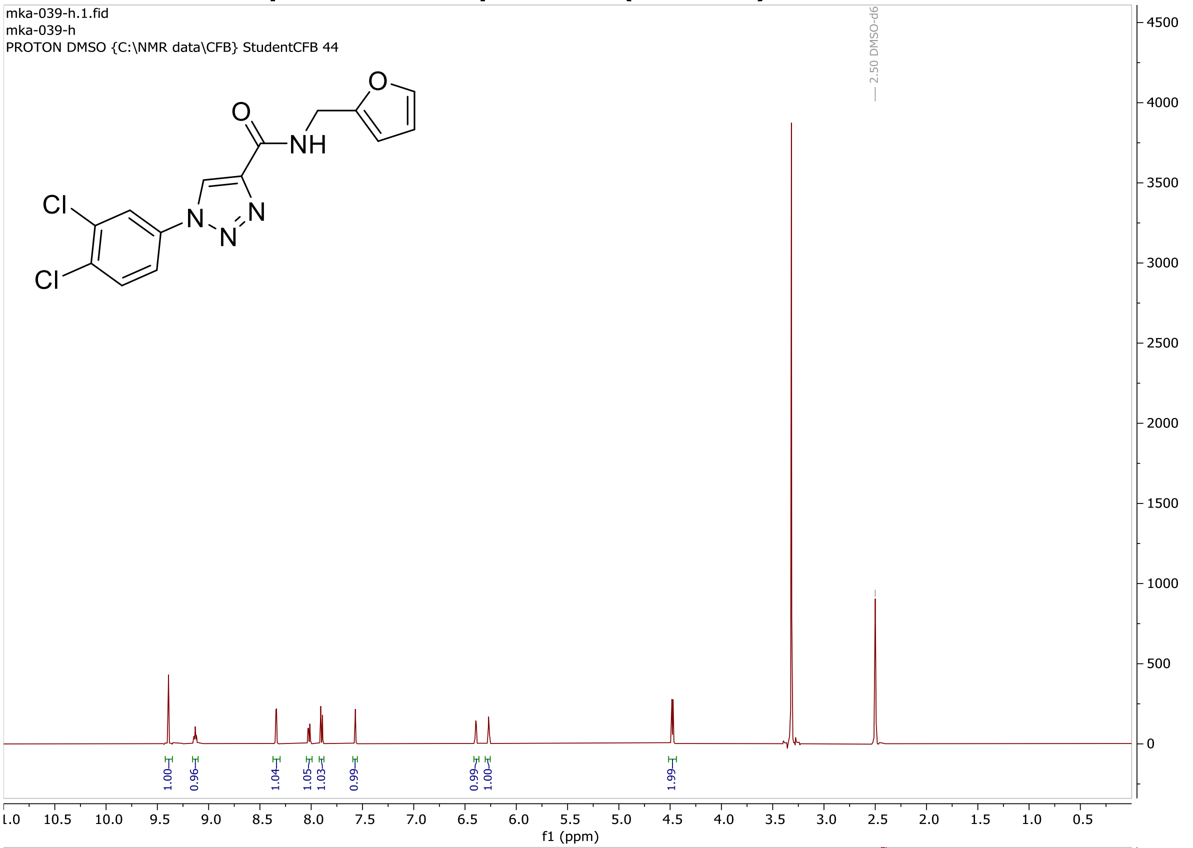

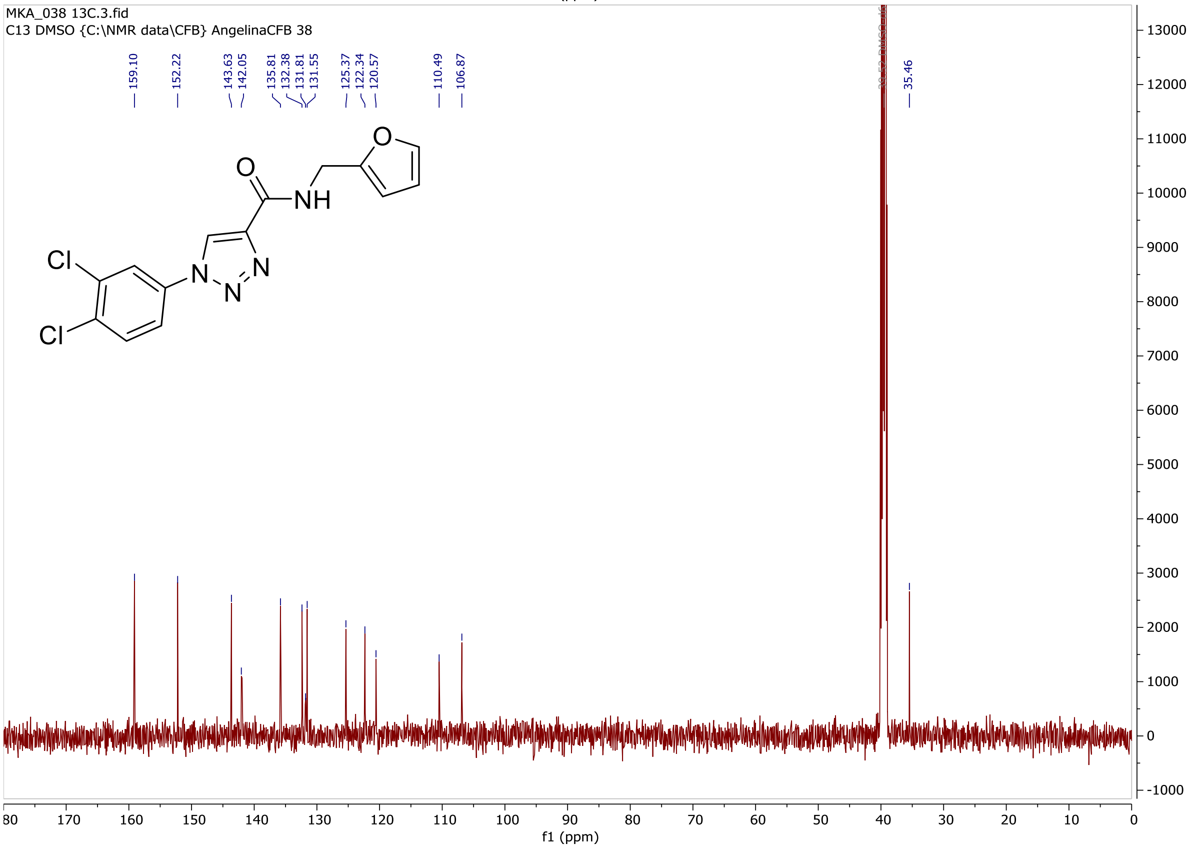

^{1}H and ^{13}C NMR spectra of compound 6m (MKA029)

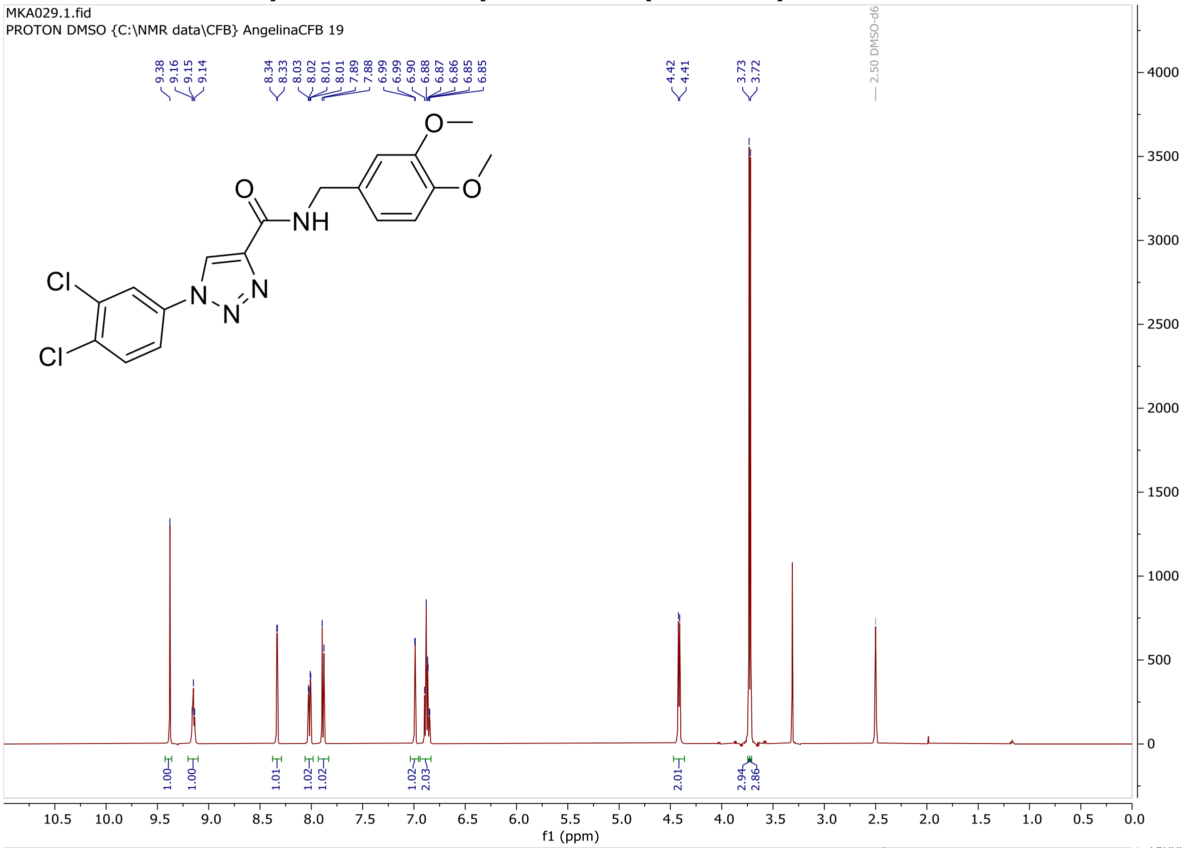

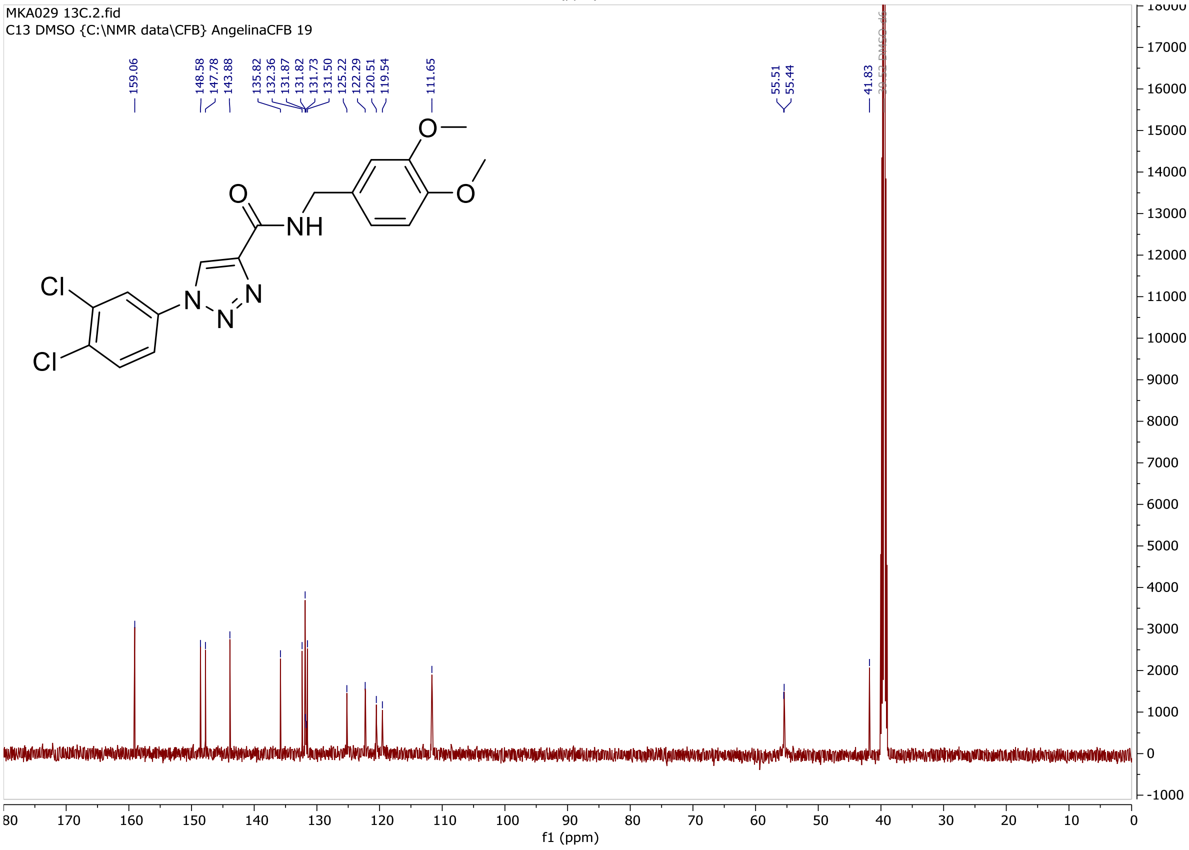

^{1}H and ^{13}C NMR spectra of compound 6n (MKA106)

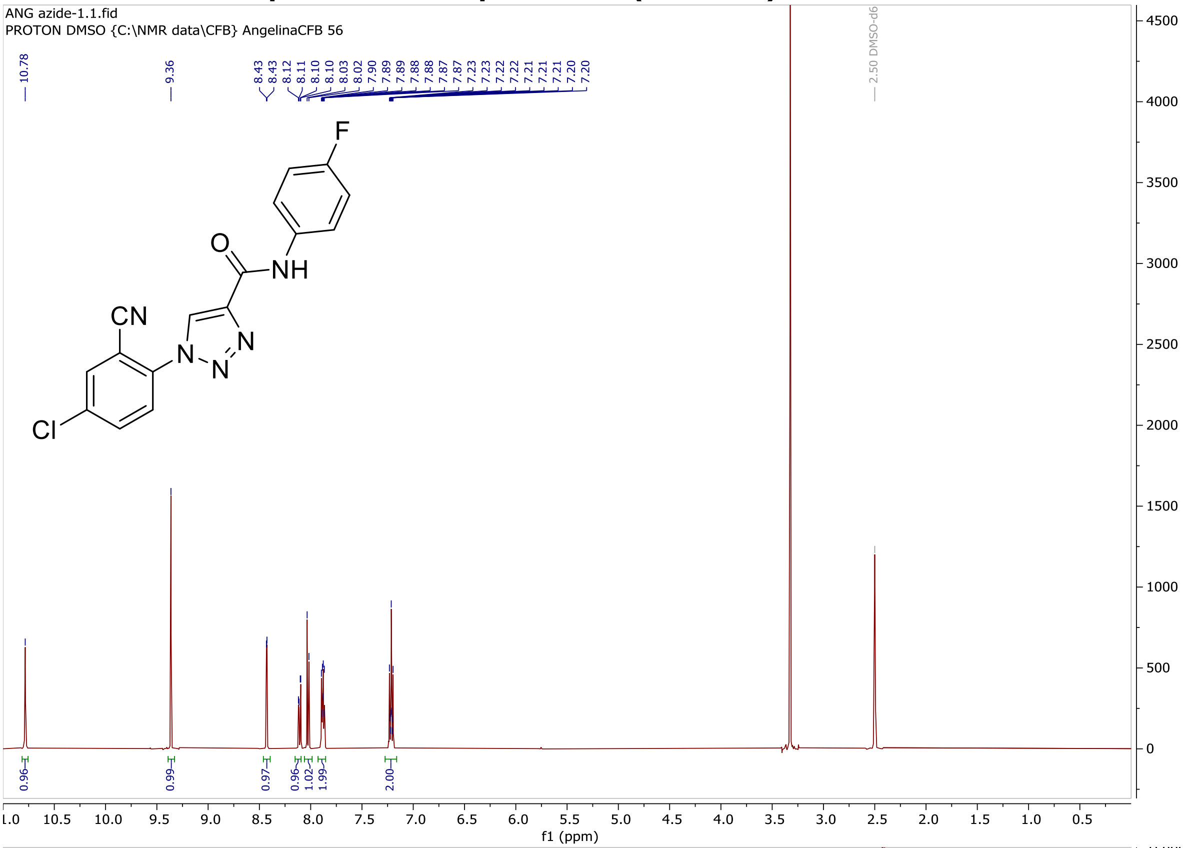

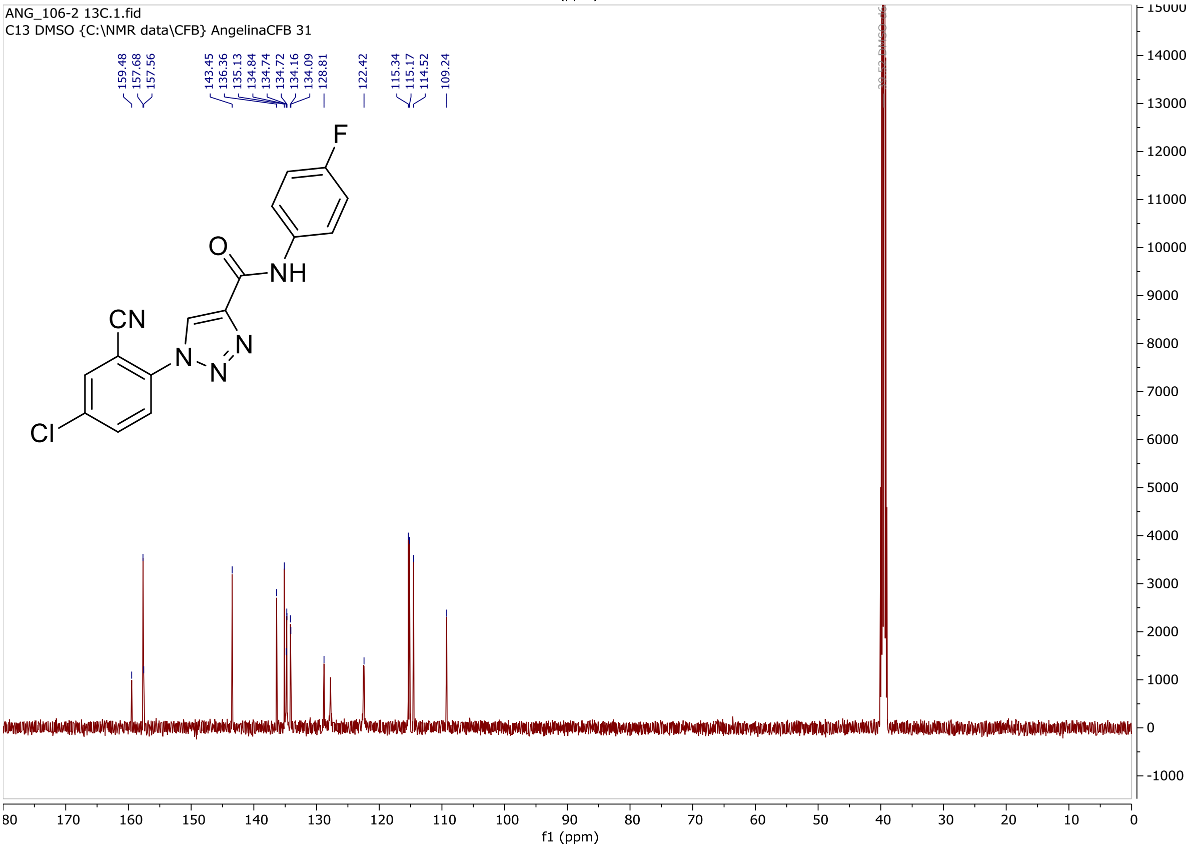

^{1}H and ^{13}C NMR spectra of compound 6o (MKA097)

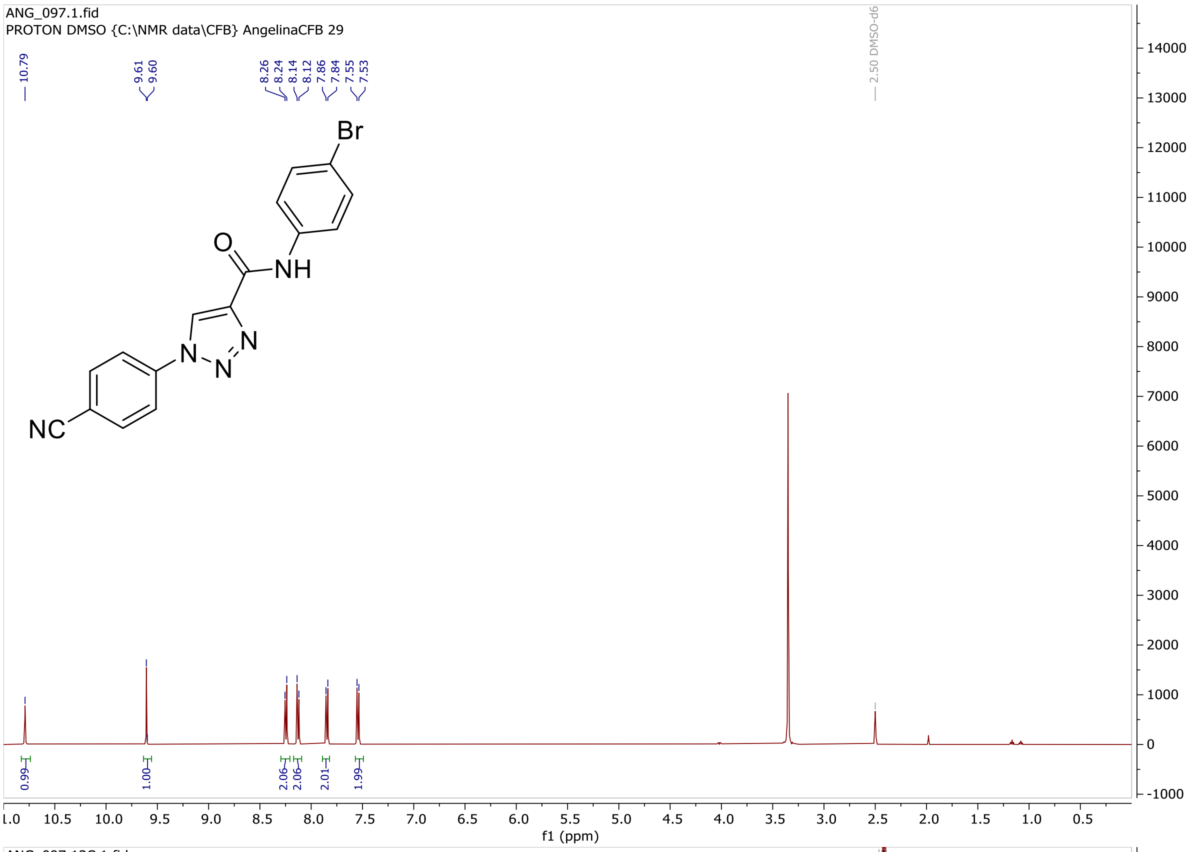

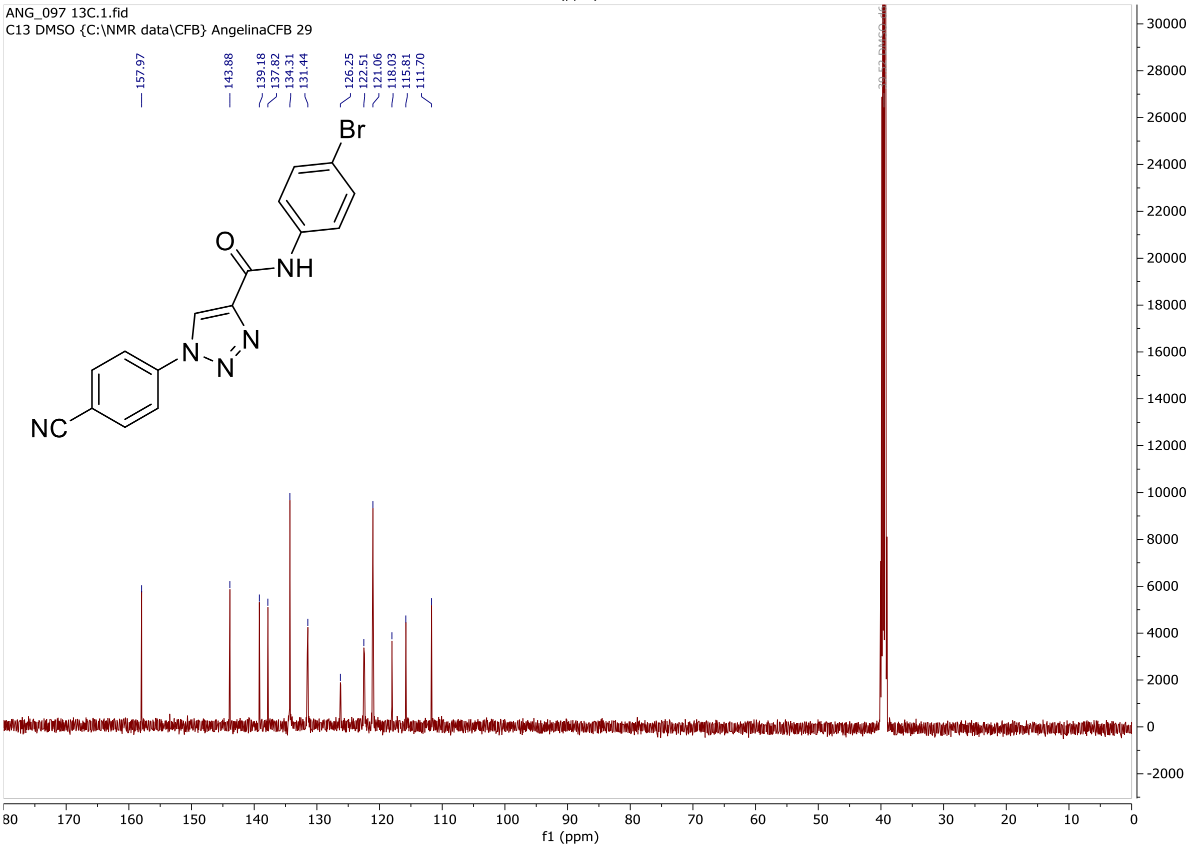

^{1}H and ^{13}C NMR spectra of compound 6p (MKA098)

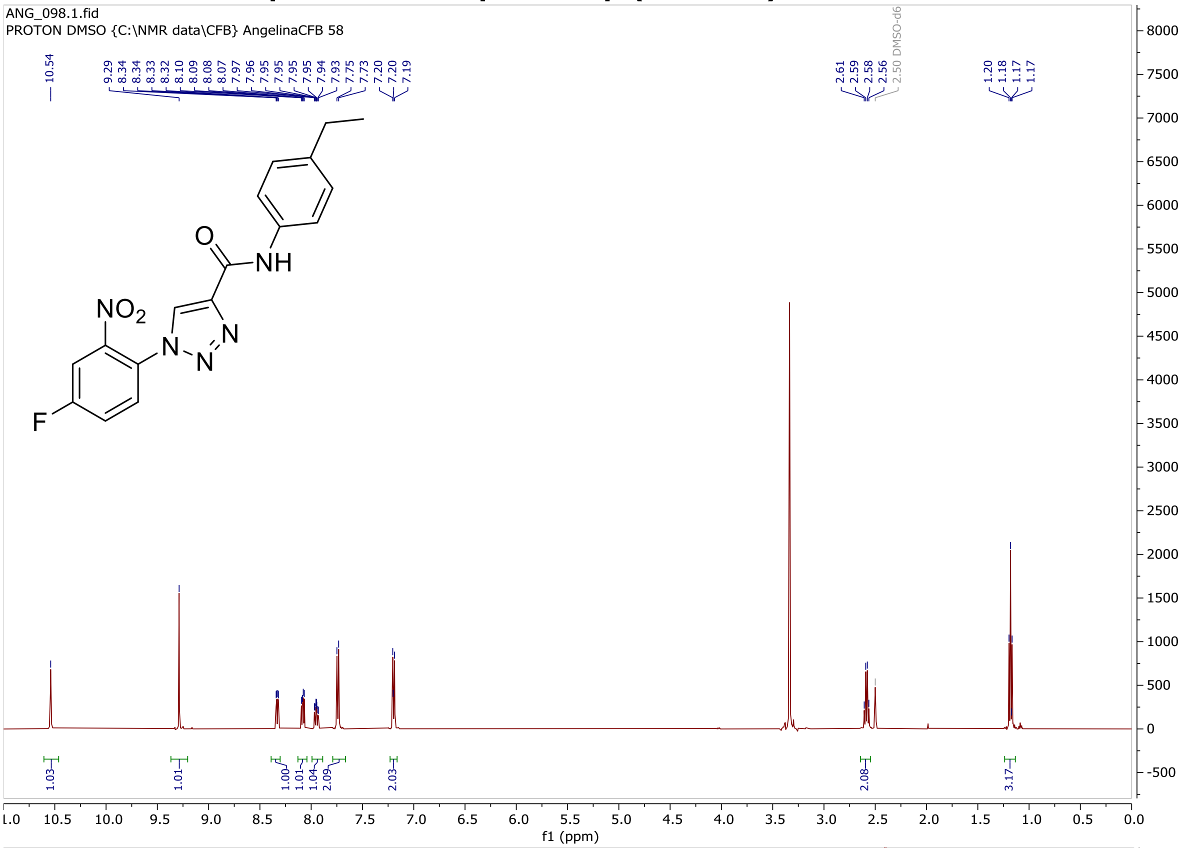

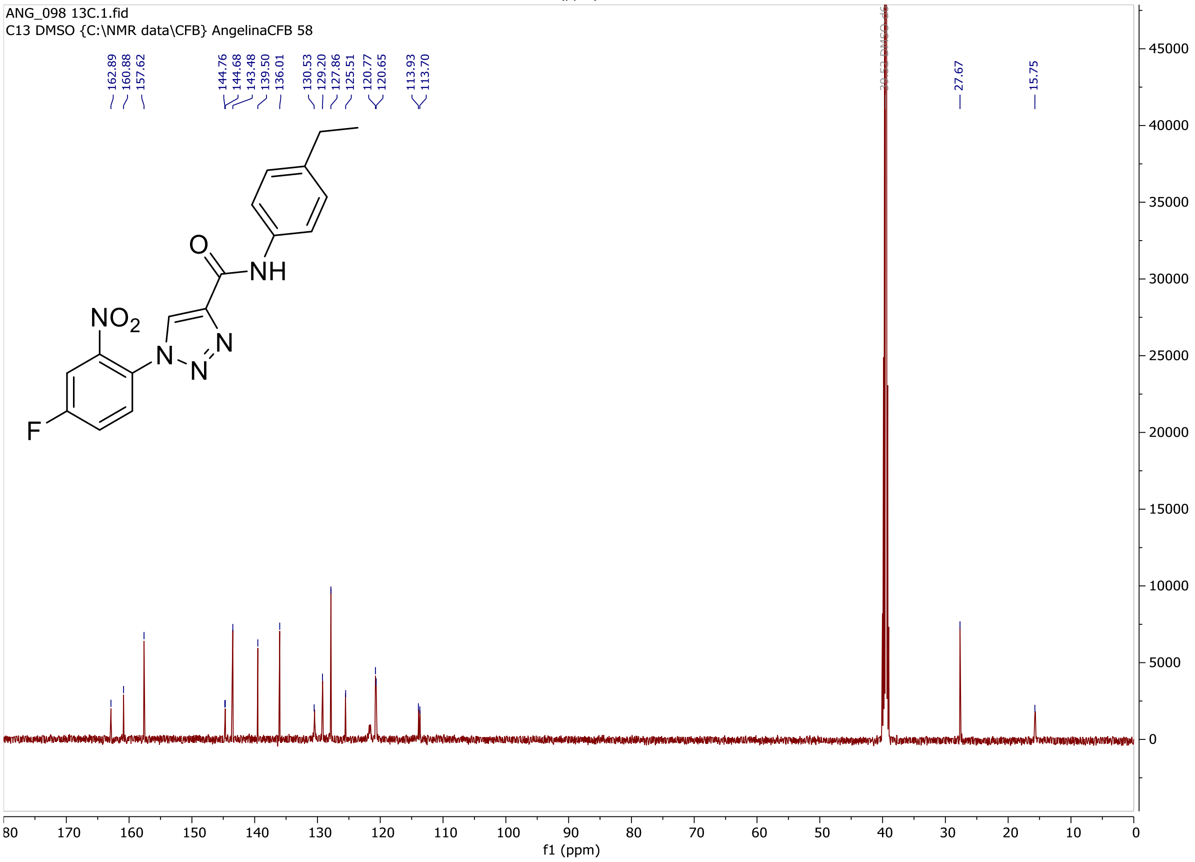

^{1}H and ^{13}C NMR spectra of compound 6q (MKA004)

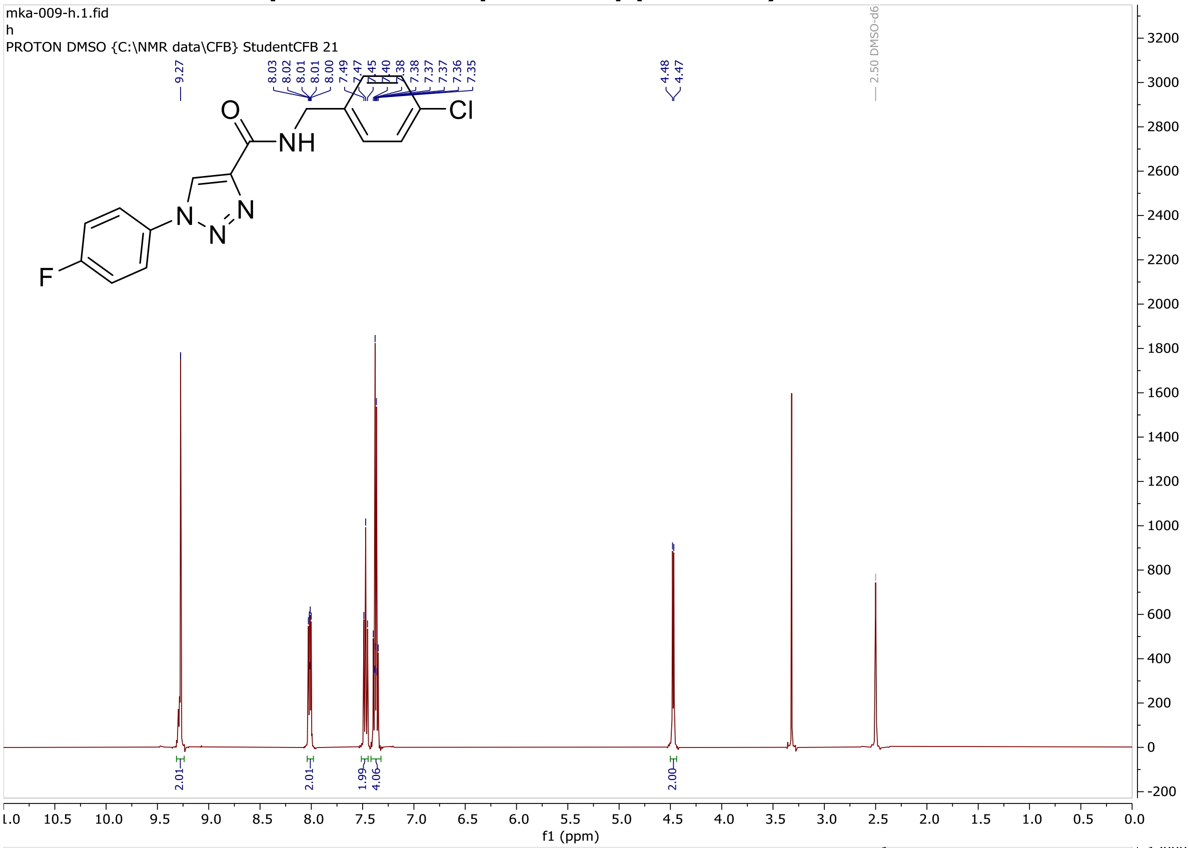

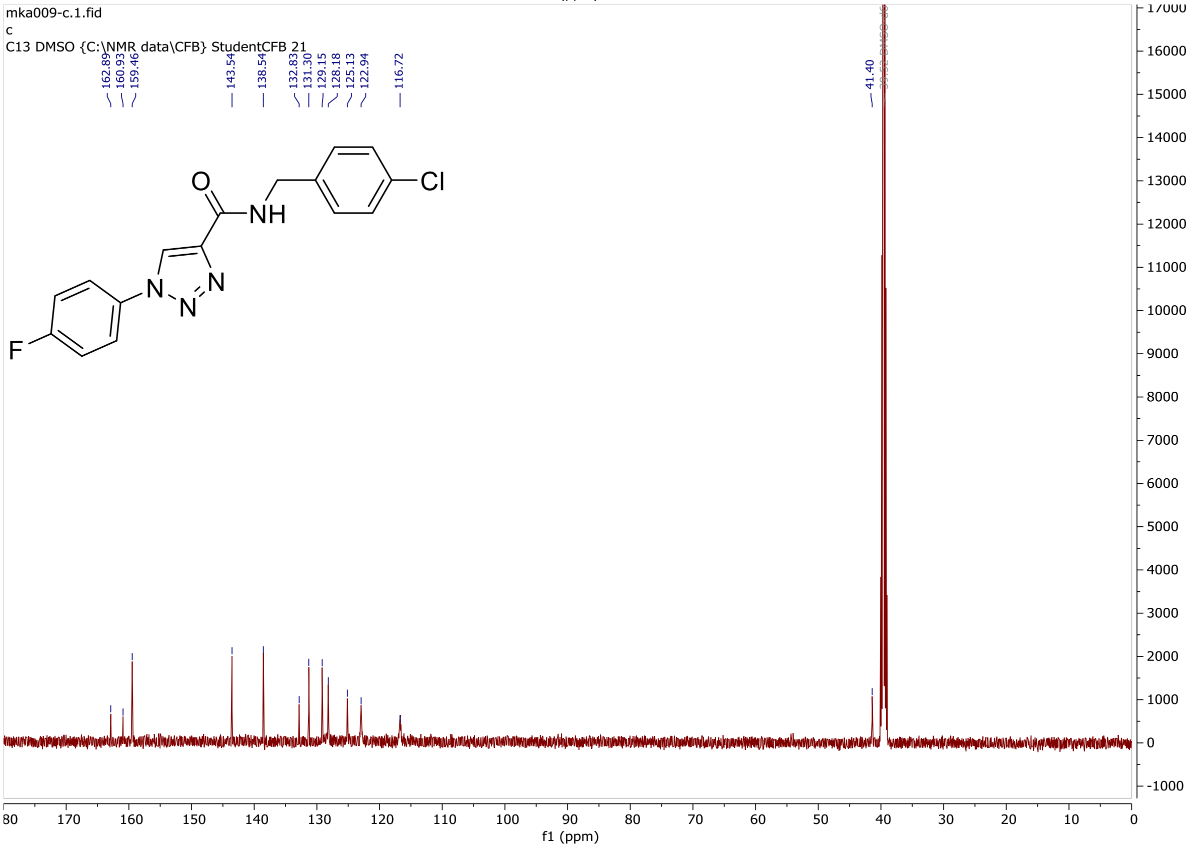

^{1}H and ^{13}C NMR spectra of compound 6r (MKA010)

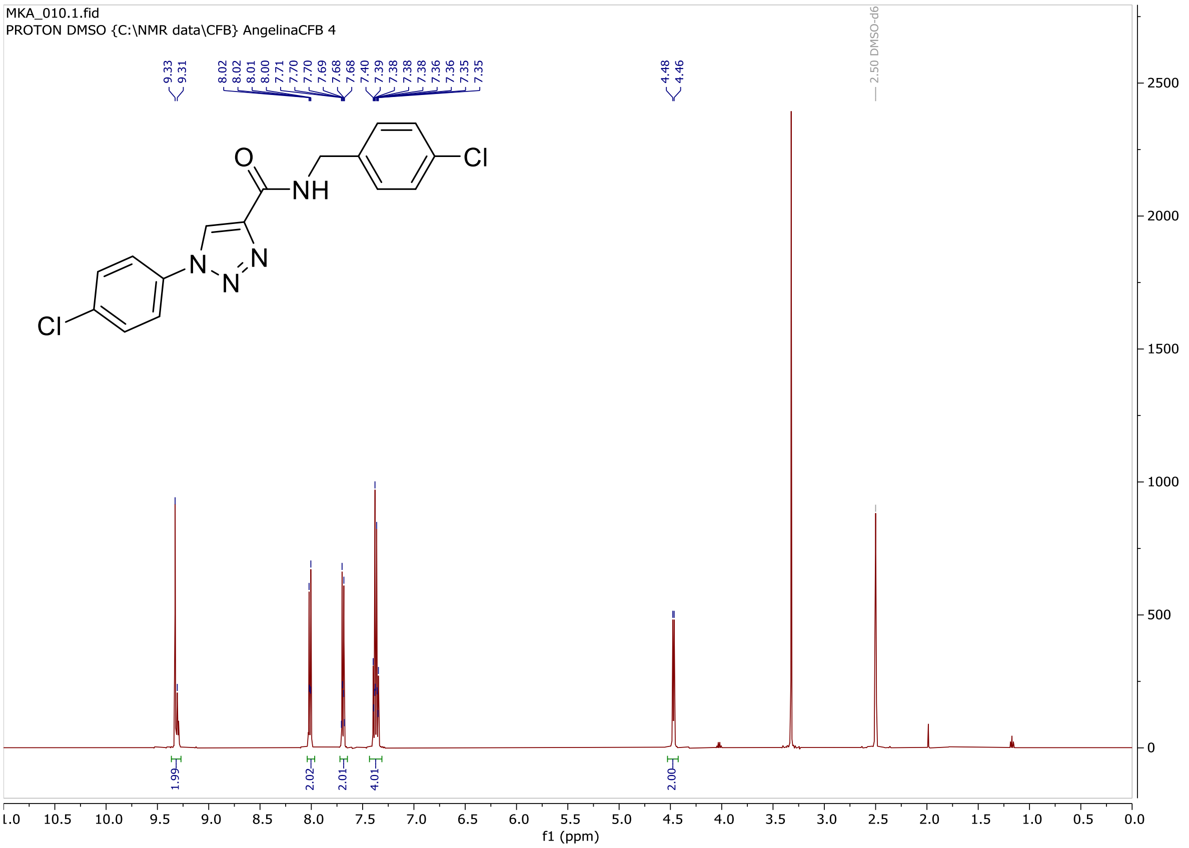

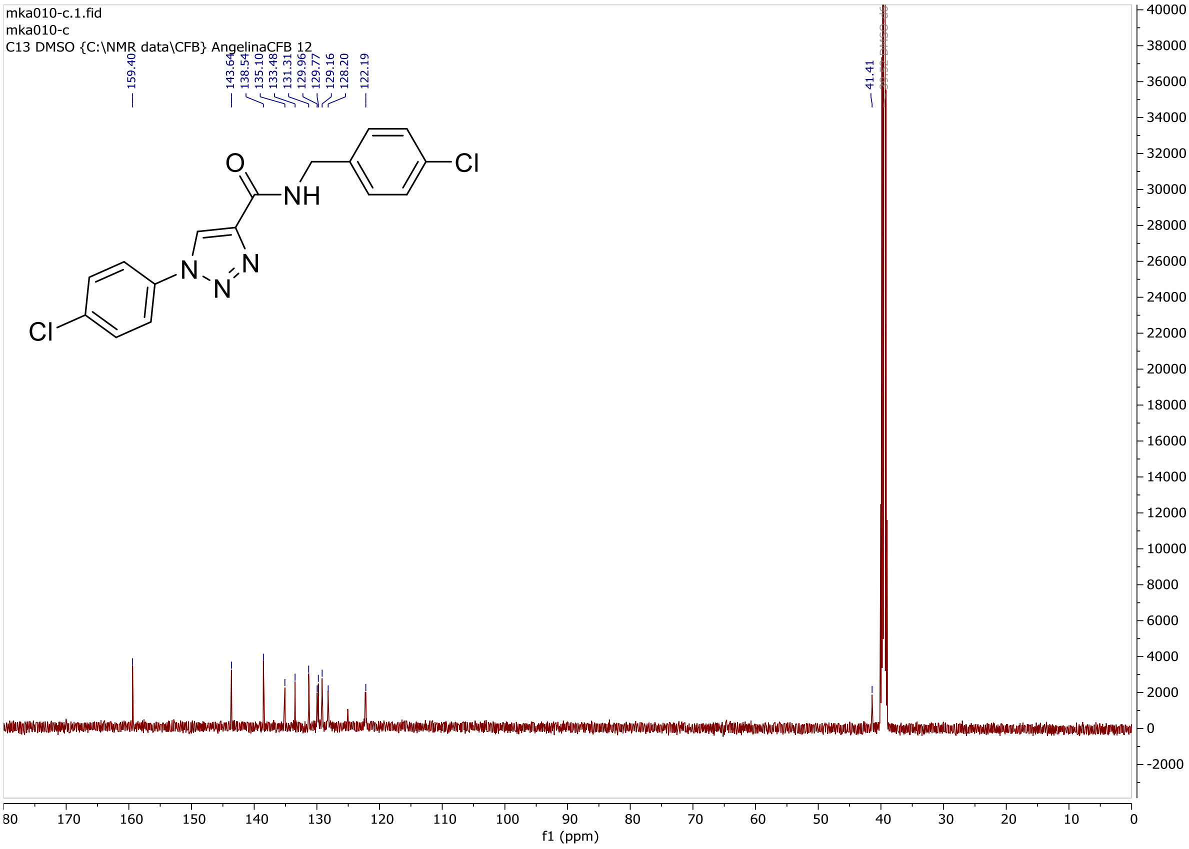

^{1}H and ^{13}C NMR spectra of compound 6s (MKA048)

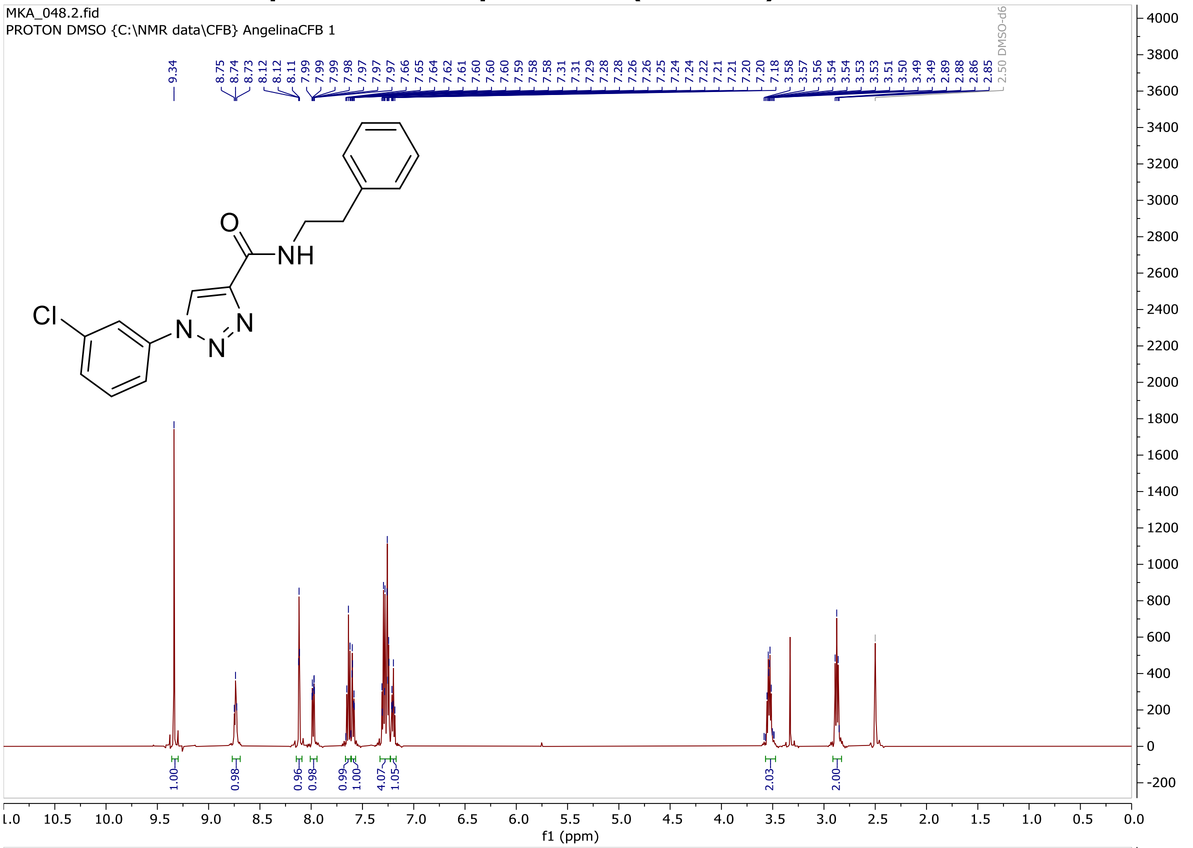

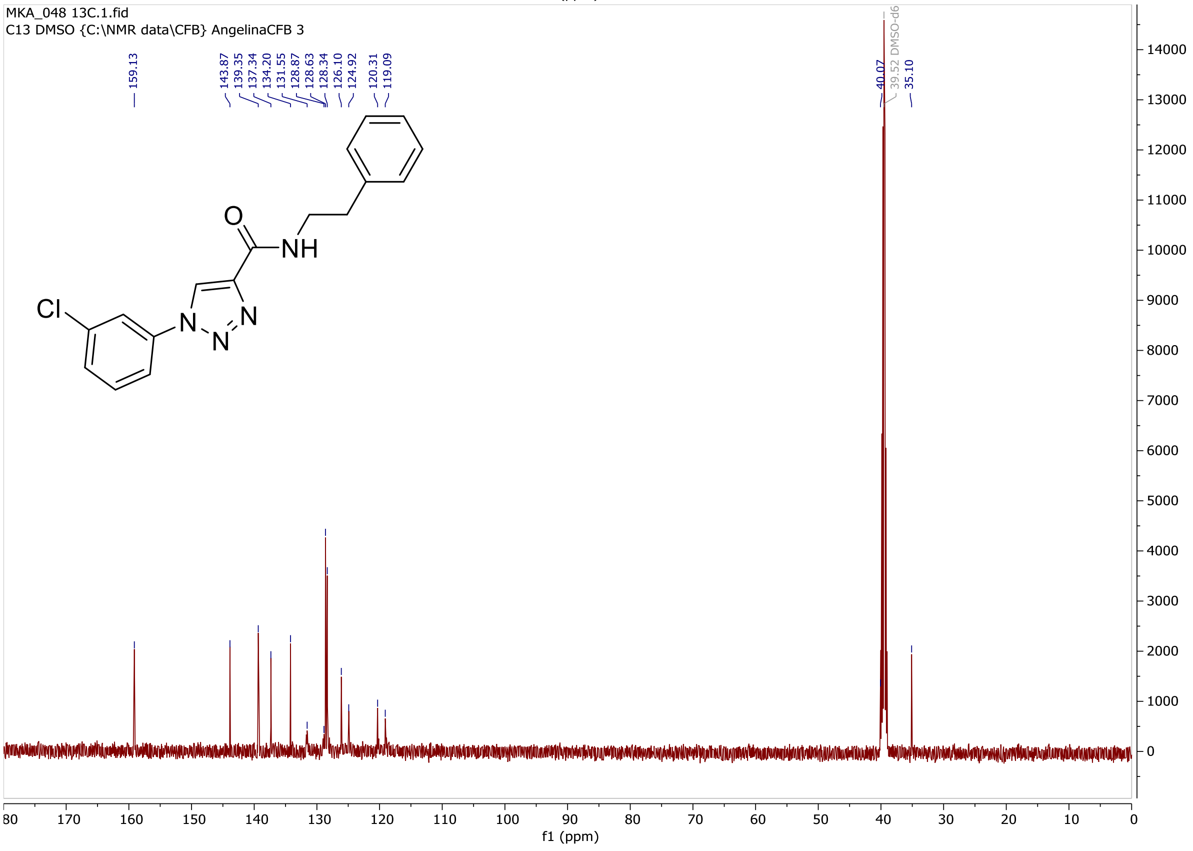

^{1}H and ^{13}C NMR spectra of compound 6t (MKA050)

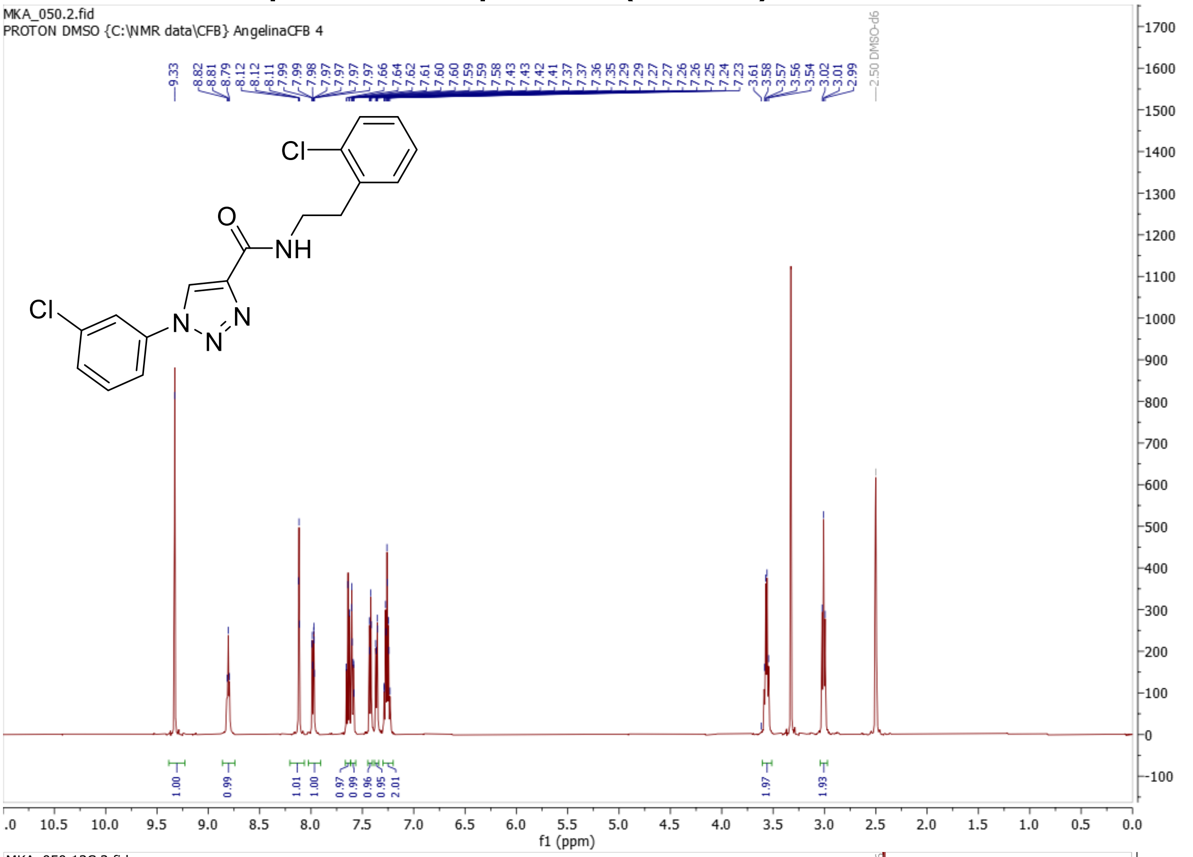

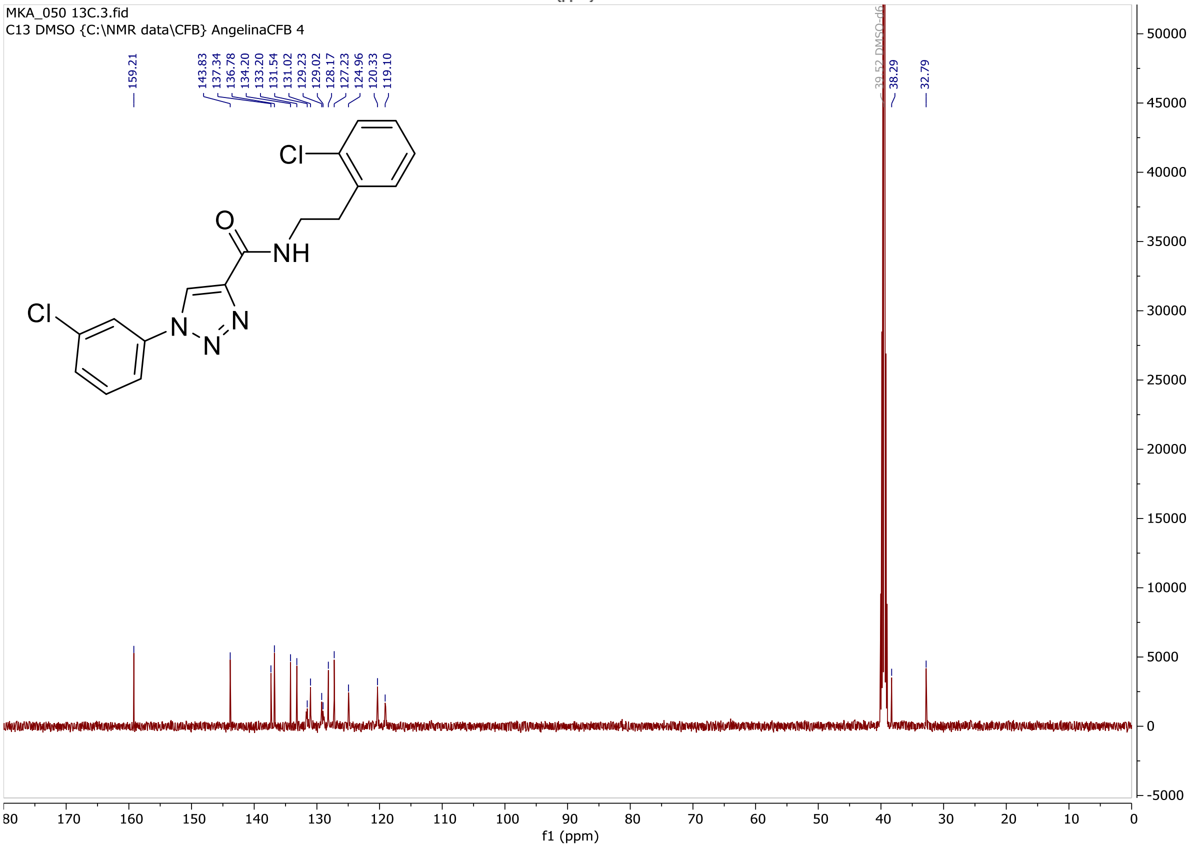

^{1}H and ^{13}C NMR spectra of compound 6u (MKA078)

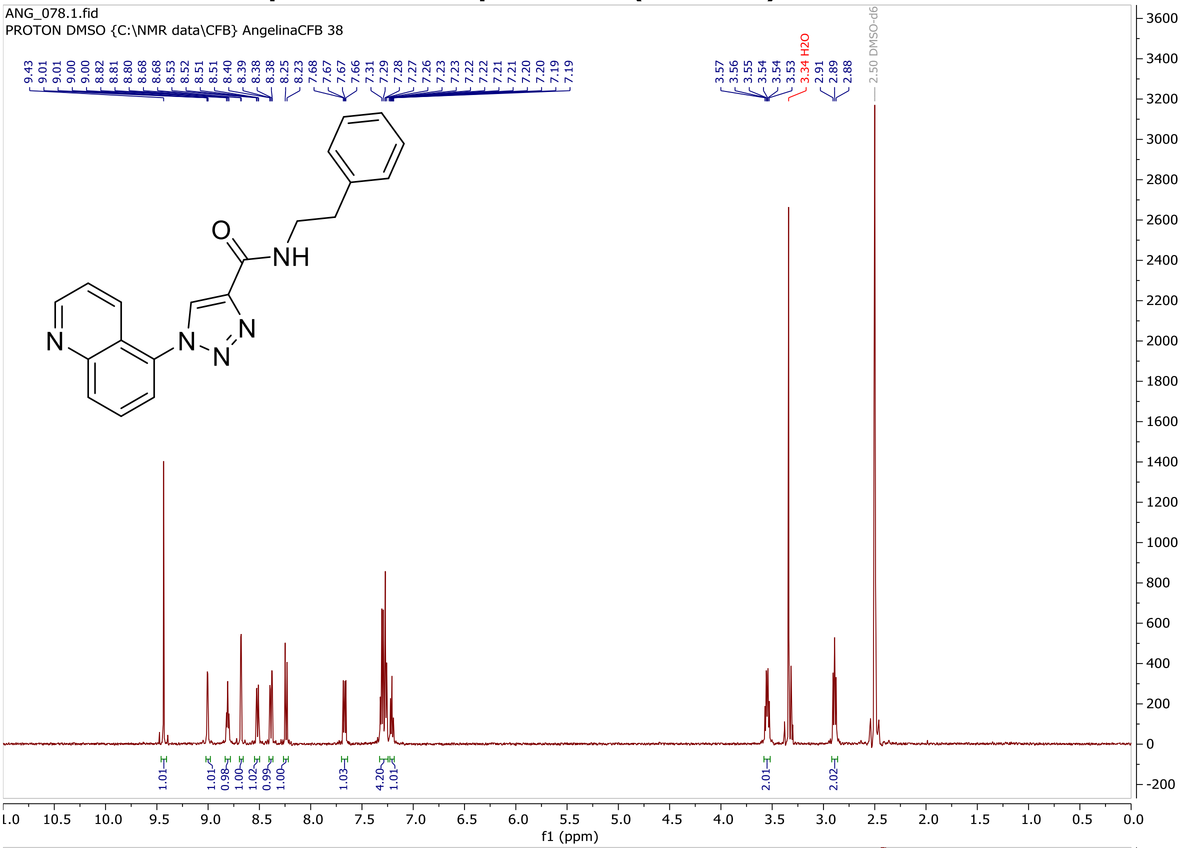

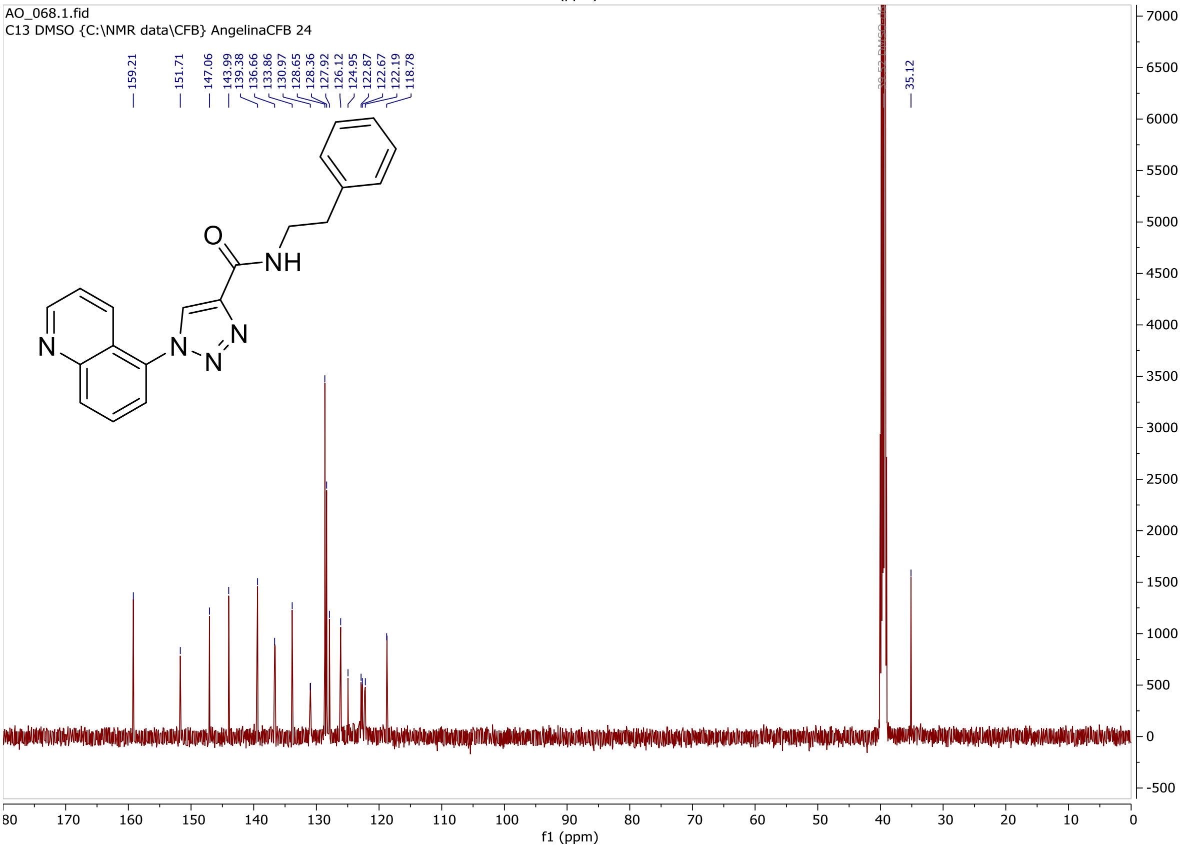

^{1}H and ^{13}C NMR spectra of compound 6v (MKA027)

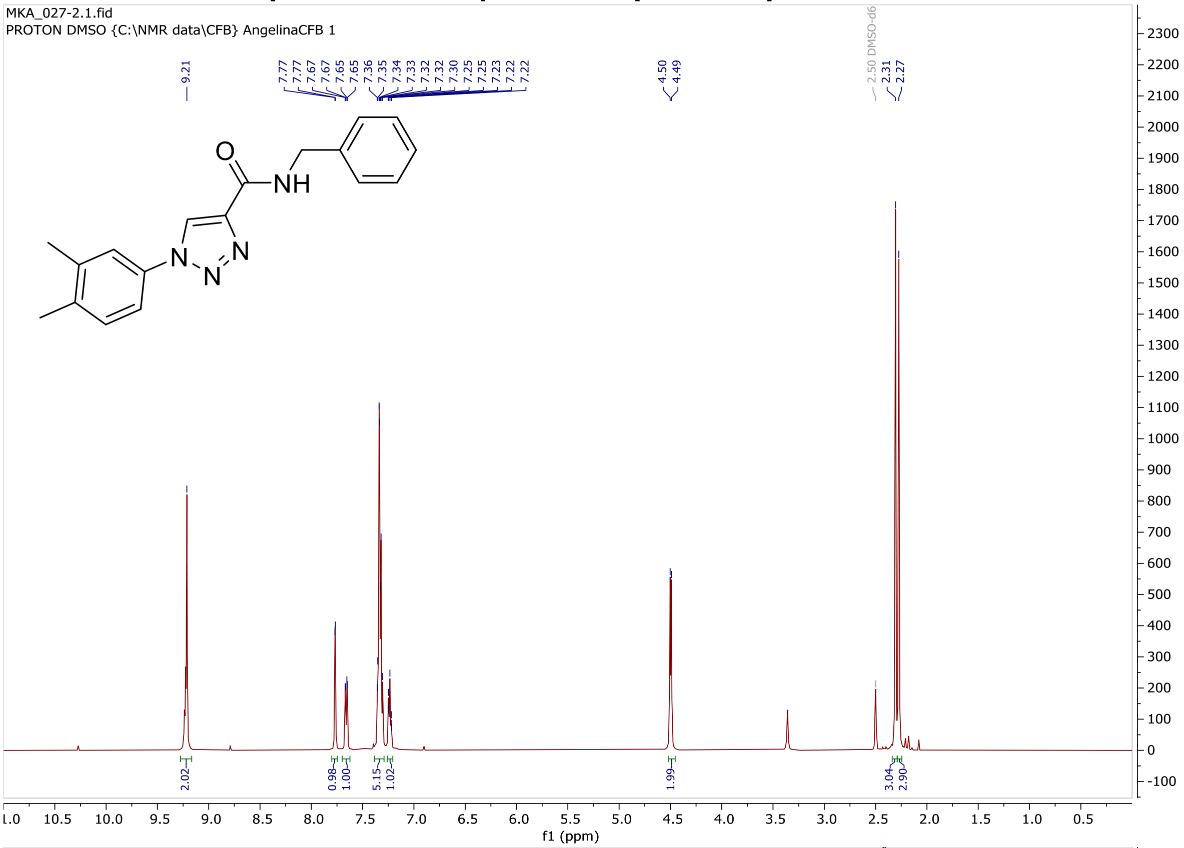

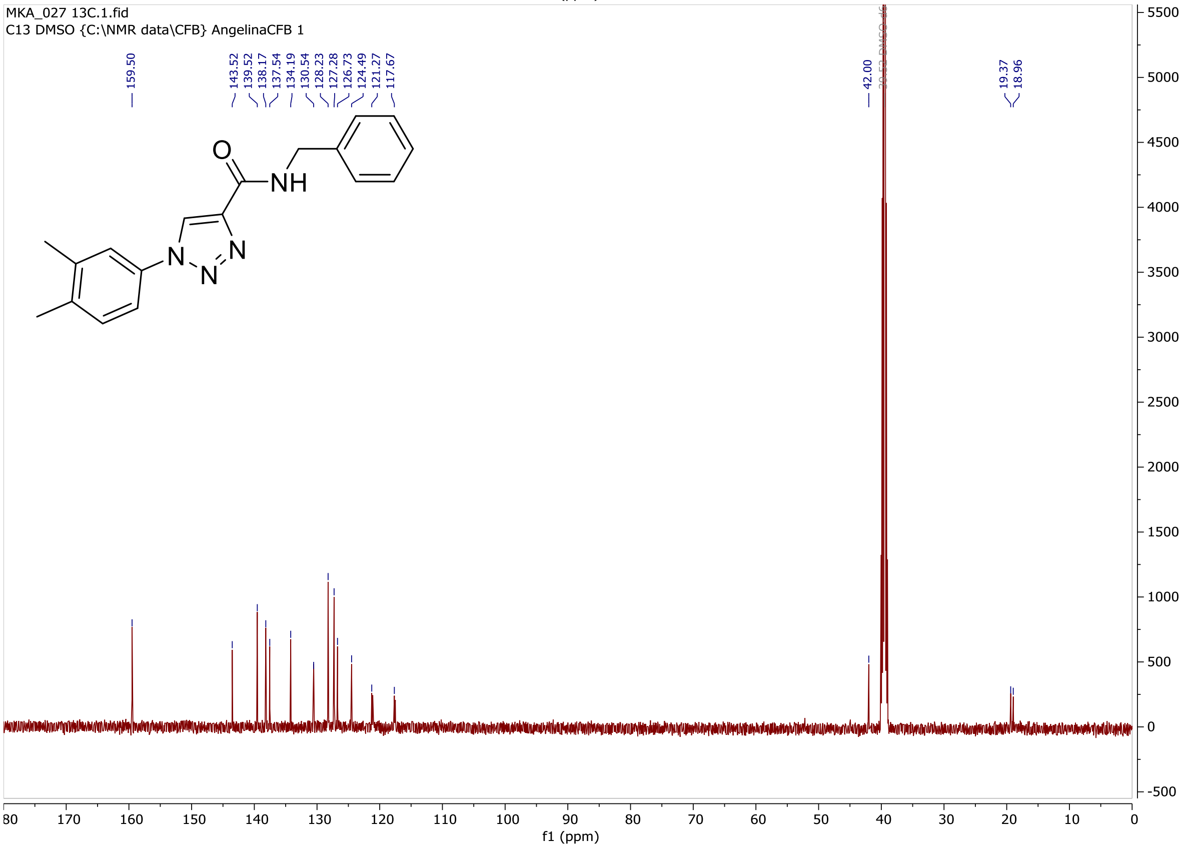

^{1}H and ^{13}C NMR spectra of compound 6w (MKA122)

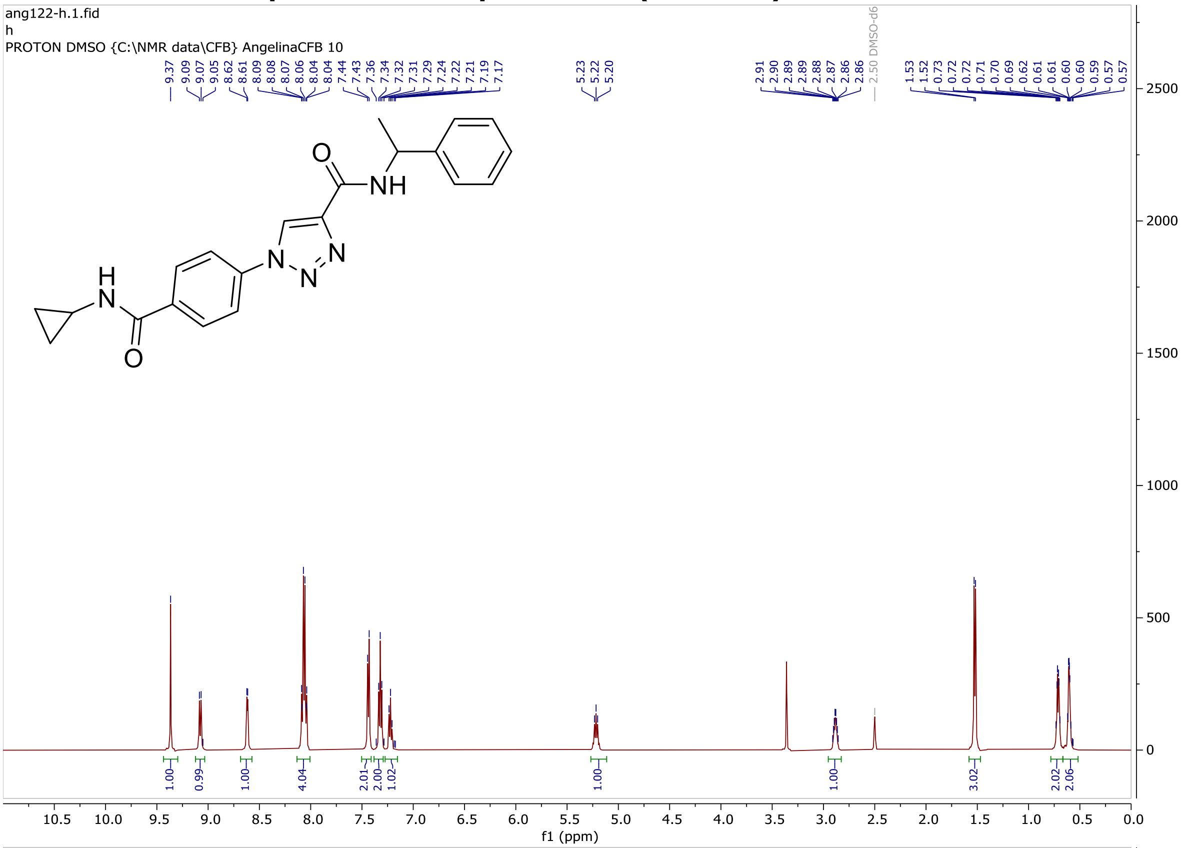

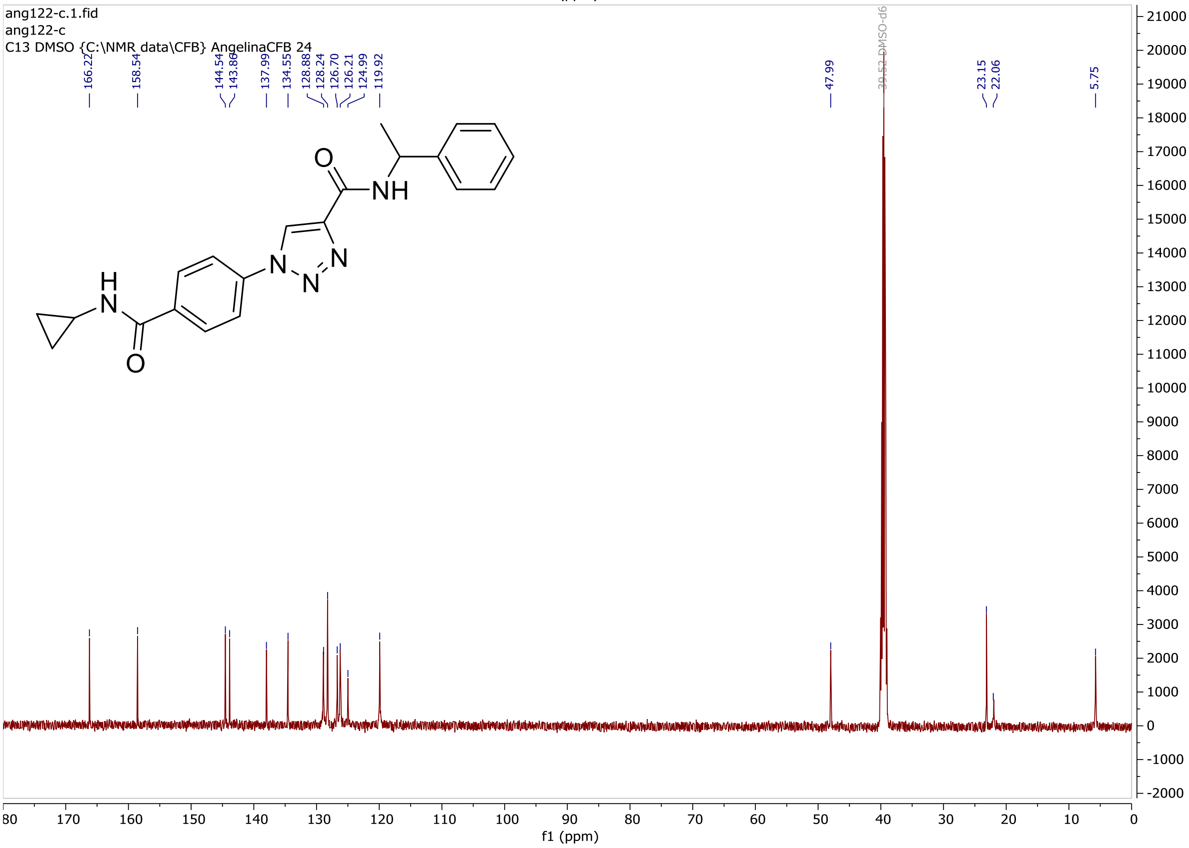

^{1}H and ^{13}C NMR spectra of compound 6x (MKA019)

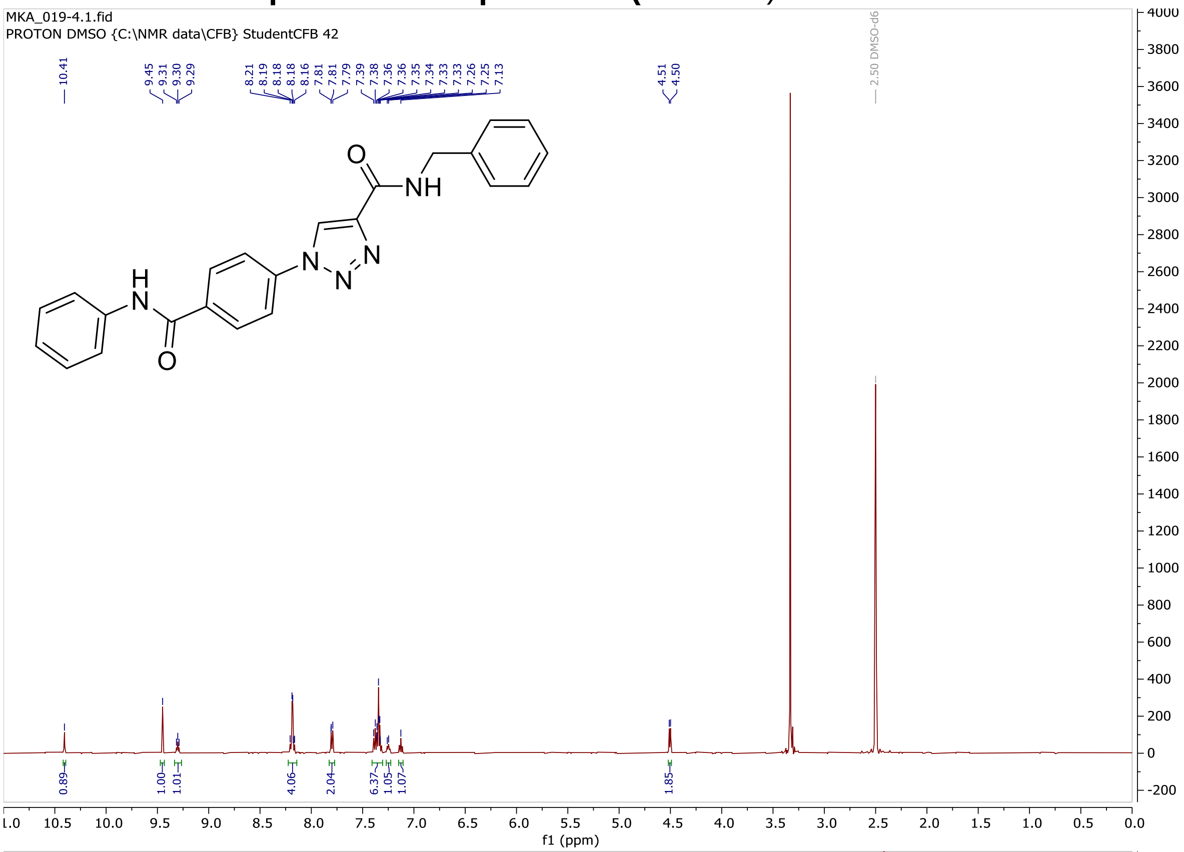

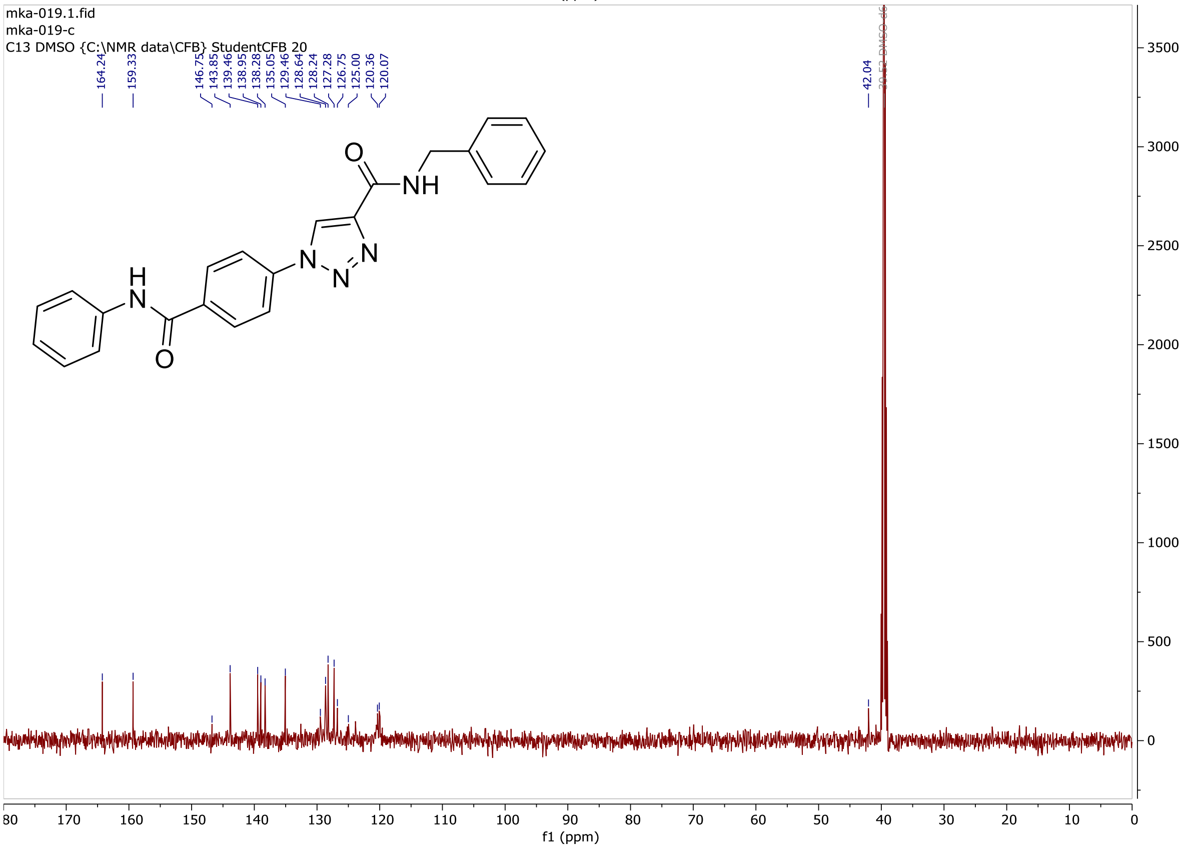

^{1}H and ^{13}C NMR spectra of compound 6y (MKA031)

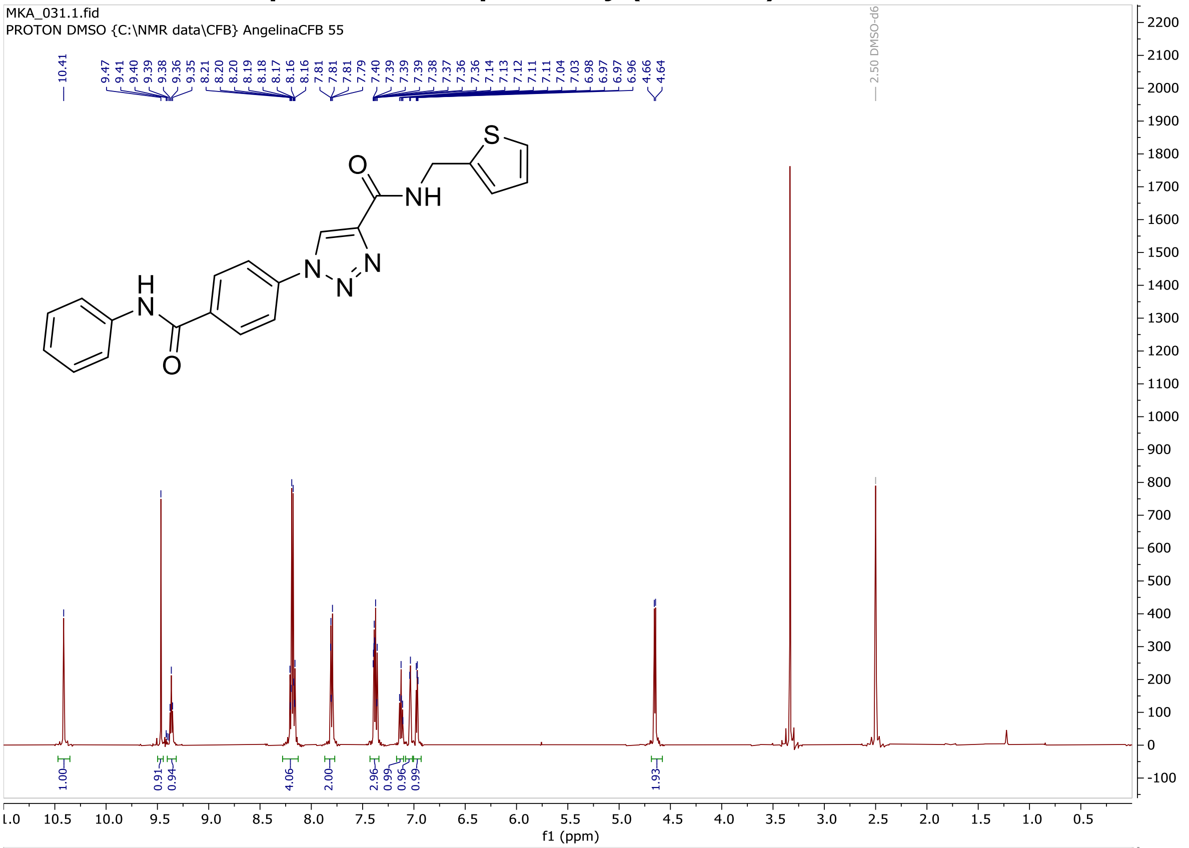

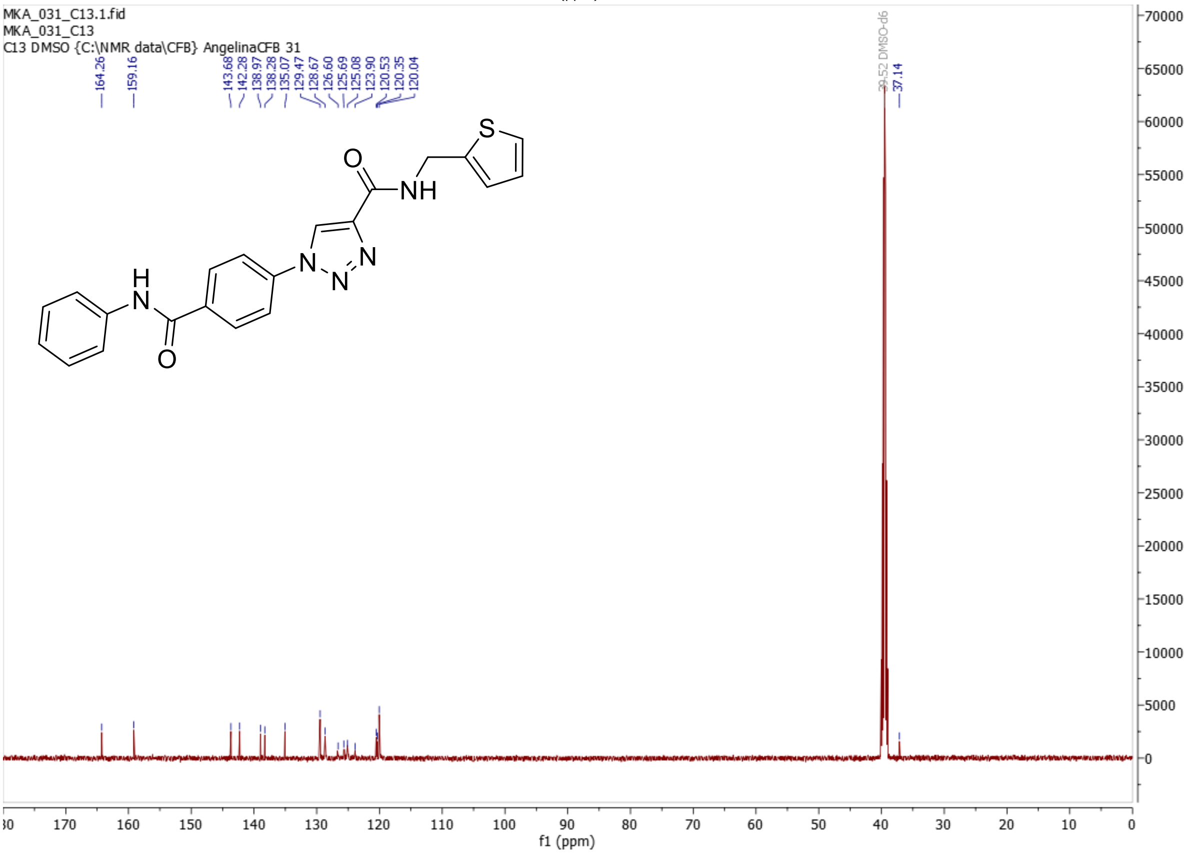

3. Copies of HPLC data of all compounds

HPLC data of 6a

University of Groningen   Dept. Pharmaceutical Biology   Groningen Research Institute of Pharmacy

Data Report

HPLC

Sample ID	: ANG_105	Tray	: 1
Date	: 23-2-2023	Vial	: 2
Data File Name	: ANG_105__003.lcd	Inj. Volume	: 10
Method File Name	: gradient_1mL_10-90%B_30C_15min_HPLC2-Print.lcm	Flow	: 1.0 mL/min
Report File Name	: MIF inhibitors	Temperature	: 30°C
Batch File	: 23022023 MIF-AIF inhibitors-print.lcb	Detection	: UV 254 nm
Column	: Kinetex 5um EVO C18 100A, 150x4.6mm		
Mobile phase A	: Water		
Mobile phase B	: Acetonitrile		
Gradient	: Acetonitrile 10-90%, 15 min		
Column nr	: 00F-4633-E0		
Serial nr	: 745950-4		
Supplier	: Phenomenex		

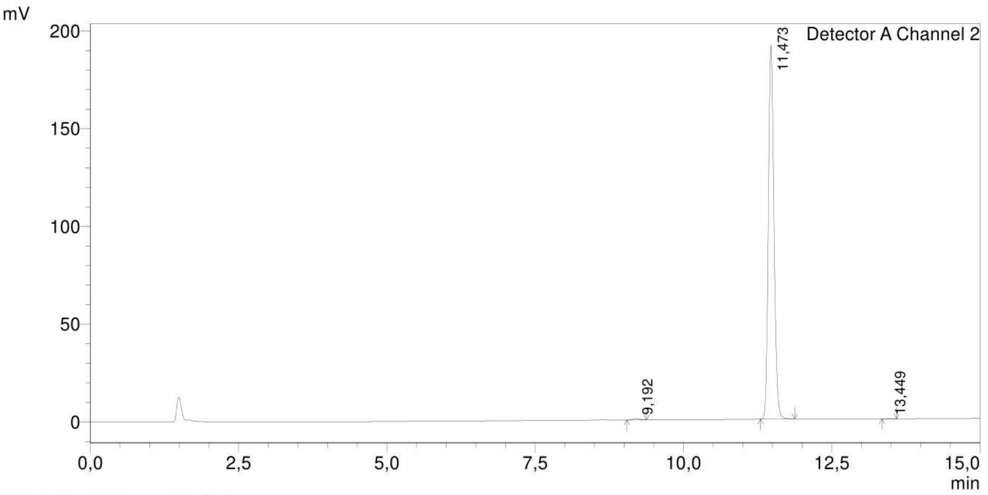

1 Detector A Channel 2/254nm

PeakTable

Detector A Channel 2 254nm

Peak#	Ret. Time	Area	Area %
1	9,192	5383	0,422
2	11,473	1267669	99,489
3	13,449	1128	0,088
Total		1274180	100,000

24-2-2023 14:54:13

HPLC data of 6b

University of Groningen Dept. Pharmaceutical Biology Groningen Research Institute of Pharmacy

Data Report

HPLC

Sample ID	: ANG_103	Tray	: 1
Date	: 23-2-2023	Vial	: 1
Data File Name	: ANG_103__001.lcd	Inj. Volume	: 10
Method File Name	: gradient_1mL_10-90%B_30C_15min_HPLC2-Print.lcm	Flow	: 1.0 mL/min
Report File Name	: MIF inhibitors	Temperature	: 30°C
Batch File	: 23022023 MIF-AIF inhibitors-print.lcb	Detection	: UV 254 nm
Column	: Kinetex 5um EVO C18 100A, 150x4.6mm		
Mobile phase A	: Water		
Mobile phase B	: Acetonitrile		
Gradient	: Acetonitrile 10-90%, 15 min		
Column nr	: 00F-4633-E0		
Serial nr	: 745950-4		
Supplier	: Phenomenex		

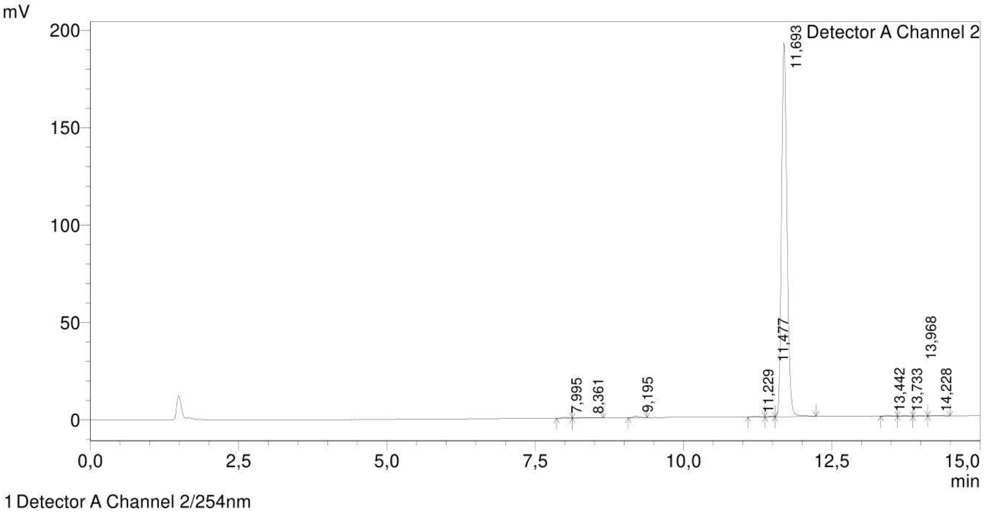

1 Detector A Channel 2/254nm

PeakTable

Detector A Channel 2 254nm

Peak#	Ret. Time	Area	Area %
1	7,995	3278	0,264
2	8,361	4301	0,347
3	9,195	5234	0,422
4	11,229	1323	0,107
5	11,477	2216	0,179
6	11,693	1214247	97,903
7	13,442	3167	0,255
8	13,733	1955	0,158
9	13,968	1842	0,148
10	14,228	2688	0,217
Total		1240251	100,000

24-2-2023 14:53:46

HPLC data of 6c

University of Groningen Dept. Pharmaceutical Biology Groningen Research Institute of Pharmacy

Data Report

HPLC

Sample ID	: ANG_109	Tray	: 1
Date	: 23-2-2023	Vial	: 9
Data File Name	: ANG_109__017.lcd	Inj. Volume	: 10
Method File Name	: gradient_1mL_10-90%B_30C_15min_HPLC2-Print.lcm	Flow	: 1.0 mL/min
Report File Name	: MIF inhibitors	Temperature	: 30°C
Batch File	: 23022023 MIF-AIF inhibitors-print.lcb	Detection	: UV 254 nm
Column	: Kinetex 5um EVO C18 100A, 150x4.6mm		
Mobile phase A	: Water		
Mobile phase B	: Acetonitrile		
Gradient	: Acetonitrile 10-90%, 15 min		
Column nr	: 00F-4633-E0		
Serial nr	: 745950-4		
Supplier	: Phenomenex		

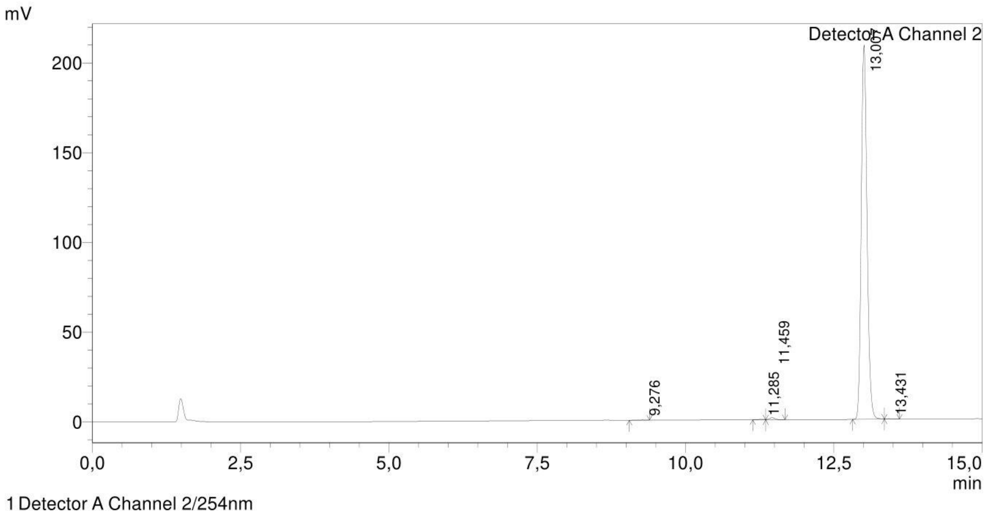

1 Detector A Channel 2/254nm

PeakTable

Detector A Channel 2 254nm

Peak#	Ret. Time	Area	Area %
1	9,276	1386	0,099
2	11,285	1588	0,114
3	11,459	7696	0,550
4	13,007	1386098	99,090
5	13,431	2061	0,147
Total		1398828	100,000

24-2-2023 14:57:59

HPLC data of 6d

University of Groningen Dept. Pharmaceutical Biology Groningen Research Institute of Pharmacy

Data Report

HPLC

Sample ID	: ANG_108	Tray	: 1
Date	: 23-2-2023	Vial	: 5
Data File Name	: ANG_108__009.lcd	Inj. Volume	: 10
Method File Name	: gradient_1mL_10-90%B_30C_15min_HPLC2-Print.lcm	Flow	: 1.0 mL/min
Report File Name	: MIF inhibitors	Temperature	: 30°C
Batch File	: 23022023 MIF-AIF inhibitors-print.lcb	Detection	: UV 254 nm
Column	: Kinetex 5um EVO C18 100A, 150x4.6mm		
Mobile phase A	: Water		
Mobile phase B	: Acetonitrile		
Gradient	: Acetonitrile 10-90%, 15 min		
Column nr	: 00F-4633-E0		
Serial nr	: 745950-4		
Supplier	: Phenomenex		

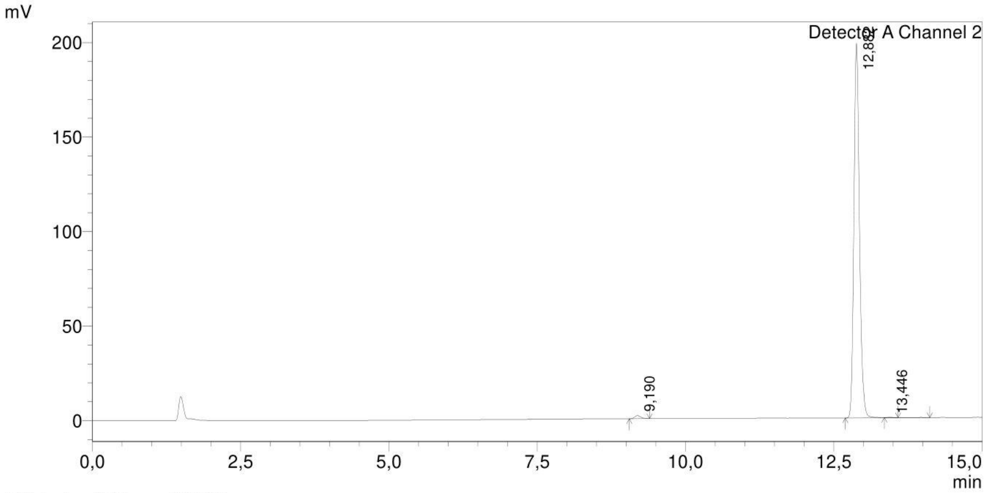

1 Detector A Channel 2/254nm

PeakTable

Detector A Channel 2 254nm

Peak#	Ret. Time	Area	Area %
1	9,190	12079	0,914
2	12,882	1308785	99,006
3	13,446	1060	0,080
Total		1321925	100,000

24-2-2023 14:55:04

HPLC data of 6e

University of Groningen Dept. Pharmaceutical Biology Groningen Research Institute of Pharmacy

Data Report

HPLC

Sample ID	: ANG_125	Tray	: 1
Date	: 23-2-2023	Vial	: 6
Data File Name	: ANG_125__011.lcd	Inj. Volume	: 10
Method File Name	: gradient_1mL_10-90%B_30C_15min_HPLC2-Print.lcm	Flow	: 1.0 mL/min
Report File Name	: MIF inhibitors	Temperature	: 30°C
Batch File	: 23022023 MIF-AIF inhibitors-print.lcb	Detection	: UV 254 nm
Column	: Kinetex 5um EVO C18 100A, 150x4.6mm		
Mobile phase A	: Water		
Mobile phase B	: Acetonitrile		
Gradient	: Acetonitrile 10-90%, 15 min		
Column nr	: 00F-4633-E0		
Serial nr	: 745950-4		
Supplier	: Phenomenex		

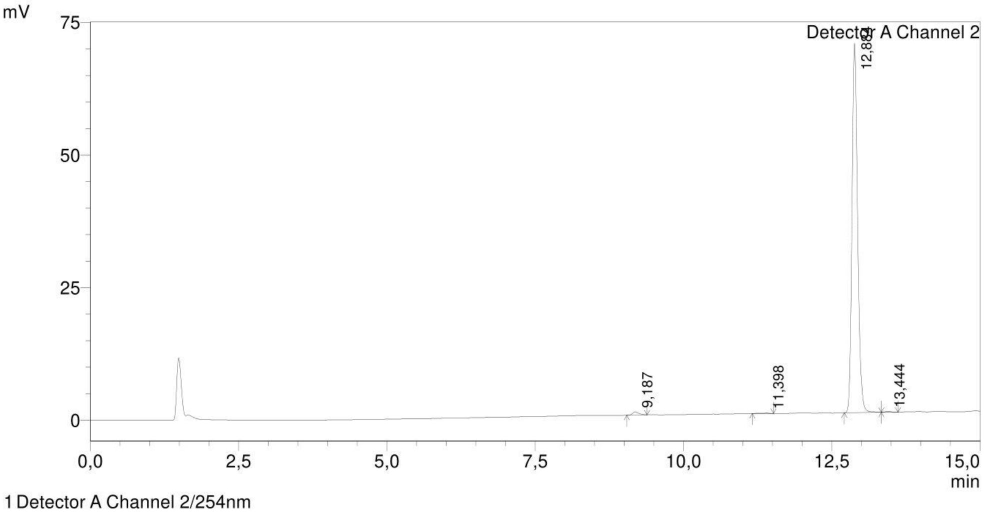

1 Detector A Channel 2/254nm

PeakTable

Detector A Channel 2 254nm

Peak#	Ret. Time	Area	Area %
1	9,187	4665	0,992
2	11,398	1226	0,261
3	12,884	463331	98,486
4	13,444	1230	0,261
Total		470452	100,000

24-2-2023 14:55:24

HPLC data of 6f

University of Groningen Dept. Pharmaceutical Biology Groningen Research Institute of Pharmacy

Data Report

HPLC

Sample ID	: ANG_102	Tray	: 1
Date	: 24-2-2023	Vial	: 18
Data File Name	: ANG_102__035.lcd	Inj. Volume	: 10
Method File Name	: gradient_1mL_10-90%B_30C_15min_HPLC2-Print.lcm	Flow	: 1.0 mL/min
Report File Name	: MIF inhibitors	Temperature	: 30°C
Batch File	: 23022023 MIF-AIF inhibitors-print.lcb	Detection	: UV 254 nm
Column	: Kinetex 5um EVO C18 100A, 150x4.6mm		
Mobile phase A	: Water		
Mobile phase B	: Acetonitrile		
Gradient	: Acetonitrile 10-90%, 15 min		
Column nr	: 00F-4633-E0		
Serial nr	: 745950-4		
Supplier	: Phenomenex		

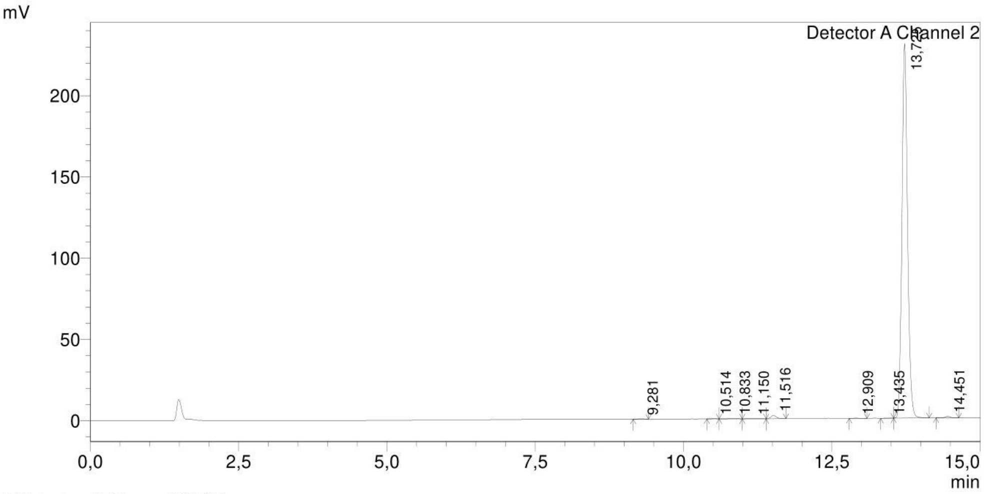

1 Detector A Channel 2/254nm

PeakTable

Detector A Channel 2 254nm

Peak#	Ret. Time	Area	Area %
1	9,281	1058	0,069
2	10,514	1025	0,067
3	10,833	3243	0,210
4	11,150	1824	0,118
5	11,516	13442	0,872
6	12,909	1028	0,067
7	13,435	1048	0,068
8	13,725	1512210	98,098
9	14,451	6656	0,432
Total		1541534	100,000

24-2-2023 14:57:37

HPLC data of 6g

University of Groningen Dept. Pharmaceutical Biology Groningen Research Institute of Pharmacy

Data Report

HPLC

Sample ID	: ANG_095	Tray	: 1
Date	: 23-2-2023	Vial	: 14
Data File Name	: ANG_095__027.lcd	Inj. Volume	: 10
Method File Name	: gradient_1mL_10-90%B_30C_15min_HPLC2-Print.lcm	Flow	: 1.0 mL/min
Report File Name	: MIF inhibitors	Temperature	: 30°C
Batch File	: 23022023 MIF-AIF inhibitors-print.lcb	Detection	: UV 254 nm
Column	: Kinetex 5um EVO C18 100A, 150x4.6mm		
Mobile phase A	: Water		
Mobile phase B	: Acetonitrile		
Gradient	: Acetonitrile 10-90%, 15 min		
Column nr	: 00F-4633-E0		
Serial nr	: 745950-4		
Supplier	: Phenomenex		

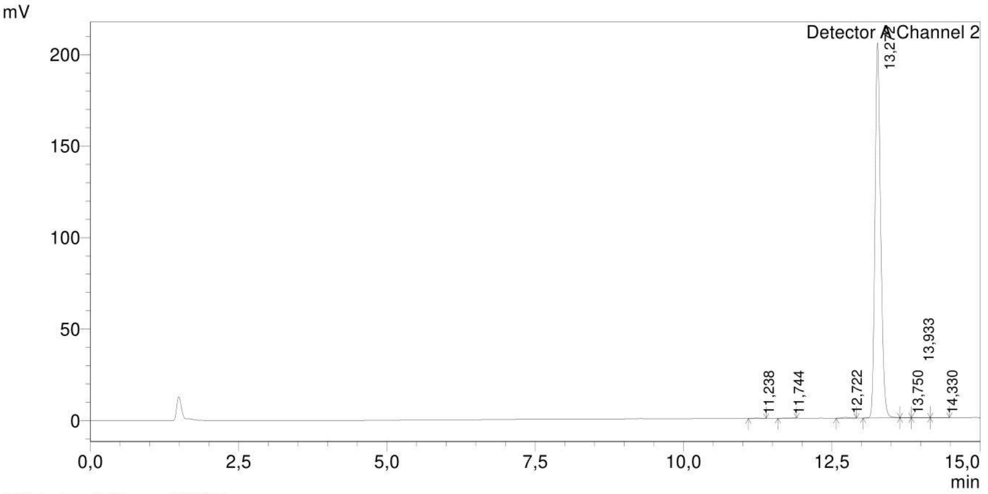

1 Detector A Channel 2/254nm

PeakTable

Detector A Channel 2 254nm

Peak#	Ret. Time	Area	Area %
1	11,238	1399	0,102
2	11,744	1847	0,135
3	12,722	3580	0,261
4	13,272	1359994	99,181
5	13,750	1689	0,123
6	13,933	1430	0,104
7	14,330	1287	0,094
Total		1371226	100,000

24-2-2023 14:56:50

HPLC data of 6h

University of Groningen Dept. Pharmaceutical Biology Groningen Research Institute of Pharmacy

Data Report

HPLC

Sample ID	: MKA_030	Tray	: 1
Date	: 24-2-2023	Vial	: 20
Data File Name	: MKA_030__039.lcd	Inj. Volume	: 10
Method File Name	: gradient_1mL_10-90%B_30C_15min_HPLC2-Print.lcm	Flow	: 1.0 mL/min
Report File Name	: MIF inhibitors	Temperature	: 30°C
Batch File	: 23022023 MIF-AIF inhibitors-print.lcb	Detection	: UV 254 nm
Column	: Kinetex 5um EVO C18 100A, 150x4.6mm		
Mobile phase A	: Water		
Mobile phase B	: Acetonitrile		
Gradient	: Acetonitrile 10-90%, 15 min		
Column nr	: 00F-4633-E0		
Serial nr	: 745950-4		
Supplier	: Phenomenex		

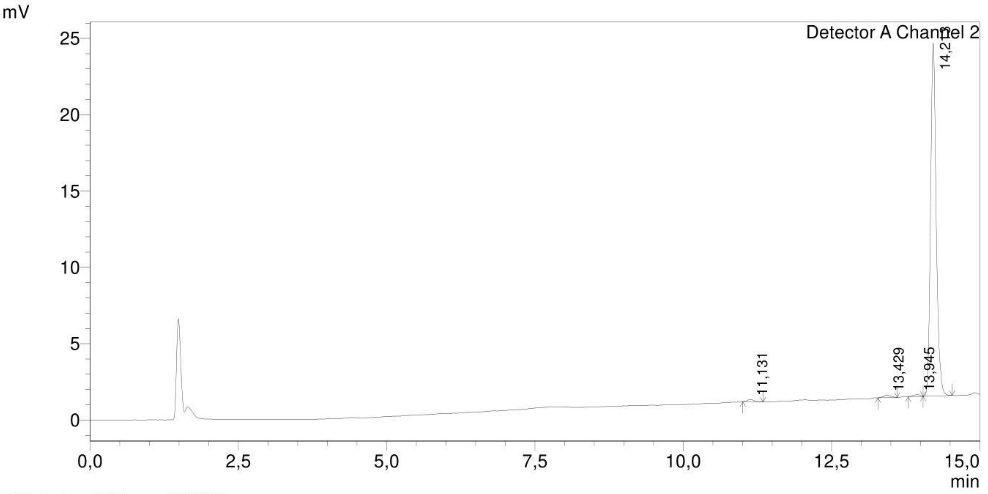

1 Detector A Channel 2/254nm

PeakTable

Detector A Channel 2 254nm

Peak#	Ret. Time	Area	Area %
1	11,131	1287	0,816
2	13,429	1512	0,959
3	13,945	1008	0,639
4	14,213	153890	97,586
Total		157697	100,000

24-2-2023 15:02:34

HPLC data of 6i

Data Report

HPLC

Sample ID	: MKA_044	Tray	: 1
Date	: 24-2-2023	Vial	: 22
Data File Name	: MKA_044__004.lcd	Inj. Volume	: 10
Method File Name	: gradient_1mL_10-90%B_30C_15min_HPLC2.lcm	Flow	: 1.0 mL/min
Report File Name	: MIF inhibitors	Temperature	: 30°C
Batch File	: 23022023 MIF-AIF inhibitors.lcb	Detection	: UV 254 nm
Column	: Kinetex 5um EVO C18 100A, 150x4.6mm		
Mobile phase A	: Water		
Mobile phase B	: Acetonitrile		
Gradient	: Acetonitrile 10-90%, 15 min		
Column nr	: 00F-4633-E0		
Serial nr	: 745950-4		
Supplier	: Phenomenex		

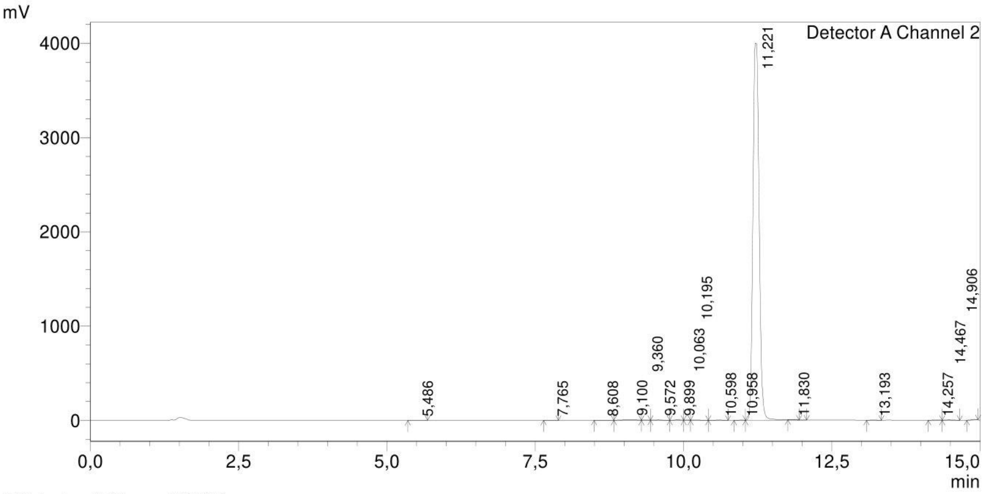

1 Detector A Channel 2/254nm

PeakTable

Detector A Channel 2 254nm

Peak#	Ret. Time	Area	Area %
1	5,486	7716	0,026
2	7,765	5070	0,017
3	8,608	14545	0,049
4	9,100	117313	0,395
5	9,360	19167	0,064
6	9,572	38204	0,129
7	9,899	35433	0,119
8	10,063	14085	0,047
9	10,195	26105	0,088
10	10,598	13864	0,047
11	10,958	4316	0,015
12	11,221	29345510	98,731
13	11,830	7817	0,026
14	13,193	4938	0,017
15	14,257	11196	0,038
16	14,467	24622	0,083
17	14,906	32681	0,110
Total		29722583	100,000

27-2-2023 12:58:03

HPLC data of 6j

Data Report

HPLC

Sample ID	: MKA_084	Tray	: 1
Date	: 23-2-2023	Vial	: 11
Data File Name	: MKA_084__021.lcd	Inj. Volume	: 10
Method File Name	: gradient_1mL_10-90%B_30C_15min_HPLC2-Print.lcm	Flow	: 1.0 mL/min
Report File Name	: MIF inhibitors	Temperature	: 30°C
Batch File	: 23022023 MIF-AIF inhibitors-print.lcb	Detection	: UV 254 nm
Column	: Kinetex 5um EVO C18 100A, 150x4.6mm		
Mobile phase A	: Water		
Mobile phase B	: Acetonitrile		
Gradient	: Acetonitrile 10-90%, 15 min		
Column nr	: 00F-4633-E0		
Serial nr	: 745950-4		
Supplier	: Phenomenex		

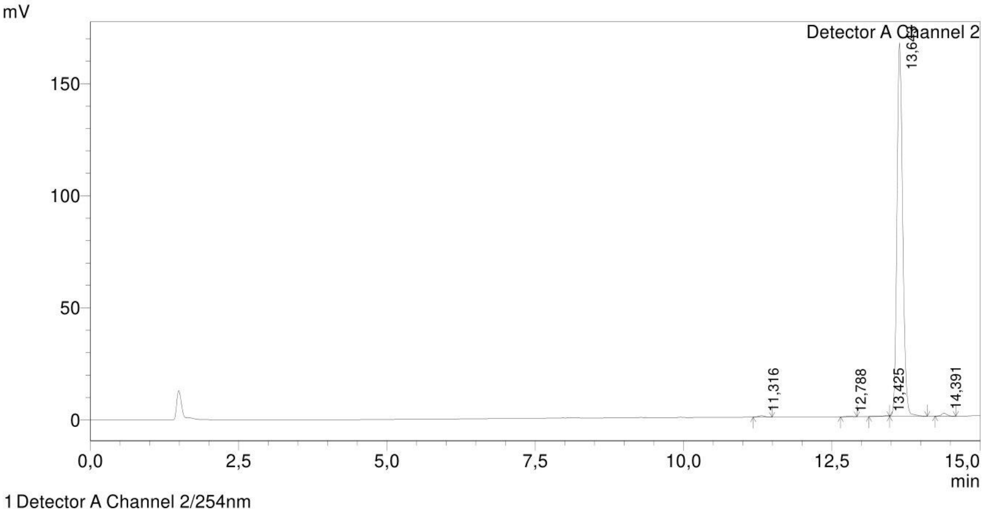

1 Detector A Channel 2/254nm

PeakTable

Detector A Channel 2 254nm

Peak#	Ret. Time	Area	Area %
1	11,316	5124	0,460
2	12,788	1316	0,118
3	13,425	3352	0,301
4	13,643	1094622	98,357
5	14,391	8497	0,764
Total		1112912	100,000

24-2-2023 15:00:52

HPLC data of 6k

Data Report

HPLC

Sample ID	: MKA_085	Tray	: 1
Date	: 23-2-2023	Vial	: 12
Data File Name	: MKA_085__023.lcd	Inj. Volume	: 10
Method File Name	: gradient_1mL_10-90%B_30C_15min_HPLC2-Print.lcm	Flow	: 1.0 mL/min
Report File Name	: MIF inhibitors	Temperature	: 30°C
Batch File	: 23022023 MIF-AIF inhibitors-print.lcb	Detection	: UV 254 nm
Column	: Kinetex 5um EVO C18 100A, 150x4.6mm		
Mobile phase A	: Water		
Mobile phase B	: Acetonitrile		
Gradient	: Acetonitrile 10-90%, 15 min		
Column nr	: 00F-4633-E0		
Serial nr	: 745950-4		
Supplier	: Phenomenex		

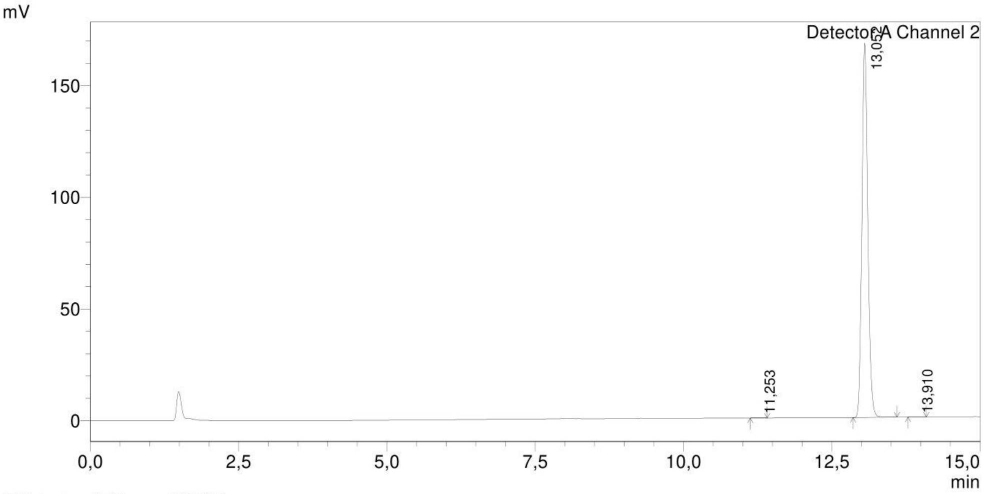

1 Detector A Channel 2/254nm

PeakTable

Detector A Channel 2 254nm

Peak#	Ret. Time	Area	Area %
1	11,253	1409	0,122
2	13,052	1153042	99,748
3	13,910	1507	0,130
Total		1155958	100,000

24-2-2023 15:01:07

HPLC data of 6l

University of Groningen   Dept. Pharmaceutical Biology   Groningen Research Institute of Pharmacy

Data Report

HPLC

Sample ID	: MKA_038	Tray	: 1
Date	: 24-2-2023	Vial	: 27
Data File Name	: MKA_038__001.lcd	Inj. Volume	: 10
Method File Name	: gradient_1mL_10-90%B_30C_15min_HPLC2.lcm	Flow	: 1.0 mL/min
Report File Name	: MIF inhibitors	Temperature	: 30°C
Batch File	: 23022023 MIF-AIF inhibitors.lcb	Detection	: UV 254 nm
Column	: Kinetex 5um EVO C18 100A, 150x4.6mm		
Mobile phase A	: Water		
Mobile phase B	: Acetonitrile		
Gradient	: Acetonitrile 10-90%, 15 min		
Column nr	: 00F-4633-E0		
Serial nr	: 745950-4		
Supplier	: Phenomenex		

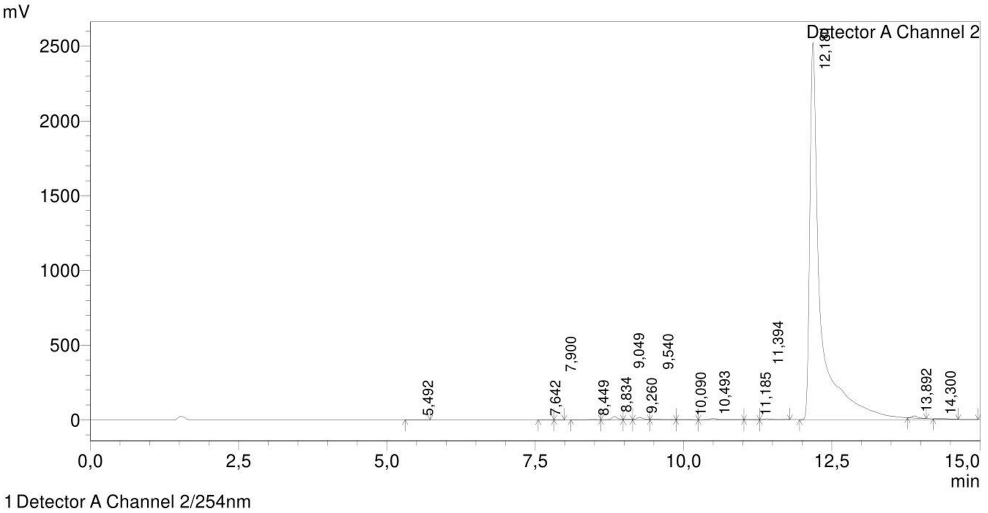

1 Detector A Channel 2/254nm

PeakTable

Detector A Channel 2 254nm

Peak#	Ret. Time	Area	Area %
1	5,492	5035	0,015
2	7,642	1118	0,003
3	7,900	2197	0,007
4	8,449	15645	0,047
5	8,834	151980	0,459
6	9,049	24454	0,074
7	9,260	126281	0,381
8	9,540	49227	0,149
9	10,090	12222	0,037
10	10,493	76623	0,231
11	11,185	14770	0,045
12	11,394	55120	0,166
13	12,181	32449967	97,950
14	13,892	100921	0,305
15	14,300	43414	0,131
Total		33128973	100,000

25-2-2023 00:02:07

HPLC data of 6m

University of Groningen Dept. Pharmaceutical Biology Groningen Research Institute of Pharmacy

Data Report

HPLC

Sample ID	: MKA_029	Tray	: 1
Date	: 24-2-2023	Vial	: 23
Data File Name	: MKA_029__045.lcd	Inj. Volume	: 10
Method File Name	: gradient_1mL_10-90%B_30C_15min_HPLC2-Print.lcm	Flow	: 1.0 mL/min
Report File Name	: MIF inhibitors	Temperature	: 30°C
Batch File	: 23022023 MIF-AIF inhibitors-print.lcb	Detection	: UV 254 nm
Column	: Kinetex 5um EVO C18 100A, 150x4.6mm		
Mobile phase A	: Water		
Mobile phase B	: Acetonitrile		
Gradient	: Acetonitrile 10-90%, 15 min		
Column nr	: 00F-4633-E0		
Serial nr	: 745950-4		
Supplier	: Phenomenex		

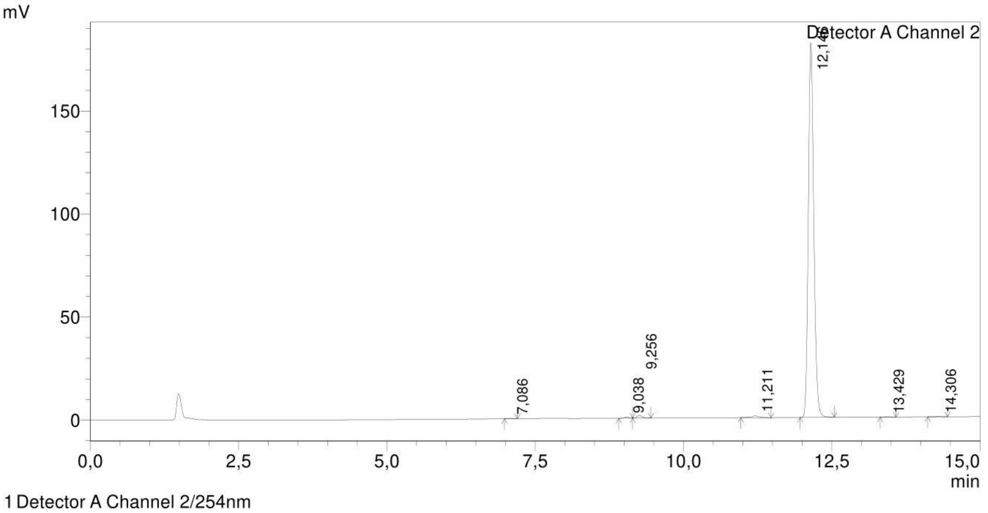

1 Detector A Channel 2/254nm

PeakTable

Detector A Channel 2 254nm

Peak#	Ret. Time	Area	Area %
1	7,086	1224	0,102
2	9,038	3036	0,252
3	9,256	8346	0,693
4	11,211	7569	0,628
5	12,146	1181692	98,118
6	13,429	1121	0,093
7	14,306	1367	0,114
Total		1204355	100,000

24-2-2023 15:02:07

HPLC data of 6n

Data Report

HPLC

Sample ID	: ANG_106	Tray	: 1
Date	: 23-2-2023	Vial	: 3
Data File Name	: ANG_106__005.lcd	Inj. Volume	: 10
Method File Name	: gradient_1mL_10-90%B_30C_15min_HPLC2-Print.lcm	Flow	: 1.0 mL/min
Report File Name	: MIF inhibitors	Temperature	: 30°C
Batch File	: 23022023 MIF-AIF inhibitors-print.lcb	Detection	: UV 254 nm
Column	: Kinetex 5um EVO C18 100A, 150x4.6mm		
Mobile phase A	: Water		
Mobile phase B	: Acetonitrile		
Gradient	: Acetonitrile 10-90%, 15 min		
Column nr	: 00F-4633-E0		
Serial nr	: 745950-4		
Supplier	: Phenomenex		

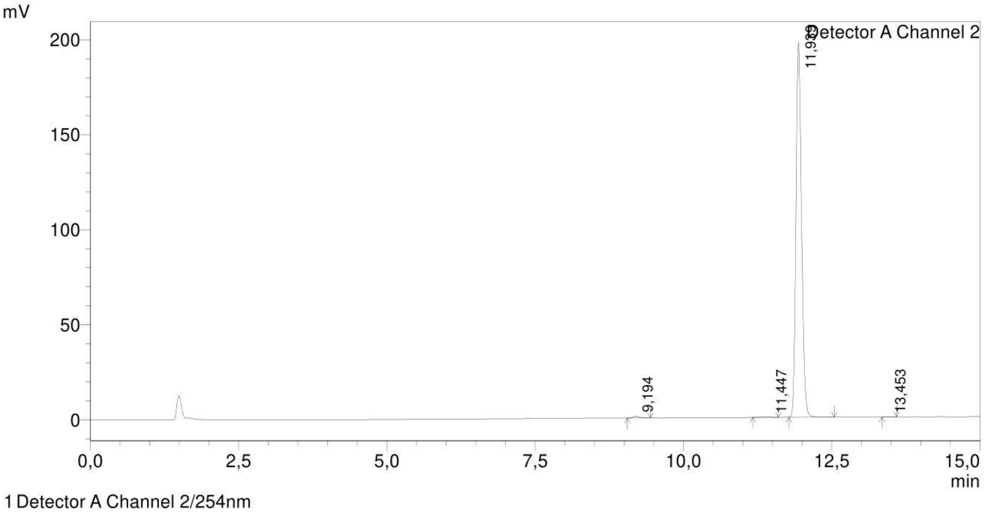

1 Detector A Channel 2/254nm

PeakTable

Detector A Channel 2 254nm

Peak#	Ret. Time	Area	Area %
1	9,194	7399	0,574
2	11,447	1968	0,153
3	11,939	1277965	99,186
4	13,453	1123	0,087
Total		1288455	100,000

24-2-2023 14:54:43

HPLC data of 6o

University of Groningen Dept. Pharmaceutical Biology Groningen Research Institute of Pharmacy

Data Report

HPLC

Sample ID	: MKA_097	Tray	: 1
Date	: 24-2-2023	Vial	: 28
Data File Name	: MKA_097__055.lcd	Inj. Volume	: 10
Method File Name	: gradient_1mL_10-90%B_30C_15min_HPLC2-Print.lcm	Flow	: 1.0 mL/min
Report File Name	: MIF inhibitors	Temperature	: 30°C
Batch File	: 23022023 MIF-AIF inhibitors-print.lcb	Detection	: UV 254 nm
Column	: Kinetex 5um EVO C18 100A, 150x4.6mm		
Mobile phase A	: Water		
Mobile phase B	: Acetonitrile		
Gradient	: Acetonitrile 10-90%, 15 min		
Column nr	: 00F-4633-E0		
Serial nr	: 745950-4		
Supplier	: Phenomenex		

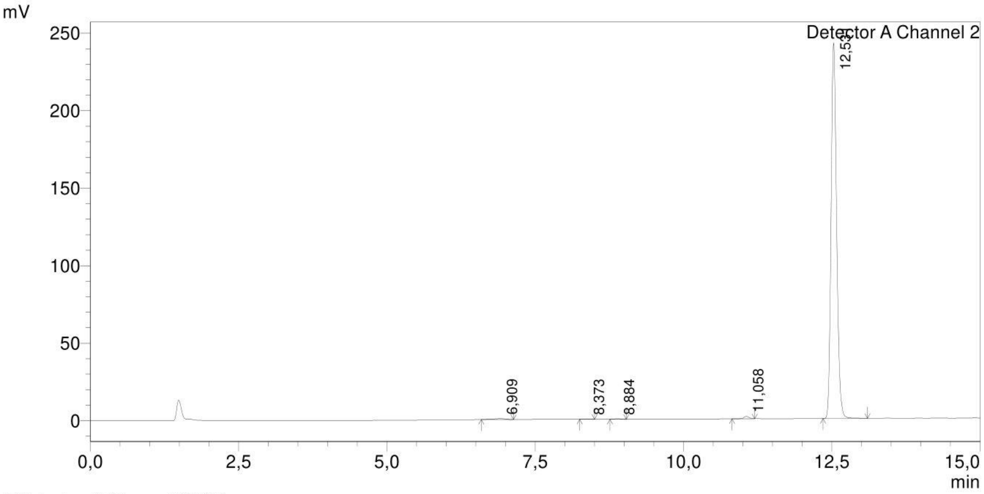

1 Detector A Channel 2/254nm

PeakTable

Detector A Channel 2 254nm

Peak#	Ret. Time	Area	Area %
1	6,909	8519	0,529
2	8,373	1537	0,095
3	8,884	2090	0,130
4	11,058	10886	0,676
5	12,531	1588363	98,571
Total		1611396	100,000

24-2-2023 15:04:09

HPLC data of 6p

Data Report

HPLC

Sample ID	: ANG_098	Tray	: 1
Date	: 23-2-2023	Vial	: 15
Data File Name	: ANG_098__029.lcd	Inj. Volume	: 10
Method File Name	: gradient_1mL_10-90%B_30C_15min_HPLC2-Print.lcm	Flow	: 1.0 mL/min
Report File Name	: MIF inhibitors	Temperature	: 30°C
Batch File	: 23022023 MIF-AIF inhibitors-print.lcb	Detection	: UV 254 nm
Column	: Kinetex 5um EVO C18 100A, 150x4.6mm		
Mobile phase A	: Water		
Mobile phase B	: Acetonitrile		
Gradient	: Acetonitrile 10-90%, 15 min		
Column nr	: 00F-4633-E0		
Serial nr	: 745950-4		
Supplier	: Phenomenex		

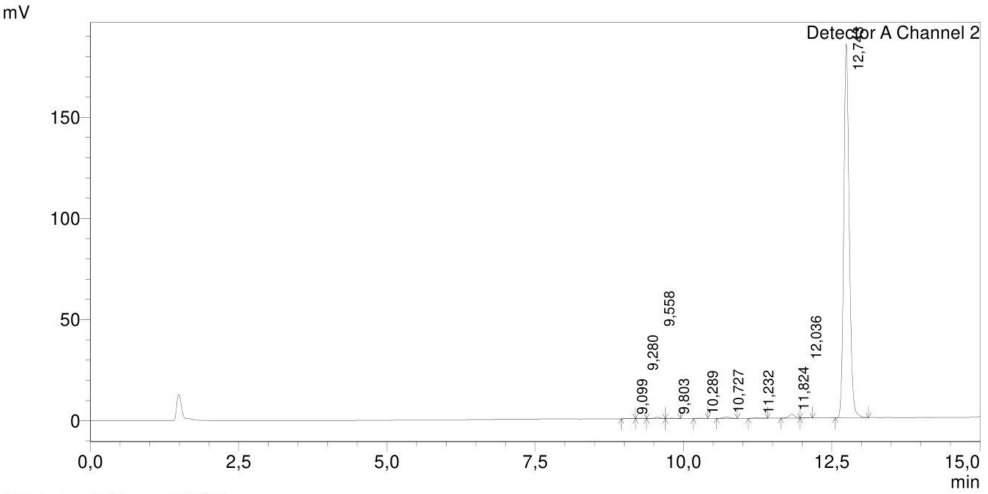

1 Detector A Channel 2/254nm

PeakTable

Detector A Channel 2 254nm

Peak#	Ret. Time	Area	Area %
1	9,099	1597	0,131
2	9,280	1384	0,113
3	9,558	5189	0,424
4	9,803	1744	0,143
5	10,289	1279	0,105
6	10,727	4667	0,382
7	11,232	1258	0,103
8	11,824	13224	1,081
9	12,036	1564	0,128
10	12,743	1191361	97,392
Total		1223267	100,000

24-2-2023 14:57:18

HPLC data of 6q

University of Groningen Dept. Pharmaceutical Biology Groningen Research Institute of Pharmacy

Data Report

HPLC

Sample ID	: MKA_004	Tray	: 1
Date	: 23-2-2023	Vial	: 7
Data File Name	: MKA_004__013.lcd	Inj. Volume	: 10
Method File Name	: gradient_1mL_10-90%B_30C_15min_HPLC2-Print.lcm	Flow	: 1.0 mL/min
Report File Name	: MIF inhibitors	Temperature	: 30°C
Batch File	: 23022023 MIF-AIF inhibitors-print.lcb	Detection	: UV 254 nm
Column	: Kinetex 5um EVO C18 100A, 150x4.6mm		
Mobile phase A	: Water		
Mobile phase B	: Acetonitrile		
Gradient	: Acetonitrile 10-90%, 15 min		
Column nr	: 00F-4633-E0		
Serial nr	: 745950-4		
Supplier	: Phenomenex		

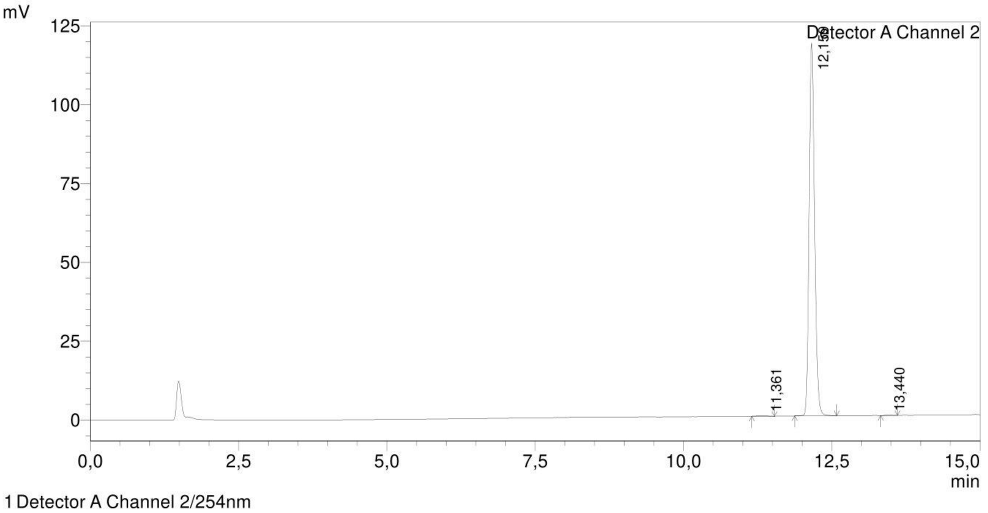

1 Detector A Channel 2/254nm

PeakTable

Detector A Channel 2 254nm

Peak#	Ret. Time	Area	Area %
1	11,361	1831	0,238
2	12,159	765674	99,597
3	13,440	1268	0,165
Total		768773	100,000

24-2-2023 14:55:48

HPLC data of 6r

University of Groningen Dept. Pharmaceutical Biology Groningen Research Institute of Pharmacy

Data Report

HPLC

Sample ID	: MKA_010	Tray	: 1
Date	: 23-2-2023	Vial	: 8
Data File Name	: MKA_010__015.lcd	Inj. Volume	: 10
Method File Name	: gradient_1mL_10-90%B_30C_15min_HPLC2-Print.lcm	Flow	: 1.0 mL/min
Report File Name	: MIF inhibitors	Temperature	: 30°C
Batch File	: 23022023 MIF-AIF inhibitors-print.lcb	Detection	: UV 254 nm
Column	: Kinetex 5um EVO C18 100A, 150x4.6mm		
Mobile phase A	: Water		
Mobile phase B	: Acetonitrile		
Gradient	: Acetonitrile 10-90%, 15 min		
Column nr	: 00F-4633-E0		
Serial nr	: 745950-4		
Supplier	: Phenomenex		

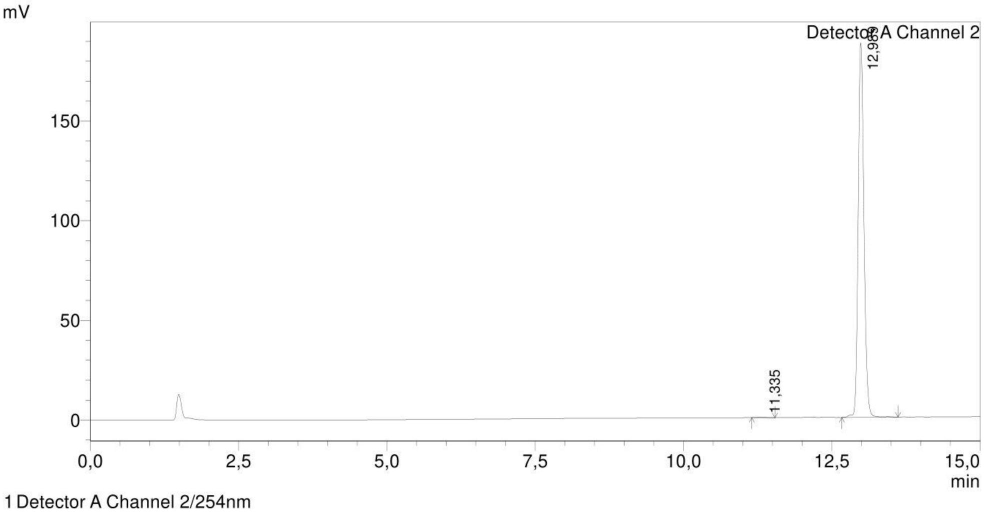

1 Detector A Channel 2/254nm

PeakTable

Detector A Channel 2 254nm

Peak#	Ret. Time	Area	Area %
1	11,335	2376	0,192
2	12,989	1237874	99,808
Total		1240249	100,000

24-2-2023 14:58:22

HPLC data of 6s

Data Report

HPLC

Sample ID	: MKA_048	Tray	: 1
Date	: 24-2-2023	Vial	: 16
Data File Name	: MKA_048__031.lcd	Inj. Volume	: 10
Method File Name	: gradient_1mL_10-90%B_30C_15min_HPLC2-Print.lcm	Flow	: 1.0 mL/min
Report File Name	: MIF inhibitors	Temperature	: 30°C
Batch File	: 23022023 MIF-AIF inhibitors-print.lcb	Detection	: UV 254 nm
Column	: Kinetex 5um EVO C18 100A, 150x4.6mm		
Mobile phase A	: Water		
Mobile phase B	: Acetonitrile		
Gradient	: Acetonitrile 10-90%, 15 min		
Column nr	: 00F-4633-E0		
Serial nr	: 745950-4		
Supplier	: Phenomenex		

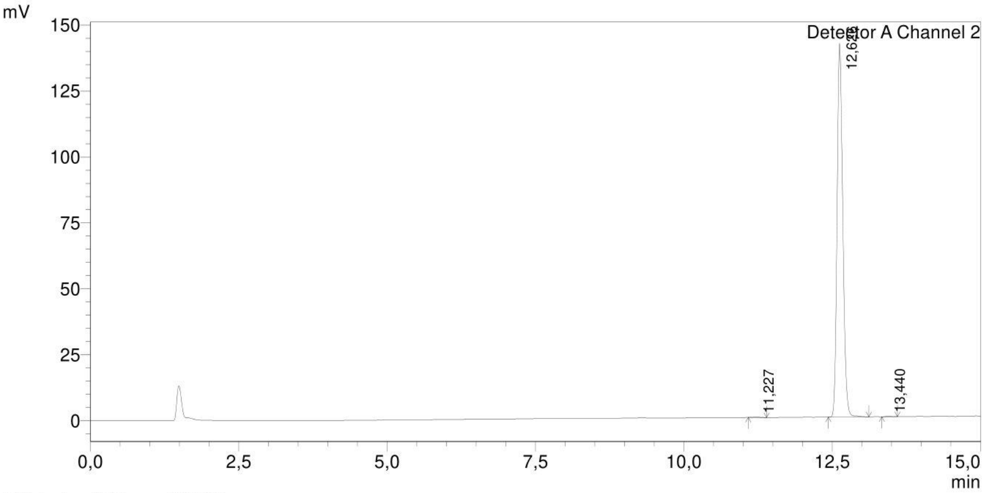

1 Detector A Channel 2/254nm

PeakTable

Detector A Channel 2 254nm

Peak#	Ret. Time	Area	Area %
1	11,227	1292	0,138
2	12,626	936453	99,740
3	13,440	1147	0,122
Total		938891	100,000

24-2-2023 15:00:37

HPLC data of 6t

Data Report

HPLC

Sample ID	: MKA_050	Tray	: 1
Date	: 24-2-2023	Vial	: 21
Data File Name	: MKA_050__041.lcd	Inj. Volume	: 10
Method File Name	: gradient_1mL_10-90%B_30C_15min_HPLC2-Print.lcm	Flow	: 1.0 mL/min
Report File Name	: MIF inhibitors	Temperature	: 30°C
Batch File	: 23022023 MIF-AIF inhibitors-print.lcb	Detection	: UV 254 nm
Column	: Kinetex 5um EVO C18 100A, 150x4.6mm		
Mobile phase A	: Water		
Mobile phase B	: Acetonitrile		
Gradient	: Acetonitrile 10-90%, 15 min		
Column nr	: 00F-4633-E0		
Serial nr	: 745950-4		
Supplier	: Phenomenex		

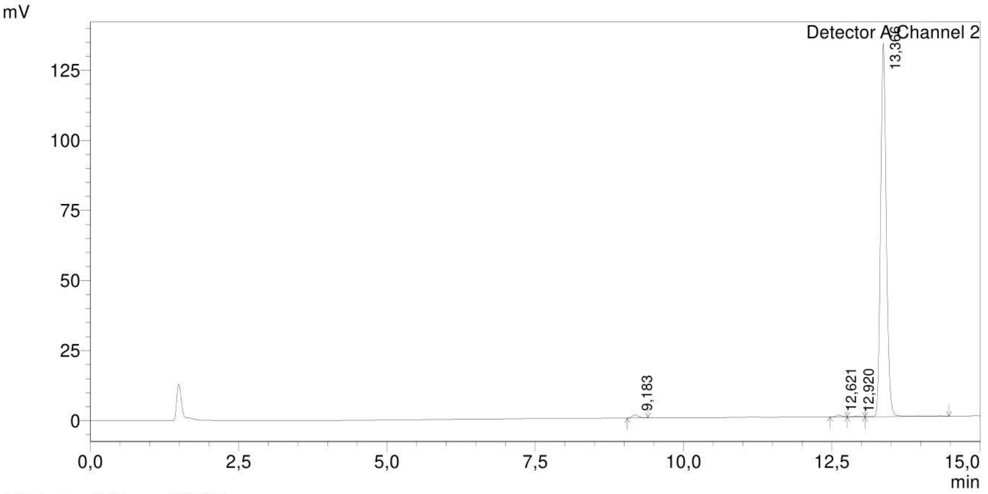

1 Detector A Channel 2/254nm

PeakTable

Detector A Channel 2 254nm

Peak#	Ret. Time	Area	Area %
1	9,183	7845	0,877
2	12,621	4322	0,483
3	12,920	1682	0,188
4	13,366	880619	98,452
Total		894467	100,000

24-2-2023 15:03:52

HPLC data of 6u

Data Report

HPLC

Sample ID	: ANG_078	Tray	: 1
Date	: 23-2-2023	Vial	: 10
Data File Name	: ANG_078__019.lcd	Inj. Volume	: 10
Method File Name	: gradient_1mL_10-90%B_30C_15min_HPLC2.lcm	Flow	: 1.0 mL/min
Report File Name	: MIF inhibitors	Temperature	: 30°C
Batch File	: 23022023 MIF-AIF inhibitors.lcb	Detection	: UV 254 nm
Column	: Kinetex 5um EVO C18 100A, 150x4.6mm		
Mobile phase A	: Water		
Mobile phase B	: Acetonitrile		
Gradient	: Acetonitrile 10-90%, 15 min		
Column nr	: 00F-4633-E0		
Serial nr	: 745950-4		
Supplier	: Phenomenex		

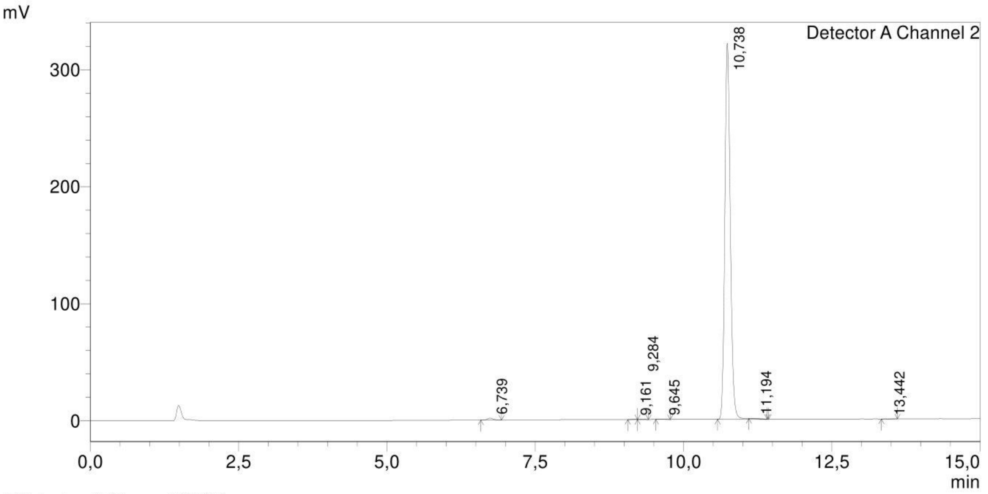

1 Detector A Channel 2/254nm

PeakTable

Detector A Channel 2 254nm

Peak#	Ret. Time	Area	Area %
1	6,739	11293	0,539
2	9,161	1464	0,070
3	9,284	1260	0,060
4	9,645	1965	0,094
5	10,738	2074817	99,021
6	11,194	3235	0,154
7	13,442	1289	0,062
Total		2095324	100,000

24-2-2023 14:42:27

HPLC data of 6v

University of Groningen Dept. Pharmaceutical Biology Groningen Research Institute of Pharmacy

Data Report

HPLC

Sample ID	: MKA_027	Tray	: 1
Date	: 24-2-2023	Vial	: 17
Data File Name	: MKA_027__033.lcd	Inj. Volume	: 10
Method File Name	: gradient_1mL_10-90%B_30C_15min_HPLC2-Print.lcm	Flow	: 1.0 mL/min
Report File Name	: MIF inhibitors	Temperature	: 30°C
Batch File	: 23022023 MIF-AIF inhibitors-print.lcb	Detection	: UV 254 nm
Column	: Kinetex 5um EVO C18 100A, 150x4.6mm		
Mobile phase A	: Water		
Mobile phase B	: Acetonitrile		
Gradient	: Acetonitrile 10-90%, 15 min		
Column nr	: 00F-4633-E0		
Serial nr	: 745950-4		
Supplier	: Phenomenex		

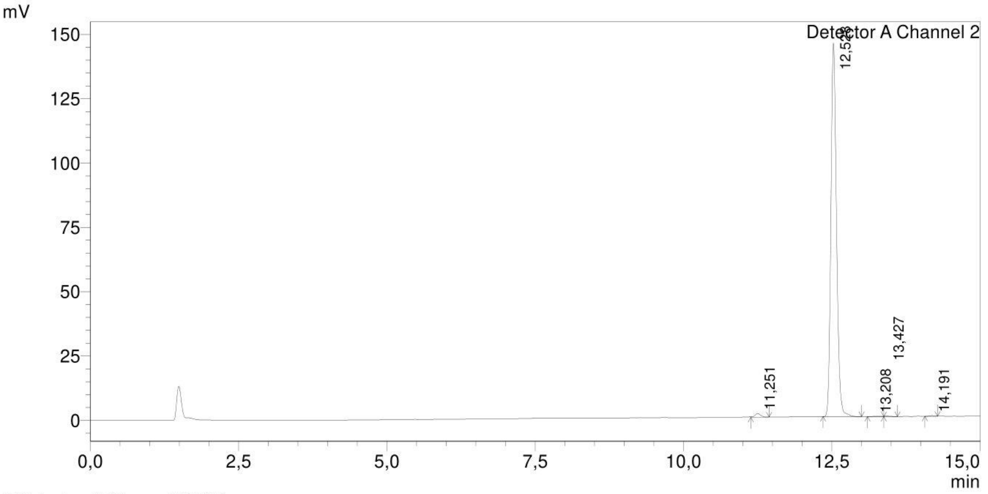

1 Detector A Channel 2/254nm

PeakTable

Detector A Channel 2 254nm

Peak#	Ret. Time	Area	Area %
1	11,251	9540	0,978
2	12,528	961686	98,553
3	13,208	1473	0,151
4	13,427	1295	0,133
5	14,191	1814	0,186
Total		975807	100,000

24-2-2023 15:00:22

HPLC data of 6w

Data Report

HPLC

Sample ID	: ANG_122	Tray	: 1
Date	: 24-2-2023	Vial	: 26
Data File Name	: ANG_122__051.lcd	Inj. Volume	: 10
Method File Name	: gradient_1mL_10-90%B_30C_15min_HPLC2-Print.lcm	Flow	: 1.0 mL/min
Report File Name	: MIF inhibitors	Temperature	: 30°C
Batch File	: 23022023 MIF-AIF inhibitors-print.lcb	Detection	: UV 254 nm
Column	: Kinetex 5um EVO C18 100A, 150x4.6mm		
Mobile phase A	: Water		
Mobile phase B	: Acetonitrile		
Gradient	: Acetonitrile 10-90%, 15 min		
Column nr	: 00F-4633-E0		
Serial nr	: 745950-4		
Supplier	: Phenomenex		

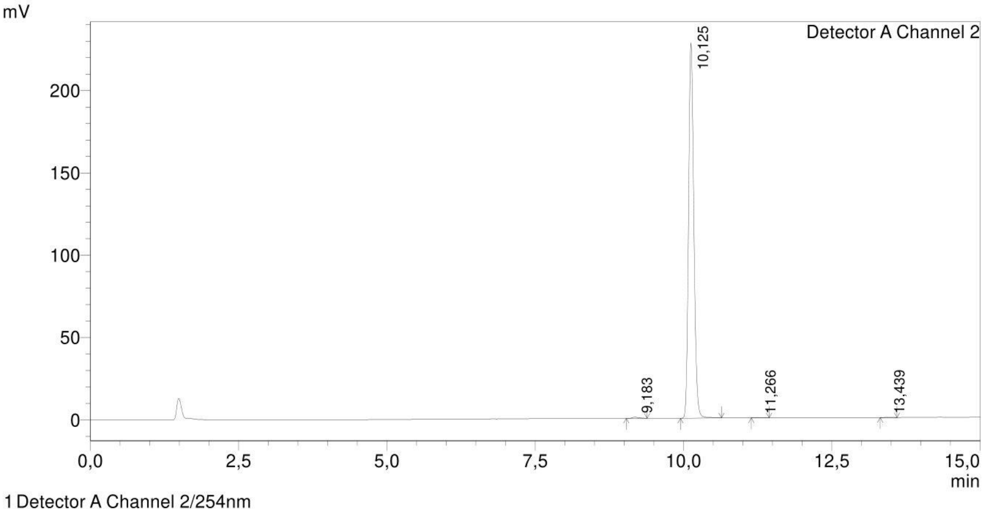

1 Detector A Channel 2/254nm

PeakTable

Detector A Channel 2 254nm

Peak#	Ret. Time	Area	Area %
1	9,183	5651	0,394
2	10,125	1426023	99,386
3	11,266	1790	0,125
4	13,439	1375	0,096
Total		1434839	100,000

24-2-2023 15:01:23

HPLC data of 6x

Data Report

HPLC

Sample ID	: MKA_019
Date	: 24-2-2023
Data File Name	: MKA_019__037.lcd
Method File Name	: gradient_1mL_10-90%B_30C_15min_HPLC2-Print.lcm
Report File Name	: MIF inhibitors
Batch File	: 23022023 MIF-AIF inhibitors-print.lcb
Column	: Kinetex 5um EVO C18 100A, 150x4.6mm
Mobile phase A	: Water
Mobile phase B	: Acetonitrile
Gradient	: Acetonitrile 10-90%, 15 min
Column nr	: 00F-4633-E0
Serial nr	: 745950-4
Supplier	: Phenomenex

Tray	: 1
Vial	: 19
Inj. Volume	: 10
Flow	: 1.0 mL/min
Temperature	: 30°C
Detection	: UV 254 nm

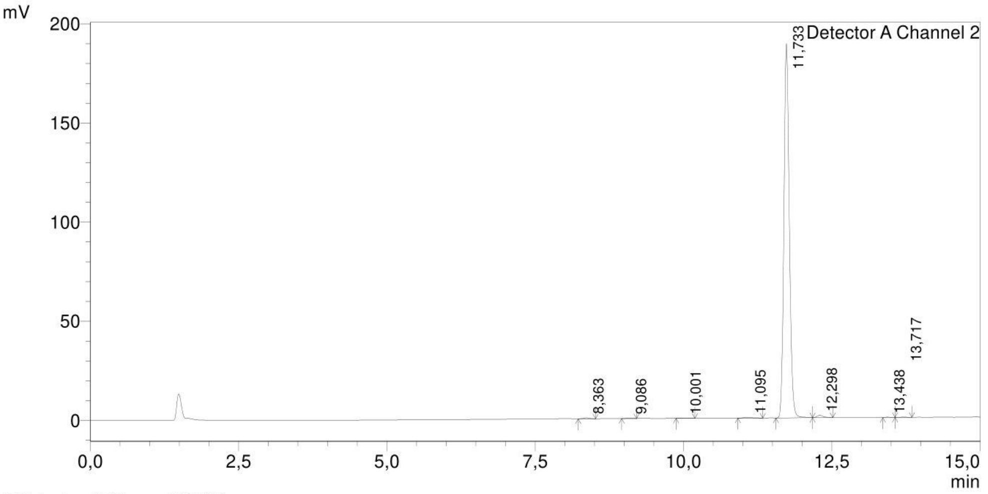

1 Detector A Channel 2/254nm

PeakTable

Detector A Channel 2 254nm

Peak#	Ret. Time	Area	Area %
1	8,363	2612	0,215
2	9,086	1106	0,091
3	10,001	1199	0,098
4	11,095	3330	0,273
5	11,733	1198461	98,436
6	12,298	8483	0,697
7	13,438	1136	0,093
8	13,717	1180	0,097
Total		1217508	100,000

24-2-2023 15:00:05

HPLC data of 6y

University of Groningen Dept. Pharmaceutical Biology Groningen Research Institute of Pharmacy

Data Report

HPLC

Sample ID	: MKA_031	Tray	: 1
Date	: 24-2-2023	Vial	: 24
Data File Name	: MKA_031__003.lcd	Inj. Volume	: 10
Method File Name	: gradient_1mL_10-90%B_30C_15min_HPLC2.lcm	Flow	: 1.0 mL/min
Report File Name	: MIF inhibitors	Temperature	: 30°C
Batch File	: 23022023 MIF-AIF inhibitors.lcb	Detection	: UV 254 nm
Column	: Kinetex 5um EVO C18 100A, 150x4.6mm		
Mobile phase A	: Water		
Mobile phase B	: Acetonitrile		
Gradient	: Acetonitrile 10-90%, 15 min		
Column nr	: 00F-4633-E0		
Serial nr	: 745950-4		
Supplier	: Phenomenex		

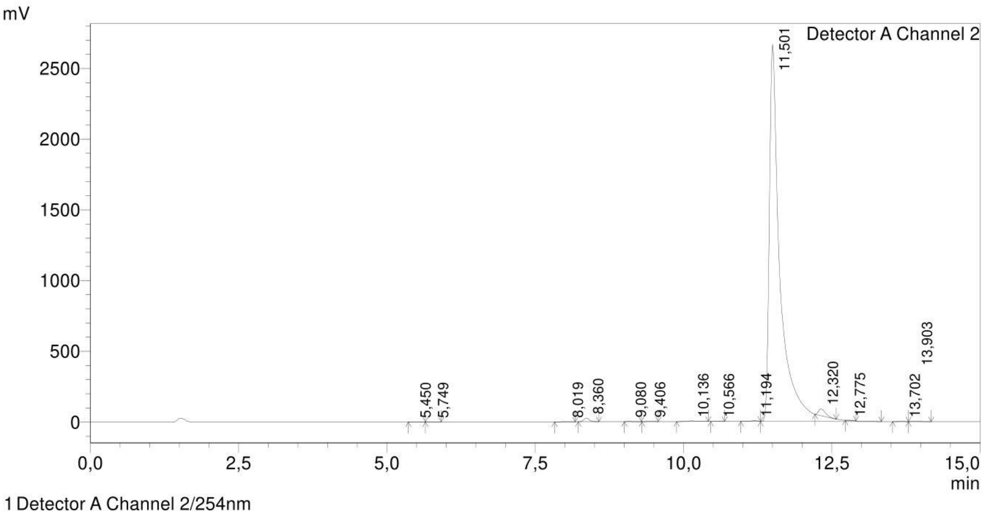

1 Detector A Channel 2/254nm

PeakTable

Detector A Channel 2 254nm

Peak#	Ret. Time	Area	Area %
1	5,450	5378	0,015
2	5,749	7202	0,021
3	8,019	17754	0,051
4	8,360	162376	0,465
5	9,080	4612	0,013
6	9,406	13654	0,039
7	10,136	40391	0,116
8	10,566	17067	0,049
9	11,194	48887	0,140
10	11,501	34215020	97,897
11	12,320	371187	1,062
12	12,775	10368	0,030
13	13,702	6275	0,018
14	13,903	29828	0,085
Total		34950000	100,000

27-2-2023 13:01:06

4. Reference

(1) Kozakov, D.; Brenke, R.; Comeau, S. R.; Vajda, S. PIPER: An FFT-Based Protein Docking Program with Pairwise Potentials. *Proteins Struct. Funct. Genet.* **2006**, *65* (2), 392–406. https://doi.org/10.1002/prot.21117.

(2) Chuang, G. Y.; Kozakov, D.; Brenke, R.; Comeau, S. R.; Vajda, S. DARS (Decoys As the Reference State) Potentials for Protein-Protein Docking. *Biophys. J.* **2008**, *95* (9), 4217–4227. https://doi.org/10.1529/biophysj.108.135814.
